# Application of a novel schema for describing fossil fern foliage and investigating taxonomic and morphological change across the Cretaceous–Paleogene boundary in western North America

**DOI:** 10.1002/ajb2.70243

**Published:** 2026-07-30

**Authors:** Fern B. Holian, Emily B. Sessa, Richard S. Barclay, Regan E. Dunn, Jacquelyn L. Gill, Kirk R. Johnson, S. Augusta Maccracken, Ian M. Miller, Jarmila Pittermann, Ellen D. Currano

**Affiliations:** ^1^ Department of Geology and Geophysics University of Wyoming Laramie 82071 WY USA; ^2^ New York Botanical Garden Bronx 10458 NY USA; ^3^ Department of Paleobiology National Museum of Natural History, Smithsonian Institution Washington 20560 DC USA; ^4^ Natural History Museums of Los Angeles County, La Brea Tar Pits Los Angeles 90036 CA USA; ^5^ Climate Change Institute, School of Biology and Ecology, University of Maine Orono 04469 ME USA; ^6^ Smithsonian National Museum of Natural History Washington 20560 DC USA; ^7^ Department of Earth Sciences Denver Museum of Nature & Science Denver 80205 CO USA; ^8^ National Geographic Society Washington 20036 DC USA; ^9^ Department of Ecology and Evolutionary Biology at the University of California Santa Cruz Santa Cruz 95060 CA USA; ^10^ Department of Botany University of Wyoming Laramie 82071 WY USA

**Keywords:** Cretaceous–Paleogene (K–Pg) boundary, fern spore spike, ferns, fossil fern descriptions, herbivory and pathogen damage, mass extinction, morphotype, taxonomy, turnover

## Abstract

**Premise:**

The Chicxulub asteroid impact at the Cretaceous–Paleogene (K–Pg) boundary triggered a mass extinction 66 million years ago. Ferns thrived in the aftermath of the extinction, evidenced by the well‐documented “fern spore spike.” However, fern macrofossil records across the K–Pg boundary are less understood.

**Methods:**

We analyzed 698 fern macrofossils from 19 Cretaceous and 83 Paleogene localities in the Raton (Colorado and New Mexico), Denver (Colorado), and Williston basins (North Dakota and Montana), grouping specimens into morphotypes using a newly developed descriptive schema. We examined familial affiliations and documented taxonomic and morphological turnover across the boundary. Additionally, herbivory and pathogen damage were categorized on each specimen.

**Results:**

We identified 33 morphotypes: eight were found only in the Cretaceous, 12 in both the Cretaceous and Paleogene, and 13 only in the Paleogene. Two fern families were in the Cretaceous only, nine in both the Cretaceous and Paleogene, and five in the Paleogene only. Taxa that occur in both the Cretaceous and Paleogene occupied less morphospace than taxa that occur only in the Cretaceous or the Paleogene. Sixty‐three specimens had herbivory or pathogen damage; one damage type was found in the Cretaceous only, nine in both the Cretaceous and Paleogene, and seven in the Paleogene only.

**Conclusions:**

Our data indicate possible changes in morphotype composition, family representations, morphological disparity, and insect herbivory across the K–Pg boundary. This research is important for understanding K–Pg recovery dynamics, fern adaptation, and their relation to deep time and modern catastrophic events.

A remarkable aspect of the Cretaceous–Paleogene (K–Pg) mass extinction is the resilience of ferns (Orth et al., 1981). Ferns not only withstood the effects of the Chicxulub asteroid impact, which struck the Yucatan Peninsula in Mexico 66 million years ago (Ma), but thrived in its aftermath. Often considered “disaster taxa” (Thomas and Cleal, 2022; Azevedo‐Schmidt et al., 2024), ferns are well adapted to thrive after catastrophic events. Ferns are thought to have been the first plants to recolonize the devastated post‐K–Pg landscape and quickly rose to dominance. This resurgence was recorded as a “fern spore spike,” an abrupt rise in the ratio of fern spores to angiosperm pollen in the palynological record (Tschudy, 1984). The fern spike occurs in sediments immediately above the K–Pg boundary, which is identified by distinct geochemical and geophysical markers, particularly an iridium anomaly (Alvarez et al., 1980) and the presence of impact spherules and shocked quartz grains (Smit and Klaver, 1981). This globally recognized phenomenon has been observed in North America (Pillmore et al., 1984; Tschudy, 1984; Nichols and Fleming, 2002), Europe (Brinkhuis and Schiøler, 1996), Japan (Saito et al., 1986), and New Zealand (Vajda et al., 2001).

This pattern of rapid fern recolonization following major disturbances is hypothesized to have been influenced by the diversity of fern reproductive strategies. Unlike many angiosperms, which often rely on insect pollinators (especially ancestrally; Stephens et al., 2023), ferns reproduce via wind‐dispersed spores, enabling them to recolonize environments even when pollinator populations have collapsed. Different fern species have a diversity of modes of asexual reproduction, including apogamy (the production of diploid spores), vegetative budding, and rhizomatous growth, which also allow for rapid reproduction when populations are low. In addition to their reproductive advantages in colonizing disturbed environments (Thomas and Cleal, 2022), ferns are physiologically well suited to post‐disturbance conditions, thriving in environments with low nutrient availability, limited light, and high humidity (Sharpe et al., 2010). By rapidly recolonizing disturbed landscapes, ferns may also play a role in facilitating the recovery of other species by stabilizing soils, increasing soil moisture, and replenishing nutrients (Azevedo‐Schmidt et al., 2024).

These reproductive and physiological adaptations are hypothesized to have enabled the success of ferns in the earliest Paleogene, which was characterized by an extended period of harsh environments. The Chicxulub impact caused a series of catastrophic events, including earthquakes, tsunamis, wildfires, and shock waves leading to widespread deforestation (Alvarez et al., 1980; Sweet, 2001; Morgan et al., 2022; Gulick, 2024). In the immediate aftermath, insolation was significantly reduced as particles and debris were ejected into the atmosphere, blocking the sun's rays and causing an “impact winter” in which Earth's surface temperatures decreased worldwide; this effect may have lasted for several months to decades (Vajda et al., 2001; Vellekoop et al., 2014, 2016). Acidification of rain by impact ejecta added another layer of ecological stress (Schulte et al., 2010). Plants experienced a decline in species diversity as a result of these stressors. The macrofossil record from the Williston Basin in North Dakota indicates a loss of around 57% of species (Wilf and Johnson, 2004; Wilf et al., 2023). In Patagonia, extinction rates exceeded 90% for non‐monocotyledonous angiosperms (Stiles et al., 2020), while in Colombia, there was a 45% decrease in palynomorph diversity (Carvalho et al., 2021). Ferns were likely able to quickly recolonize the deforested landscapes as competitors declined, while decreased insolation, cooler temperatures, and acidified soils further contributed to the decline in pollinators and animal‐pollinated plants. Despite the widespread recognition of the fern spore spike, paleobotanical research on the K–Pg extinction and recovery has primarily focused on non‐monocotyledonous angiosperm macrofossils (e.g., Wilf and Johnson, 2004; Wilson Deibel et al., 2024). Fern macrofossils represent an important opportunity to understand the taxonomy, physiology, and ecology behind the fern spore spike.

The fern lineage has been estimated to have diverged from its sister group 346.7 Ma based on molecular data from extant and fossil calibrations (Pelosi et al., 2022). Phylogenetic research has established key patterns of fern diversification beginning in the Cretaceous and continuing throughout the Cenozoic, which is the interval most relevant to this study (Schuettpelz and Pryer, 2009; Testo and Sundue, 2016). Leptosporangiate ferns experienced a radiation event at the beginning of the Cretaceous, likely associated with the rise of angiosperms (Schuettpelz and Pryer, 2009). This diversification may have been associated with an ecological opportunistic response as angiosperm‐dominated ecosystems provided new niches for ferns to exploit. Bursts of fern diversification occurred throughout the Cenozoic, likely driven by the evolution of epiphytism, an adaptation that allowed ferns to expand into tropical rainforest canopies (Schuettpelz and Pryer, 2009). The stem molecular ages of most fern families predate the K–Pg boundary (66.04 Ma; with the exception of Davalliaceae [66 Ma] and Polypodiaceae [56 Ma]; Pelosi et al., 2022). This pattern suggests that the majority of extant fern families diverged from one another before the K–Pg extinction.

Systematic approaches to identifying, describing, and classifying fern macrofossils, including delineating morphotypes and proposing familial assignments, are necessary to understand floristic changes at the K–Pg boundary and the fern spore spike. To address this critical knowledge gap, we first developed a standardized schema for describing fossilized fern remains. This framework can also be used for modern specimens, allowing for more rigorous comparisons between fossil and extant taxa. We then used this schema to describe fern macrofossil morphotypes, i.e., groups defined by shared morphological characteristics, from before and after the K–Pg boundary in the Raton (Colorado and New Mexico), Denver (Colorado), and Williston (North Dakota and Montana) basins. Fern macrofossils are relatively well preserved and abundant in these basins, but have not been thoroughly investigated, and taxonomic identifications are limited (but see Knowlton, 1930; Brown, 1962; Collinson, 2001; Manchester, 2014; Berry, 2021). We evaluated previous taxonomic identifications and, when possible, propose familial assignments for each morphotype. We also used morphological data to investigate changes in morphospace occupancy across the K–Pg boundary and to propose foliar characters tied to success in the aftermath of the impact. Last, we recorded herbivory and pathogen damage because these can serve as proxies for understanding ecological interactions and environmental stressors that may have shifted as a result of the Chicxulub bolide impact. Together, our findings provide insights into fern turnover and the ecological consequences of a mass extinction event. Identifying which taxonomic groups thrived during the aftermath of the K–Pg impact and what foliar characters these groups share will also allow us to better predict and understand how ferns may respond to modern‐day catastrophes, including rapid environmental changes, substantial losses in biodiversity, and consequential ecological disruptions.

## MATERIALS AND METHODS

### Geologic setting

**Figure 1:**
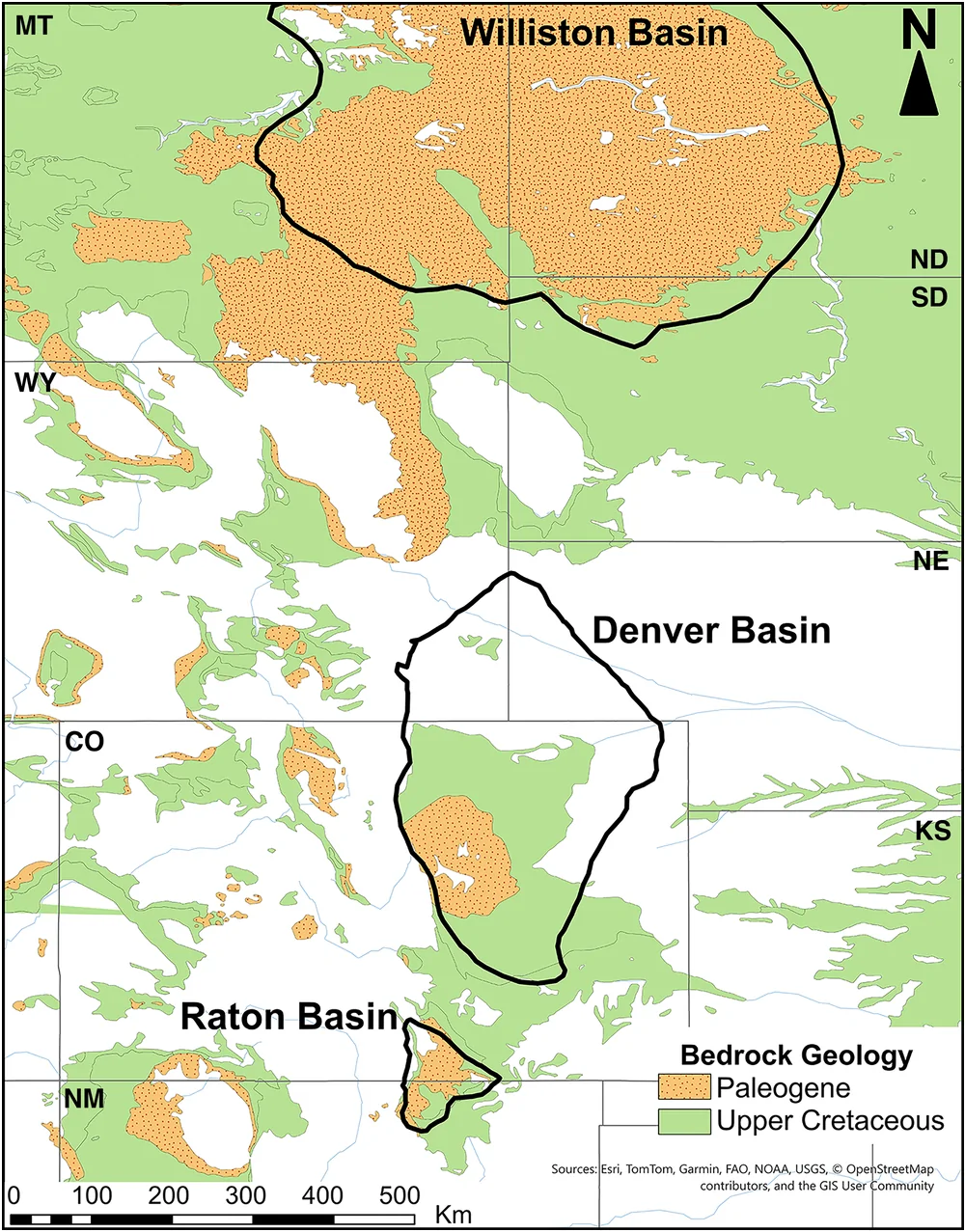
Map of study area. Western United States with outlined Williston Basin, Denver Basin, and Raton Basin. Map adapted from Coleman and Cahan (2012), Commission for Environmental Cooperation (2022), and Garrity and Soller (2009).

We analyzed 786 fern fossils from the Denver (Colorado, USA), Williston (North Dakota and Montana, USA), and Raton (Colorado and New Mexico, USA) Basins (Figure 1) that are held in the Burke Museum of Natural History and Culture (Seattle, WA, USA; examined in photographs), the Denver Museum of Nature & Science (Denver, CO, USA; examined in person), the University of Wyoming Geological Museum (Laramie, WY, USA; examined in person), and the Yale Peabody Museum (New Haven, CT, USA; examined in person). These basins contain the K–Pg boundary defined by an iridium anomaly and the subsequent fern spore spike recorded immediately above the boundary. This data set included 197 specimens from Cretaceous‐aged (Maastrichtian) rocks, 540 specimens from the Paleogene (Danian), and 49 specimens with an unknown, but likely Maastrichtian or Paleocene, age. We opted to include the unknown age specimens in this study to obtain a more comprehensive understanding of each morphotype, but were cautious in including these in statistical analyses. Cretaceous fossils were collected from 24 localities (19 had specimens that could be placed into a morphotype), and Paleogene fossils from 90 localities (83 had specimens that could be placed into a morphotype). Geographic location, age, stratigraphic position, and depositional environment for each locality are given in Appendix S1 (Table S1), and locality and repository information for each specimen is provided in Appendix S1 (Tables S2 and S3).

In the Denver Basin, the K–Pg boundary is located within the Denver Formation (Barclay et al., 2003). The depositional environment of this formation comprises coarse‐grained, sand‐rich alluvial fan and debris flow deposits in proximal mountain settings, transitioning to finer‐grained fluvial, paludal, and lacustrine sediments in distal, low‐energy environments at the center of the basin. The K–Pg boundary itself is marked by a 3‐cm‐thick claystone layer that exhibits the iridium anomaly and shocked quartz grains, as observed in the West Bijou Site (Barclay and Johnson, 2004). Directly above the boundary, fern spores comprise 74% of the palynological assemblage in contrast to 5% of the assemblage in the Cretaceous. Their abundance declines to 8% of the assemblage 7 cm above the boundary. Fossils examined here come from the Corral Bluffs (Lyson et al., 2019), West Bijou (Barclay et al., 2003), and Plum Creek Parkway (Ellis et al., 2003) study areas.

Within the Williston Basin, the K–Pg boundary is associated with the transition between the Hell Creek Formation and the Fort Union Formation (Johnson, 1992). The Hell Creek Formation is late Maastrichtian (latest Cretaceous) and is composed of mudstone, lenticular sandstone, and muddy sandstone. The depositional environment includes a fluvial system with channel point bars and oxbow lakes. This poorly drained floodplain is indicated by root traces, ped structures, and slickensides. The Fort Union Formation represents the earliest Paleocene and consists of flat‐laminated mudstone, lignite, and tabular or lenticular sand bodies. This section shows a transition to swampy, fluvio‐deltatic sediments with ponds, crevasse splays, and meandering channels. A high abundance of fern spores is observed directly above the boundary clay, which exhibits the iridium anomaly and shocked quartz (Bercovici et al., 2012). Notable contributors of the fern spike include *Cyathidites* (59% of the total assemblage) and *Laevigatosporites* (18.8%). North Dakota specimens were collected as part of larger paleobotanical studies by Johnson (2002) and Wilf and Johnson (2004), and Montana specimens were previously studied by Wilson et al. (2021) and Wilson Deibel et al. (2024).

For the Raton Basin, the K–Pg boundary occurs in the lower Raton Formation (Pillmore and Flores, 1987). This formation is composed of sandstone, siltstone, mudstone, coal, carbonaceous shale, and conglomerate deposited in swamp, floodplain, and meandering‐channel systems. Orth et al. (1981) report the K–Pg boundary with the iridium anomaly at the base of a coal bed along with the pronounced increase in fern spores directly above the boundary. Specimens were collected from the Berwind Canyon (Colorado), Clear Creek South (Colorado), North Ponil (New Mexico), and Starkville South (Colorado) K–Pg sections of Pillmore et al. (1984, 1999) and Pillmore and Flores (1987), and on the Vermejo Park Ranch (New Mexico).

Depositional environment likely affects the composition, preservation potential, and spatial and temporal resolution of fossil floras. We, therefore, attempted to classify the depositional environment of each fossil locality in our study (Appendix S1: Table S1), although we could not always do so given our use of museum collections with heterogeneous locality data. The Denver, Hell Creek, Fort Union, and Raton formations all represent fluvial depositional systems. As done in previous work in the Williston Basin by Johnson (2002) and Wilson Deibel et al. (2024), we categorized localities as “channel” or “overbank” based on lithology and sedimentary structures. Channel deposits are characterized by coarser‐grained rocks, interbedded sandstones and silt/mudstones, inclined bedding, and greater oxidation. Overbank deposits (e.g., crevasse splays, abandoned channels, ponds, backswamps) typically have finer‐grained rocks, massive or tabular horizontal beds, and less oxidation (Fastovsky, 1987; Johnson, 2002; Wilson Deibel et al., 2024).

### Schema for describing fossilized ferns

We developed a standardized method for describing fossilized fern morphotypes by scoring a list of morphological characters and character states. This method provides a consistent framework for description, including for incomplete specimens, that can be applied to the majority of fern fossils. We comprehensively reviewed the literature for modern and fossilized ferns to compile a list of characters that can describe most features observed in fern fronds. Critical references for this work include *Flora of North America North of Mexico* (Cranfill, 2020a, 2020b, 2020c; Farrar, 2020; Johnson, 2020; Kato, 2020; Montgomery and Wagner, 2020; Moran, 2020; Nauman, 2020a, 2020b, 2020c; Nauman and Evans, 2020; Smith, 2020a, 2020b; Wagner et al., 2020; Whetstone and Atkinson, 2020; Yatskievych, 2020; Yatskievych and Windham (2020), He and Kato (2013), Jin et al. (2013), Tryon (1960), Wang et al. (2013), Zhang et al. (2013), and Zhang and Nishida (2013). The complete description formula of these characters is as follows: Preserved segment division (to the highest order observed), complete blade division, ultimate segment shape, ultimate segment tapering at the tip, ultimate segment tapering at the base, ultimate segment symmetry, penultimate segment shape, penultimate segment tapering at the tip, penultimate segment tapering at the base, penultimate segment symmetry, tertiary ultimate segment shape, tertiary ultimate segment tapering at the tip, tertiary ultimate segment tapering at the base, quaternary ultimate segment shape, quaternary ultimate segment tapering at the tip, quaternary ultimate segment tapering at the base, complete frond shape, frond tapering at the tip, frond tapering at the base, vein type, vein termination at the margin, vein termination location, margin type, reproductive structure type, shape of sori/sporangial groupings or other reproductive structures, sori/sporangial or other reproductive structure placement, and presence of indusia/indusium type. Definitions, descriptions, and illustrations of each character and their character states are in Appendix S2.

We include all characters in each morphotype description to ensure fully comprehensive, parallel descriptions and avoid assumptions about characters that are not preserved. For example, if venation was not preserved in any fossils of a morphotype, then part of the description would state “venation not observed”. We also note when characters were not completely preserved, but their character state could be reasonably inferred. An example would be a large penultimate segment that is pinnate, but the complete penultimate segment is not preserved. Because of the size of the penultimate segment, it would be unreasonable to suggest that this penultimate segment would be further segmented into bipinnate or further division. In this instance, we suggest that the description state “complete blade division not observed but likely pinnate”. The division of the penultimate segment should also be explicitly stated in this instance as “pinnate penultimate segment”. We note that this scenario is common where it is often unknown if an entire frond and/or its complete blade division is preserved.

The terminology of ultimate, penultimate, tertiary ultimate, quaternary ultimate segments, and so forth is preferred over pinnulet, pinnule, and pinna when referring to segments of fossilized fern fronds because it ensures consistency between descriptions and accounts for cases where the entire blade division is not known. Further explanation of this terminology along with illustrations are provided in Appendix S2.

### Delineating and naming morphotypes

Characters were scored for each individual specimen. Specimens were divided into morphotypes based on consistency in character states scored, along with overall visual similarity. For this method, the majority of characters should be consistent between individuals of the same morphotype, with few exceptions. These exceptions are if character states are on a continuum or if certain characters are not observable in some individuals. For example, frond or segment shape may vary along a continuum within a morphotype where some individuals may exhibit lanceolate fronds or segments and others may have a more ovate appearance. In cases of unobserved characters, such as higher‐order segment divisions or reproductive structures, all other character states should be consistent for the morphotype. Ultimately, the process of morphotyping involves subjectivity. We chose to distinguish morphotypes as separate groups when the observed described character states were reasonably different. Conversely, we grouped morphotypes together when there were no clearly described distinctions in character states.

Morphotypes were numbered and sequentially ordered loosely based on their best‐supported hypothesis of familial taxonomic affiliation (see methods below). Families with generally stronger hypotheses were placed earlier in the morphotype list. Within each family, morphotypes that were supported by stronger family hypotheses were listed first.

The criteria for the morphotype quality index (MQI; Johnson, 1989; Arens and Allen, 2014), traditionally applied to non‐monocotyledonous angiosperms, were adapted for application to ferns to assess the completeness of a morphotype and to determine whether sufficient character states were preserved to warrant naming or placing the morphotype in a taxonomic group. The non‐monocotyledonous angiosperm method focuses on the exemplar quality index (EQI) of individual specimens (Wilson et al., 2021), with MQI reflecting the number of well‐preserved specimens. For ferns, we propose a different approach. Because entire fern fronds can be much larger than non‐monocotyledonous angiosperm leaves, along with the fact that some fern fronds are fertile, we suggest that morphotype completeness should be assessed holistically rather than by evaluating individual specimens. Ultimately, our method does not count the number of complete specimens but rather assesses our ability to completely reconstruct the morphology of the fern frond. The criteria to assess MQI are as follows and are scored using a point system accordingly. With a total of four points, the classification for the quality of each morphotype is placed into high, medium, or low. A high MQI requires a score of 4 to 3.5, a medium is <3.5 to ≥2, and a low is <2. We acknowledge the considerable threshold for a high MQI, but each character listed must be evaluated for a complete and accurate reconstruction of the morphotype. The characters that require evaluated character states are as follows:

1. Reproductive structures. 1 point: Reproductive structures are observed. 0.5 point: Reproductive structures are not observed but can be inferred with confidence based on other characteristics. As an example, if a morphotype has consistent characteristics with modern aquatic ferns in Salviniales, it is assumed that the reproductive structures are contained within a sporocarp and not on the fern frond, and reproductive character states can be reasonably inferred, although they are not fully described and all associated reproductive character states are not assumed. 0 points = Reproductive structures are not observed and cannot be inferred with confidence. This category includes hypothesized dimorphic fronds where character states of the fertile fronds and reproductive structures are not reasonably inferred.
2. Complete blade division. 1 point: The entire frond is preserved. 0.5 point: The entire frond is not preserved but can be inferred with confidence. For example, the frond is incomplete, but the preserved segments are sufficiently large making it unreasonable to assume that they are further divided. 0 points: The entire frond is not preserved and cannot be inferred with confidence.
3. Venation type. 1 point: The venation type can be assigned a character state. 0 points: The venation type cannot be assigned a character state.
4. Margin type. 1 point: The margin type can be assigned a character state. 0 points: The margin type cannot be assigned a character state.

After morphotyping was complete, example photos of each morphotype were sent to experts on modern ferns (Dr. Susan Fawcett, Dr. Robbin Moran, Dr. Alan Smith, Dr. Weston Testo, and Dr. Eddie Watkins), who were surveyed about which modern taxa the morphotypes most closely resembled. For nearly all morphotypes, the experts provided differing interpretations, reflecting disagreement in assessment, and each hypothesis was thoroughly investigated. Taxonomic names and affinities proposed in the literature were also examined. However, we did not incorporate character descriptions of established morphotypes from the literature when writing our descriptions and diagnostic characters; these are based solely on observed characters in our fossil data set. We investigated each morphotype by comparing their morphology to modern photographs of the proposed corresponding taxa sourced from Moran (n.d.) and modern descriptions of families.

Morphotypes were subsequently placed into families. Genera with strong similarities to the morphotypes are denoted as “reference genera.” However, our hypotheses do not suggest that the morphotypes should be placed in the stated genera, due to the limited confidence in assigning extant genera to fossils from deep time.

The stem molecular ages of each family were referenced (Pelosi et al., 2022) to provide an approximate framework for evaluating whether lineages could plausibly have been present at the K–Pg boundary. If a suggested family had a stem molecular age of 66 million years (My) or younger, the hypothesis was still investigated and assessed more critically for morphological similarity, but all were rejected. Hypotheses for each morphotype's taxonomic placement were sorted into four categories: strong, moderate, weak, and rejected. The criteria for each category are listed in Table 1 and include the presence of diagnostic features, the number of inconsistent features, and the minimum MQI required. All criteria must be met for the hypothesis to be placed in each distinct category.

**Table 1:** Variables for fern morphotype family‐level hypothesis strength. The criteria listed for diagnostic features, inconsistent features, and morphotype quality index must all be met to fit each hypothesis strength.

Hypothesis strength	Diagnostic features	Inconsistent features	Morphotype quality index
Strong	Must include at least one unique diagnostic feature or a unique combination of diagnostic features exclusive to the family.	May not have more than one minor inconsistent feature with the family as a whole.	Medium to High
Moderate	A diagnostic feature or a combination of diagnostic features is present but is not unique to one family. The hypothesis has solid grounding, but other families with similar features cannot be confidently excluded.	May not have more than one minor inconsistent feature with the family as a whole.	Low to High
Weak	No diagnostic features unique to a family are present.	May not have more than one minor inconsistent feature with the family as a whole.	Low to High
Rejected	No diagnostic features unique to a family are present.	One or more major features are inconsistent with the family as a whole.	Low to High

Best‐supported familial hypotheses were assigned for each morphotype in our study. The assigned hypothesis represents the highest strength hypothesis (strong, moderate, weak) per morphotype, but can reflect any confidence level. For example, even if no family received a rating of “strong” for a particular morphotype, we still selected what would be the best‐supported hypothesis from among the suggested families for that morphotype.

All named morphotypes in this study reflect names previously established in the literature, with no new names proposed. Morphotype nomenclature is primarily based on Knowlton (1930), with additional references from Brown (1962) and Rothwell and Stockey (1994). Some morphotypes have been renamed over time, reflecting efforts to better represent taxonomic affiliations. As such, some morphotypes have been referred to by multiple names in the literature. For the sake of consistency, we have opted to prioritize names established by Knowlton (1930), supplementing with the later sources as necessary. In cases where published descriptions of morphotypes are ambiguous or there is uncertainty regarding whether our morphotypes matched the published morphotypes, we used names based on the best morphological match.

Unnamed morphotypes represent those that have not yet been formally described or assigned a name. We have elected not to name these morphotypes due to uncertainty in both their genus‐level and higher‐level taxonomic affiliations. Because the best‐supported hypotheses vary in strength, assigning new names at this stage could lead to future misrepresentation of these morphotypes.

### Morphospace analysis

We described variations in fern morphology and disparity across the K–Pg boundary using modifications of the methods of Stiles et al. (2020), which considered morphological change in non‐monocotyledonous angiosperms across the K–Pg boundary in Patagonia. Character‐state data recorded in Appendix S1 (Table S4) were modified for morphological analyses as follows. First, we eliminated Fern33 (*Equisetum* sp.) from the analysis because none of our specimens included vegetative material. Second, the character “preserved segment division” represents a mix of the true complete blade division and preservational completeness. To separate these factors, we subdivided this character into ultimate segment division, penultimate segment division, tertiary ultimate segment division, and quaternary ultimate segment division. Third, segment shape is often variable within a morphotype, and so we scored each morphotype for the presence/absence of each segment shape. All other characters were retained as categorical variables. Since only one morphotype displays multiple states for variable margin type (Fern21) and reproductive structure shape/type (Fern12), we chose to use the most common character state in the analysis, rather than score all morphotypes for presence/absence of each character state as done for segment shape. Fourth, we eliminated characters that could not be scored on at least one third of morphotypes or that had the same character state for all morphotypes: complete blade division; tertiary ultimate segment shape, tapering at tip, and tapering at base; quaternary ultimate segment division, shape, tapering at tip, and tapering at base; frond shape, tapering at tip, and tapering at base; vein termination at the margin; and indusium type. Last, the remaining data matrix contains a total of 11 character states that were thought to be likely although not directly observed (i.e., character states with a question mark in Appendix S1: Table S4). We assumed that these are correct for the morphological analysis because we were cautious in making these designations and they represent just 2.8% of the total character states scored in our final matrix.

Morphological analyses were conducted using R version 4.5.2 (R Core Team, 2025). Gower's distance metric (function gowdis in the R package FD; Laliberté et al., 2014) was used to calculate pairwise distances among morphotypes because it can handle both categorical and asymmetric binary variables and is robust against missing data (Gower, 1985). The distance matrix was visualized using a weighted principal coordinates analysis (PCoA) with the function wcmdscale in the R package vegan (Oksanen et al., 2025). To investigate changes in morphological disparity through the study interval, we grouped the morphotypes based on whether they occur only in the Cretaceous, only in the Paleogene, or in both the Cretaceous and the Paleogene (also referred to as K–Pg taxa or boundary‐crossers). As done by Stiles et al. (2020), we calculated four disparity metrics for each of the three groups. First, a hypercuboid volume was estimated by multiplying the ranges of the first four axes of the PCoA (Wills et al., 1994). Second, the sum of ranges of the first four axes of the PCoA was calculated. Last, the average and maximum Gower pairwise distances were determined.

Because morphological analyses are sensitive to missing data, we conducted three sets of analyses with varying thresholds for missing data. The first and most inclusive analysis is referred to as “All Taxa” and includes all morphotypes and the character states described above. In the second analysis (Subset 1), we removed from the “All Taxa” matrix all morphotypes for which fewer than half of characters could be scored; this eliminated Fern12 (Pg), Fern13 (K–Pg), Fern17 (Pg), Fern21 (K–Pg), and Fern29 (Pg). For the third analysis (Subset 2), we returned to the “All Taxa” matrix, eliminated all character states that could not be scored for >60% of morphotypes (penultimate segment shape, penultimate segment symmetry, reproductive structure shape/type, and reproductive structure placement). Then, all morphotypes with fewer than 60% of the remaining characters were removed (Fern13, Fern21, and Fern29). The morphological matrices used and character state explanations are included in Appendix S1 (Tables S5–S8), and R code is included as Appendix S3.

### Assessment of herbivory and pathogen damage type

Insect herbivory was assessed by recording the functional feeding groups and corresponding damage types (DTs) on each fossil specimen as done by Labandeira et al. (2007 and subsequent updates) for images and descriptions of DTs. In addition, pathogen and fungal DTs were recorded. Herbivory and pathogen DTs were analyzed by compiling a list of DTs observed on each morphotype and calculating the percentage of specimens with damage per morphotype. Additionally, we documented changes in the number and composition of damage types and functional feeding groups observed before and after the boundary. Because the fossils examined in this study came from museum collections, rather than census collections in which every specimen from a site is identified and scored for herbivory, we did not pursue further quantitative analyses.

## RESULTS

### Morphotype and familial affiliation results

**Figure 2:**
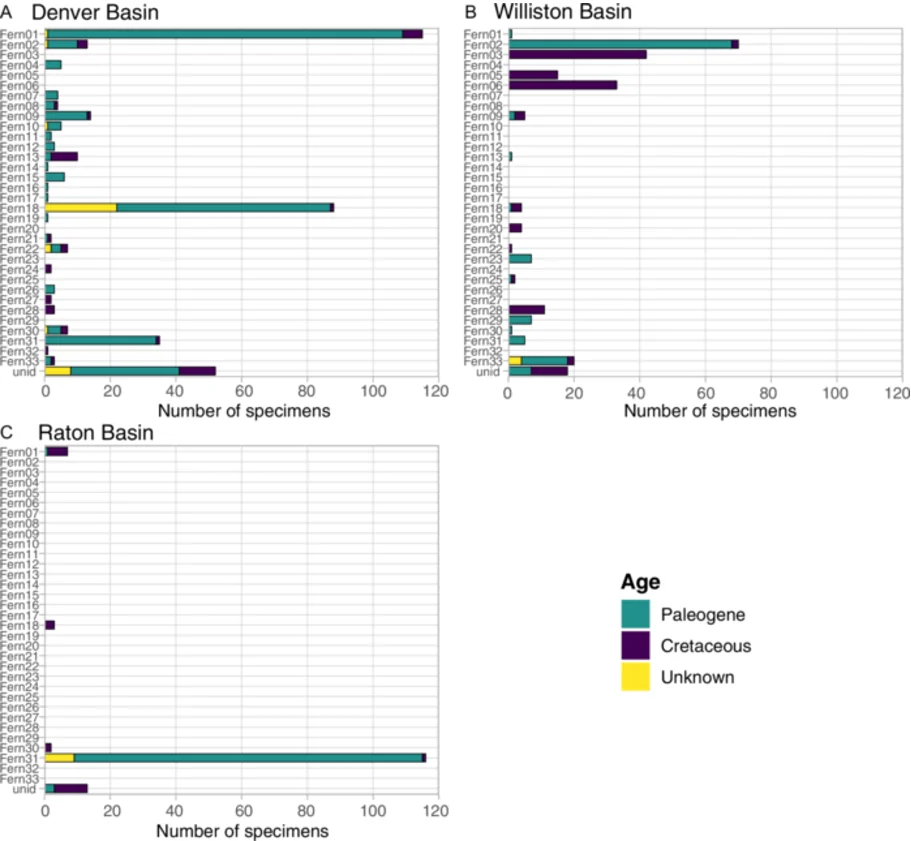
Fern morphotype abundances for each basin (see Figure 1). Number of specimens per morphotype from the (A) Denver Basin, (B) Williston Basin, and (C) Raton Basin. Bar fill color indicates the geological age of specimens associated with each morphotype.

Of the 786 fern fossils analyzed, 698 specimens were assigned to 33 distinct morphotypes. Character states and MQI for each morphotype are summarized in Appendix S1 (Table S4), and full descriptions, diagnostic characters, specimen information, and images are provided in Appendix S4. From our data, 24% (8/33) of morphotypes were exclusive to the Cretaceous, 39% (13/33) were exclusive to the Paleogene, and 36% (12/33) occurred in both time intervals. As such, 20 total morphotypes were observed in the Cretaceous, and 25 total morphotypes were observed in the Paleogene. Twenty‐six morphotypes were found in the Denver Basin (*N* = 390 specimens examined at 68 localities; Figure 2A), 17 in the Williston Basin (*N* = 247 at 29 localities; Figure 2B), and four in the Raton Basin (*N* = 141 at 20 localities; Figure 2C). Eight specimens had unknown basin information (see Appendix S1: Tables S2 and S3).

**Figure 3:**
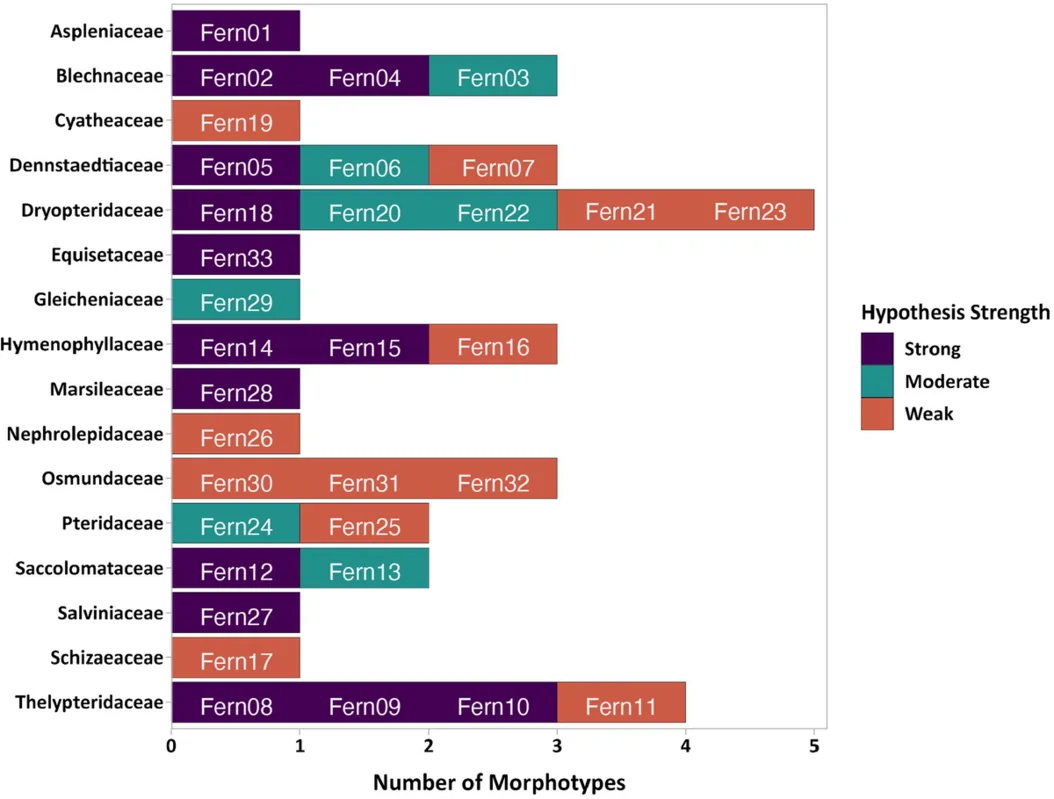
Morphotype best‐supported family hypotheses. Number of morphotypes assigned to each best‐supported family hypothesis. Morphotype numbers are shown within the bars; fill color indicates hypothesis strength.

Best‐supported family hypotheses are summarized in Figure 3 and are color‐coded by hypothesis strength. Table 2 outlines all of the hypotheses investigated per morphotype along with the strength of each hypothesis. Detailed discussion on how the strength of each hypothesis was determined is provided in Appendix S4. Based on our best‐supported family hypotheses, 16 total families were observed. Of these, two families were observed only in the Cretaceous, five only in the Paleogene, and nine in both time intervals. Fifteen families were observed in the Denver Basin, 11 in the Williston Basin, and three in the Raton Basin.

**Table 2:** Summary of morphotype family assignments. Fossil names are from previous publications, rather than our own suggestions. All hypotheses are compiled from literature sources and our own suggestions. Variables for hypothesis strengths are provided in Table 1.

Morphotype	Strong hypotheses	Moderate hypotheses	Weak hypotheses	Rejected hypotheses
Fern01 *Allantodiopsis erosa* (Lesquereux) Knowlton et Maxon	Aspleniaceae	–	Athyriaceae; Diplaziopsidaceae	Thelypteridaceae; Osmundaceae; Polypodiaceae
Fern02 *Onoclea hesperia* Brown; *Woodwardia latiloba serrata* Knowlton; *Woodwardia arctica* (Heer) RW Brown	Blechnaceae	–	–	Onocleaceae; Pteridaceae
Fern03	–	Blechnaceae; Onocleaceae	–	–
Fern04 *Salpichlaena anceps* (Lesquereux) Knowlton	Blechnaceae	–	–	–
Fern05 *Dennstaedtia crossiana* Knowlton	Dennstaedtiaceae	–	–	–
Fern06	–	Dennstaedtiaceae	Dryopteridaceae	–
Fern07	–	–	Dennstaedtiaceae; Dryopteridaceae; Anemiaceae	Davalliaceae
Fern08	Thelypteridaceae	–	Osmundaceae; Athyriaceae	–
Fern09	Thelypteridaceae	–	Cyatheaceae	–
Fern10	Thelypteridaceae	Dryopteridaceae	–	Nephrolepidaceae
Fern11	–	–	Thelypteridaceae; Gleicheniaceae	Athyriaceae; Osmundaceae
Fern12 *Saccoloma gardneri* (Lesquereux) Knowlton	Saccolomataceae	–	–	Pteridaceae; Dennstaedtiaceae
Fern13		Saccolomataceae	Pteridaceae	–
Fern14	Hymenophyllaceae	–	–	–
Fern15	Hymenophyllaceae	–	–	Pteridaceae
Fern16	–	–	Hymenophyllaceae	–
Fern17	–	–	Schizaeaceae;Hymenophyllaceae; Pteridaceae	–
Fern18 *Dryopteris lakesii* (Lesquereux) Knowlton	Dryopteridaceae	–	Cyatheaceae	Tectariaceae
Fern19	–	–	Cyatheaceae; Dryopteridaceae	–
Fern20 *Dryopteris integra* Knowlton	–	Dryopteridaceae	–	–
Fern21	–	–	Dryopteridaceae	Athyriaceae
Fern22	–	Dryopteridaceae; Saccolomataceae	Athyriaceae; Dennstaedtiaceae	–
Fern23	–	–	Dryopteridaceae; Athyriaceae	–
Fern24	–	Pteridaceae	–	–
Fern25	–	–	Pteridaceae	Osmundaceae
Fern26	–	–	Nephrolepidaceae; Pteridaceae	–
Fern27 *Salvinia* sp.	Salviniaceae	–	–	–
Fern28 *Hydropteris pinnata* Rothwell & Stockey; *Dorfiella knowltonii* (Dorf) Weber	Marsileaceae/Hydropteridaceae	–	–	Salviniaceae
Fern29	–	Gleicheniaceae	–	
Fern30 *Anemia elongata* (Newberry) Knowlton	–	–	Osmundaceae; Blechnaceae	Anemiaceae; Schizaeaceae
Fern31 *Diplazium crossii* Knowlton	–	–	Osmundaceae; Blechnaceae	Athyriaceae; Lonchitidaceae; Dryopteridaceae
Fern32	–	–	Osmundaceae	Pteridaceae
Fern33 *Equisetum* sp.	Equisetaceae	–	–	–

### Morphospace analysis

**Figure 4:**
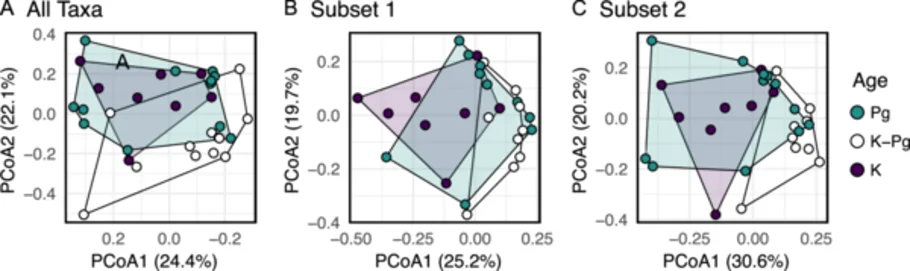
Morphospace occupation of fern morphotypes visualized using principal coordinate analysis (PCoA). (A) All Taxa refers to the most inclusive analysis, which consists of all morphotypes and all character states with at least one third of morphotypes scored. (B) Subset 1 excludes morphotypes with less than half of character states scored. (C) In Subset 2, character states that could not be scored for >60% of morphotypes were removed, followed by morphotypes with fewer than 60% of the remaining characters. Points are color‐coded based on the time interval in which the morphotype occurs; polygons indicate the morphospace occupied within each time interval. Percentage variation explained by each axis is given in parentheses in the axis label. The *x*‐axis of the All Taxa ordination was reflected to maintain consistent morphotype orientation across plots, as the sign of PCoA axis scores is arbitrary.

**Figure 5:**
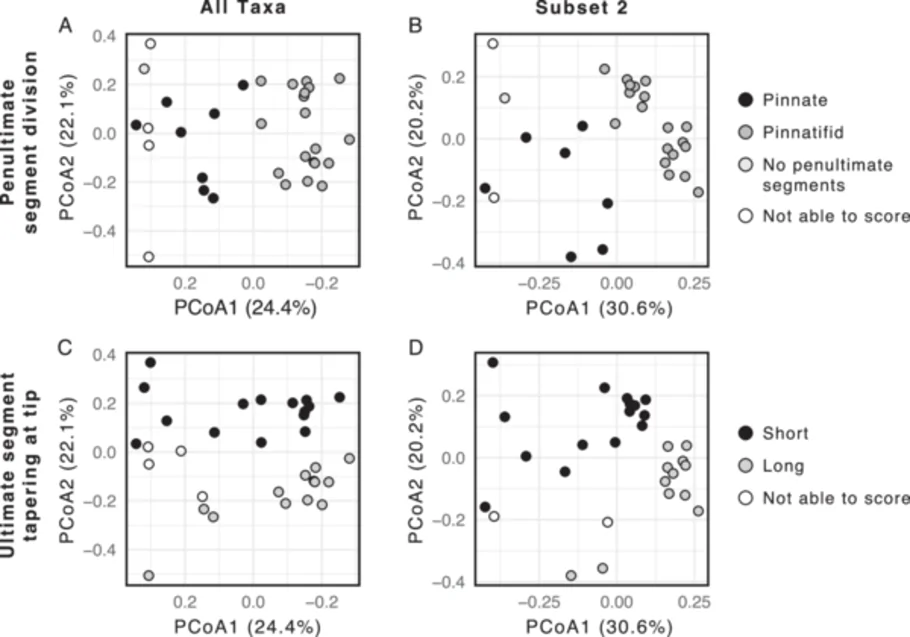
Principal coordinate analysis (PCoA) of fern morphotypes color‐coded by (A, B) penultimate segment division and (C, D) ultimate segment tapering at the tip. Morphotype ordination scores for the All Taxa data set (first column) are the same as in Figure 4A, and scores for the Subset 2 data set (second column) are the same as in Figure 4C. Penultimate segment division loads strongly onto Axis 1, with pinnate penultimate segments (black) on the left and pinnatifid penultimate segments (darker gray) on the right. Ultimate segment tapering at the tip loads strongly onto Axis 2, with short tapering (black) on top and long tapering (gray) on bottom.

Morphotypes plotted similarly in the PCoA regardless of which data set (All Taxa, Subset 1, or Subset 2) was used (Figure 4). The first axis explained between 24.4% and 30.6% of variation, and the second axis between 19.7% and 22.1%, depending on the data set. In all analyses, penultimate segment division loaded strongly on axis 1, and ultimate segment tapering at the tip loaded strongly on axis 2 (Figure 5).

Cretaceous and Pg morphotypes significantly overlapped in the occupied morphospace in all analyses, as did K–Pg and Pg morphotypes (Figure 4). The K and K–Pg morphotypes had less overlap, particularly in Subset 1 and Subset 2. Several characters likely played a key role in this pattern. Pinnatifid penultimate segment division was common in K–Pg (80%) and Pg (77%) taxa, whereas K taxa were more evenly divided between pinnate (50%) and pinnatifid (37.5%) penultimate segment division (the remaining K morphotype was simple and therefore does not have penultimate segments). Most K–Pg and Pg taxa had penultimate segments with long tapering at tip, whereas three of the four K morphotypes that could be scored for this character had short tapering at the tip. Penultimate segments were symmetrical for 80% of K–Pg and all Pg morphotypes, whereas K morphotypes were evenly split between symmetrical and asymmetrical penultimate segments. Margin type also varied across time intervals. All K taxa and 73% of Pg taxa were entire, whereas 55% of K–Pg taxa were serrate.

The amount of change in morphospace occupation (i.e., disparity) through time varied depending on the data set and quantification metric used (Table 3). In the All Taxa analysis, the hypercuboid volume and sum of ranges were similar for K, K–Pg, and Pg taxa. K–Pg taxa had lower average and higher maximum Gower pairwise distance than K or Pg taxa. However, these results should be treated with caution because three morphotypes in the All Taxa data set had character‐state completeness below 30%. Two of these taxa, Fern21 and Fern13, were K–Pg, and Fern21 plots far from any other morphotype in the PCoA (Figure 4A). When taxa with <50% character state completeness were removed (Subset 1), all four disparity metrics were lowest for K–Pg and highest for K taxa. Similarly, disparity was low for K–Pg taxa relative to K or Pg taxa when considering Subset 2. To examine full morphospace occupancy in the Cretaceous vs. the Paleogene, we grouped the K–Pg taxa with the K and the Pg taxa, respectively. The All Taxa and Subset 2 data sets showed little change between the full suite of Cretaceous taxa vs. the full suite of Paleogene taxa. In the Subset 1 data, all four disparity metrics had lower values for the Paleogene than for the Cretaceous.

**Table 3:** Morphological disparity metrics. Values for the four disparity metrics were determined for each data set (All Taxa, Subset 1, Subset 2) for taxa that only occur in the Cretaceous (K‐only), only occur in the Pg (Pg‐only), and occur in both the Cretaceous and Palgeoene (K–Pg), as well as for the full suite of taxa that occur in the Cretaceous (K‐only plus K–Pg, or K + K–Pg) and for the full suite of taxa that occur in the Paleogene (Pg‐only plus K–Pg, or Pg + K–Pg).

Dataset	Interval	No. of taxa	Hypercuboid volume	Sum of ranges PCoA 1‐4	Average gower pairwise distance	Maximum gower pairwise distance
**All taxa**	K‐only	8	0.052	1.92	0.50	0.78
	K–Pg	11	0.054	2.02	0.37	0.91
	Pg‐only	13	0.058	1.97	0.44	0.73
	K + K–Pg	19	0.10	2.32	0.46	0.92
	K–Pg + Pg	24	0.11	2.41	0.43	0.92
**Subset 1**	K‐only	8	0.087	2.18	0.50	0.78
	K–Pg	9	0.007	1.31	0.35	0.61
	Pg‐only	10	0.058	1.78	0.40	0.69
	K + K–Pg	17	0.13	2.39	0.46	0.85
	K–Pg + Pg	19	0.048	1.94	0.39	0.69
**Subset 2**	K‐only	8	0.056	1.97	0.46	0.75
	K–Pg	9	0.020	1.54	0.35	0.68
	Pg‐only	12	0.058	1.97	0.42	0.73
	K + K–Pg	17	0.095	2.25	0.43	0.85
	K–Pg + Pg	21	0.077	2.19	0.40	0.88

### Herbivory and pathogen damage results

**Figure 6:**
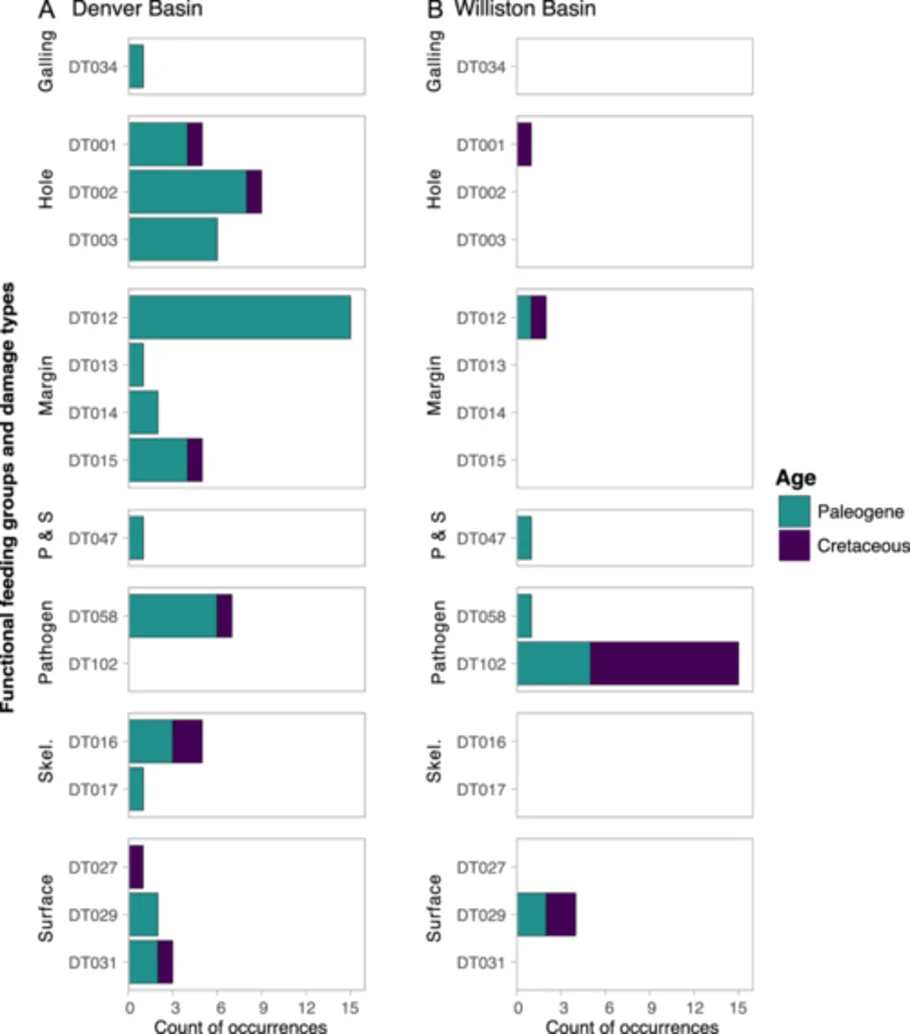
Occurrences of functional feeding groups and damage types (DT) in the Denver and Williston Basins color coded by age. No occurrences of damage were observed in the Raton Basin. In the Denver Basin, the five Cretaceous and 37 Paleogene fossil specimens exhibited herbivory/pathogen damage. For the Williston Basin, the count was 12 in the Cretaceous and 9 in the Paleogene. Note that multiple types of damage have been observed on individual fern specimens (see Appendix S1: Table S9). Functional feeding groups are abbreviated P & S = piercing and sucking; Skel. = skeletonization.

We observed 63 specimens with herbivory or pathogen damage, representing 8.02% of the data set. The specific DTs and functional feeding groups observed on each damaged specimen are given in Appendix S1 (Table S9). Margin feeding, hole feeding, surface feeding, skeletonization, and pathogen/environmental damage were observed in the Cretaceous specimens, and margin feeding, hole feeding, surface feeding, skeletonization, galling, piercing and sucking, and pathogen/environmental damage were observed in the Paleogene (Figure 6). The number of damaged specimens and DTs observed was greater in the Paleogene (*N* = 46 damaged specimens, 15 DTs) versus the Cretaceous (*N* = 17 damaged specimens, 10 DTs; Figure 6). Forty‐two of 390 specimens from the Denver Basin, 21 of 247 Williston Basin specimens, and none of the 141 Raton Basin specimens exhibited damage. Over 80% of the Raton Basin specimens came from Paleogene localities within 1 m of the K–Pg boundary, including 47 specimens from Starkville South localities that were ~25 cm above the boundary layer and 33 specimens from Clear Creek South localities that occur in the 50 cm above the K–Pg boundary.

**Figure 7:**
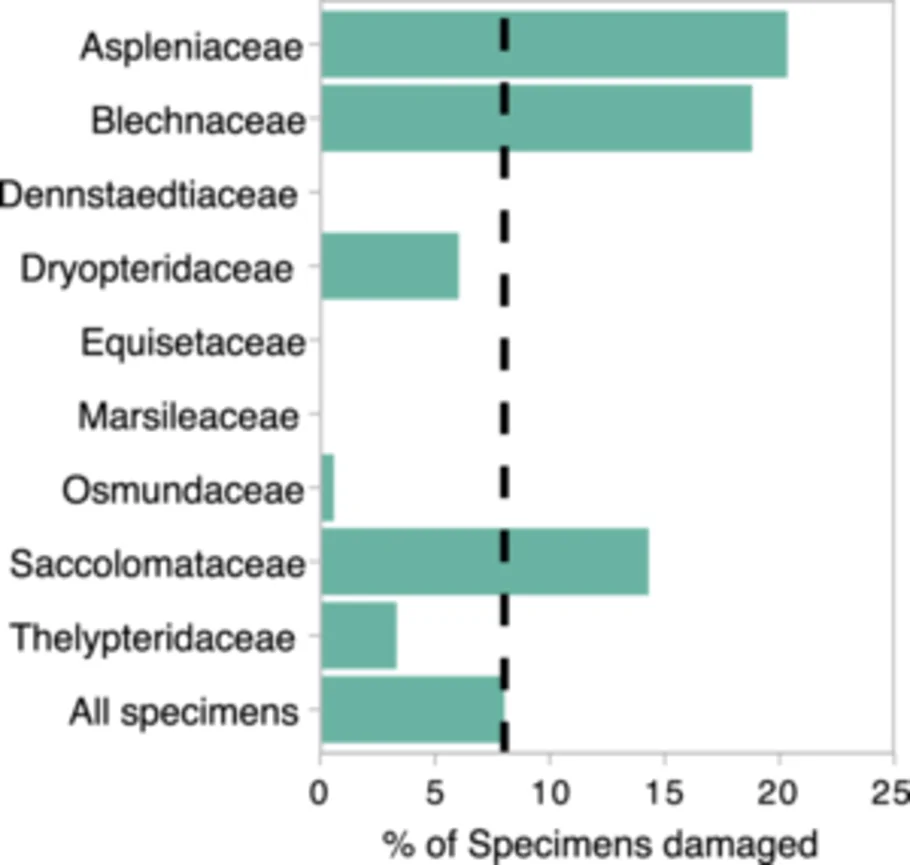
Percentage of fern specimens with herbivory or pathogen damage according to our best‐supported family hypotheses. Only families represented by at least 10 specimens are included. The black dashed line represents the overall damage rate across all specimens (8.02%, bottom bar), encompassing all specimens (*N* = 786) from this study. Families are represented by the following morphotypes with their total number of specimens examined: Aspleniaceae (Fern01; *N* = 123), Blechnaceae (Fern02, Fern03, Fern04; *N* = 133), Dennstaedtiaceae (Fern05, Fern06, Fern07; n = 52), Dryopteridaceae (Fern18, Fern20, Fern21, Fern22, Fern23; *N* = 116), Equisetaceae (Fern33; *N* = 23), Marsileaceae (Fern28; *N* = 14), Osmundaceae (Fern30, Fern31, Fern32; *N* = 167), Saccolomataceae (Fern12, Fern13; *N* = 14), and Thelypteridaceae (Fern08, Fern09, Fern10, Fern11; *N* = 30).

We also considered how damage frequency (% of leaves with damage) varied for fern families with >10 specimens, within the context of the morphotype best‐supported familial affiliation (Figure 7). We compared damage frequency for each family to the overall average rate (8.02%). Fern01 (*Allantodiopsis erosa* (Lesquereux) Knowlton et Maxon), belonging to Aspleniaceae, had a higher‐than‐average percentage of specimens with damage (20.33%), representing a diversity of damage types. Blechnaceae also had higher‐than‐average percentages of specimens with damage, with Fern02 at 15.11% and Fern03 either 9.52% or 28.53%, depending on whether the features we identified as DT102 are actually this DT or are instead irregular clasts within the larger sedimentary rock matrix. For Fern03, we are uncertain whether the observed structures indicate pathogen damage types or irregular clasts within the larger sedimentary rock matrix (Appendix S4, Plate 5B, DMNH.EPI.19968). Overall, Blechnaceae, including Fern05 (0% of specimens with damage), had a damage frequency of 12.78% or 18.80%.

Members of Dennstaedtiaceae (Fern05, Fern06, Fern07), Marsileaceae/Hydropteridaceae (Fern28), and Equisetaceae showed no evidence of herbivory. Within Thelypteridaceae (Fern08, Fern09, Fern10, Fern11), only one specimen (Fern10) exhibited damage, representing a damage frequency of 3.33%. In Osmundaceae (Fern30, Fern31, Fern32), damage was recorded only on a single specimen (Fern32), which was the only specimen of that morphotype, resulting in a 0.60% damage frequency for this family. Saccolomataceae had two damaged specimens, yielding a 14.29% damage frequency. Fern18 in Dryopteridaceae exhibited some specimens with damage (6.31%), with the family overall having a 6.03% damage frequency.

## DISCUSSION

In this study, we outlined a novel framework for describing fossilized fern fronds, analyzed possible fern familial affiliations to morphotypes around the Cretaceous–Paleogene boundary in Western North America, examined morphospace occupation, and recorded herbivory and pathogen damage on individual fossils. By adding insights to an understudied, but ecologically important group of plants, this work contributes to a deeper understanding of biodiversity dynamics during one of the most significant mass extinctions in Earth's history. Here, we address challenges of assigning familial affiliations, particularly in relation to frond dimorphism. We examine extinction and turnover dynamics, focusing on changes in familial occurrences and morphology across the K–Pg boundary. Additionally, we discuss how our macrofossil findings relate to varying interpretations in the literature about the fern spore spike. Then, we explore possible explanations for different frequencies of herbivory and pathogen damage. Finally, we present recommendations for nomenclature assignments and the application of familial hypotheses in databases.

### Taphonomic disparities between sterile and fertile fronds and their implications for fern morphological and familial interpretation

One of the primary challenges in assigning morphotypes of fossilized ferns to a specific family is that reproductive structures are often absent. This issue particularly affects species with frond dimorphism, wherein a single species produces morphologically distinct sterile and fertile fronds. In dimorphic species, reproductive structures may take the form of sori borne directly on an axis lacking leaf tissue, or sori may be located on highly modified fronds with reduced leaf tissue. Monomorphic species, in contrast, are species with sterile and fertile fronds that are morphologically identical.

Two potential major issues arise in assigning dimorphic ferns to a family. First, sterile and fertile fronds are often differentially preserved; fertile fronds of dimorphic species tend to be rarely preserved, based on our own observations. As such, fertile fronds are often short‐lived and deteriorate rapidly after the release of spores. Second, even when both sterile and fertile fronds of a dimorphic species are preserved, strong morphological differences between these fronds may lead to misidentification and inaccurate morphotype assignments.

**Figure 8:**
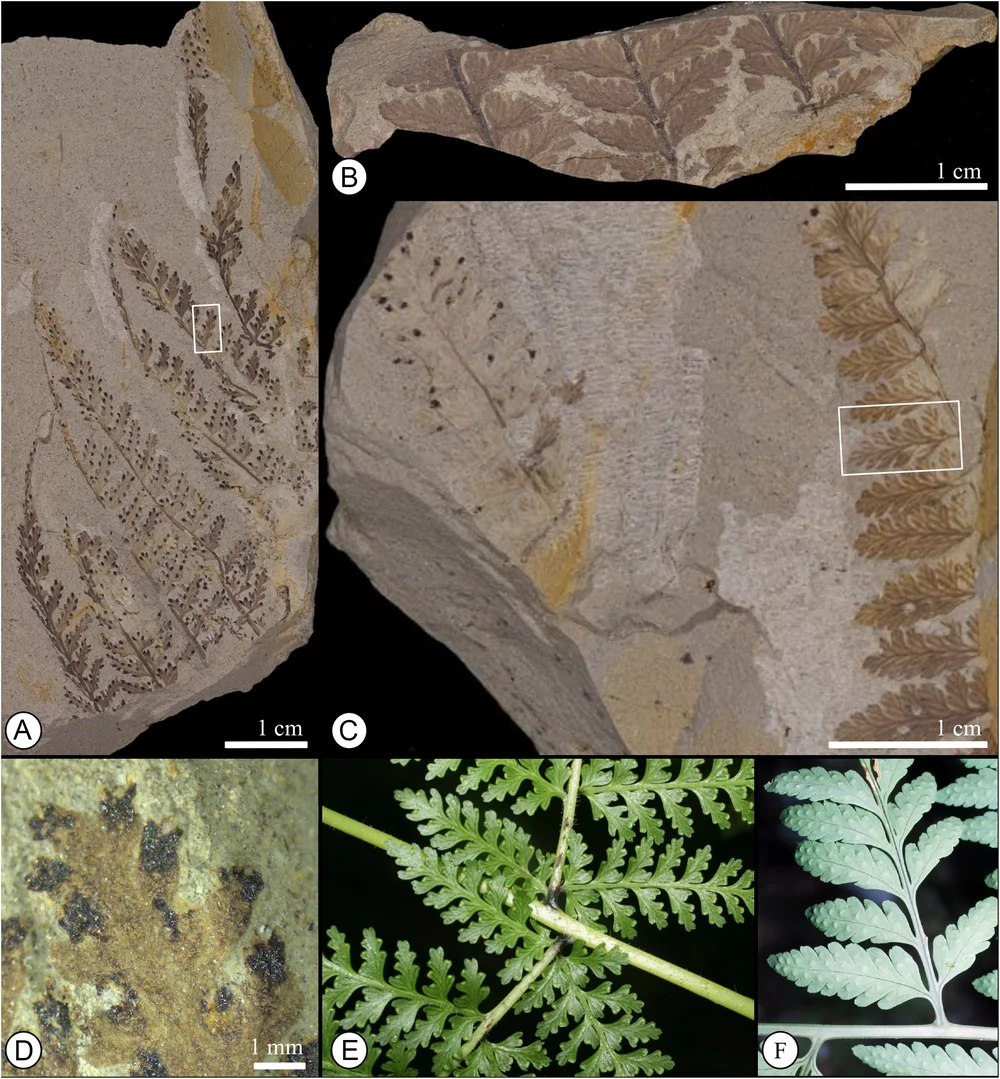
Fern05 *Dennstaedtia crossiana* Knowlton, Fern06, and modern comparisons. (A) Fertile quaternary ultimate segment (Fern05). Box outlines a penultimate segment, DMNH.EPI.52058. (B) Tertiary ultimate segments (Fern06), DMNH.EPI.52098. (C) Tertiary ultimate segments, both morphotypes on the same block: Fern05 (left), Fern06 (right). Box outlines penultimate segment, DMNH.EPI.52059. (D) Marginal sori with a likely cup‐shaped indusium (Fern05), DMNH.EPI.52074. (E) Dennstaedtiaceae, *Dennstaedtia cicutaria* (Sw.) T. Moore (Fern05, Fern06). (F) Dryopteridaceae, *Dryopteris patula* (Sw.) Underw. (Fern05). Modern fern images: Robbin Moran via plantsystematics.org.

For example, in our data set, what is likely a single fern species was split into two separate morphotypes (Fern05 and Fern06) because the sterile and fertile fronds appear distinct, despite strong co‐occurrence and some visual similarities (Figure 8). This distinction is notable because frond dimorphism is not found in the extant Dennstaedtiaceae, with which the fertile frond is strongly associated (Figure 8). To account for this discrepancy, we have maintained these morphotypes as separate, although we cannot definitively assert that they represent different species. (See Appendix S4, Fern05 and Fern06: Family assignment deliberations.)

**Figure 9:**
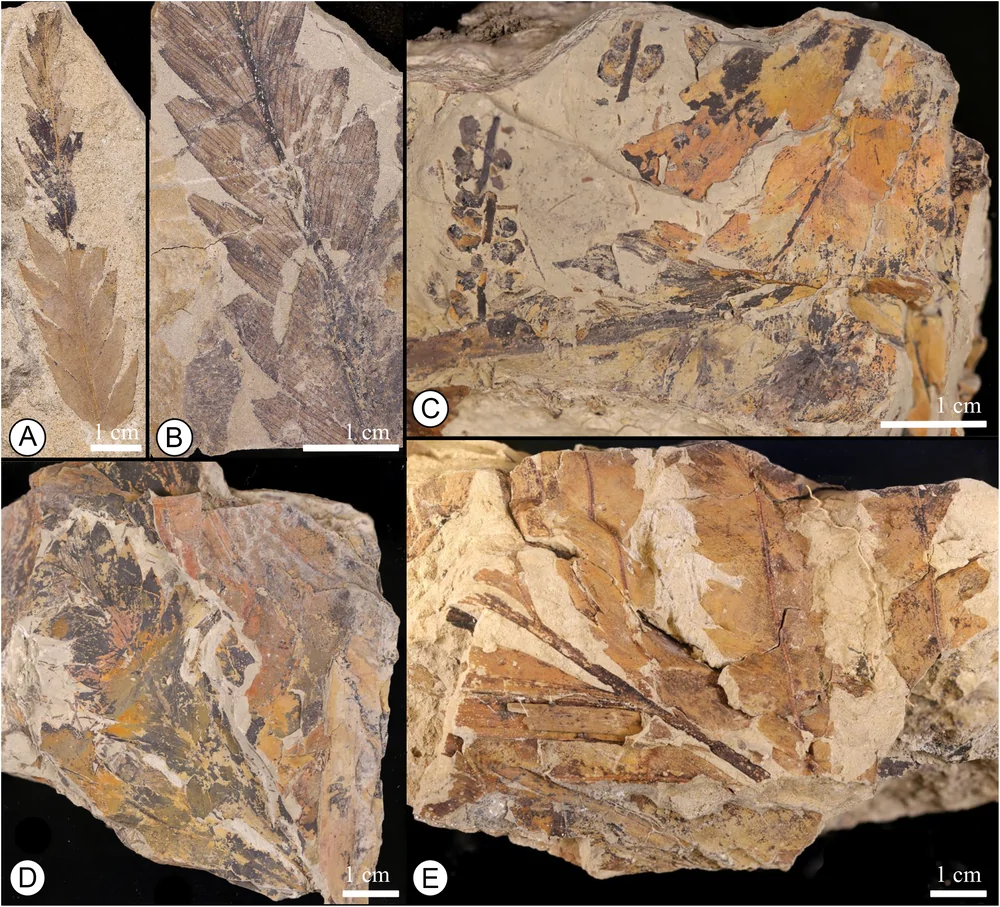
Fern31 “*Diplazium*” *crossii* Knowlton. (A) Penultimate segment, DMNH.EPI.51023. (B) Penultimate segment with near‐parallel venation free and forked and lacking an axial vein on ultimate segments, VPR.013. (C) Axis bearing sori? with possible association to Fern31, SS.025. (D) Penultimate segment long tapering at the base, SS.046. (E) Tertiary ultimate segment, CB.037.

Another pertinent example is Fern31, the most abundant morphotype in this study, represented by 156 specimens. Despite the abundance of Fern31 sterile fronds, fertile material has not been confidently associated with this morphotype. The only potential exception is one specimen (Figure 9C) that includes an axis likely bearing sori and a typical sterile Fern31 positioned adjacent to it. However, we cannot definitively confirm that these two structures belong to the same plant or morphotype. Regardless of the possible fertile association with Fern31, the combined abundance of this morphotype, along with the lack of reproductive structures observed, brings up an important question: Is this evidence of frond dimorphism? The lack of fertile structures found is not in itself evidence of frond dimorphism (for example, there are extant taxa, such as *Pteridium*, that are monomorphic but rarely fertile), but it is a reasonable working hypothesis for morphotypes that exhibit both high abundance and no observed reproductive structures.

A dimorphic hypothesis can serve as a useful starting point in narrowing down familial associations. Purely vegetative characters would need to be used for familial assignment. However, this approach results in, at best, moderate‐strength hypotheses due to the overlapping diversity of nonreproductive structure characters in fern families that have dimorphic fronds. Additionally, dimorphism is not exclusive to any particular clade of ferns; many families contain both monomorphic and dimorphic species. As a result, a dimorphic frond hypothesis may reduce the number of potential familial affiliations but does not offer definitive taxonomic resolution.

**Figure 10:**
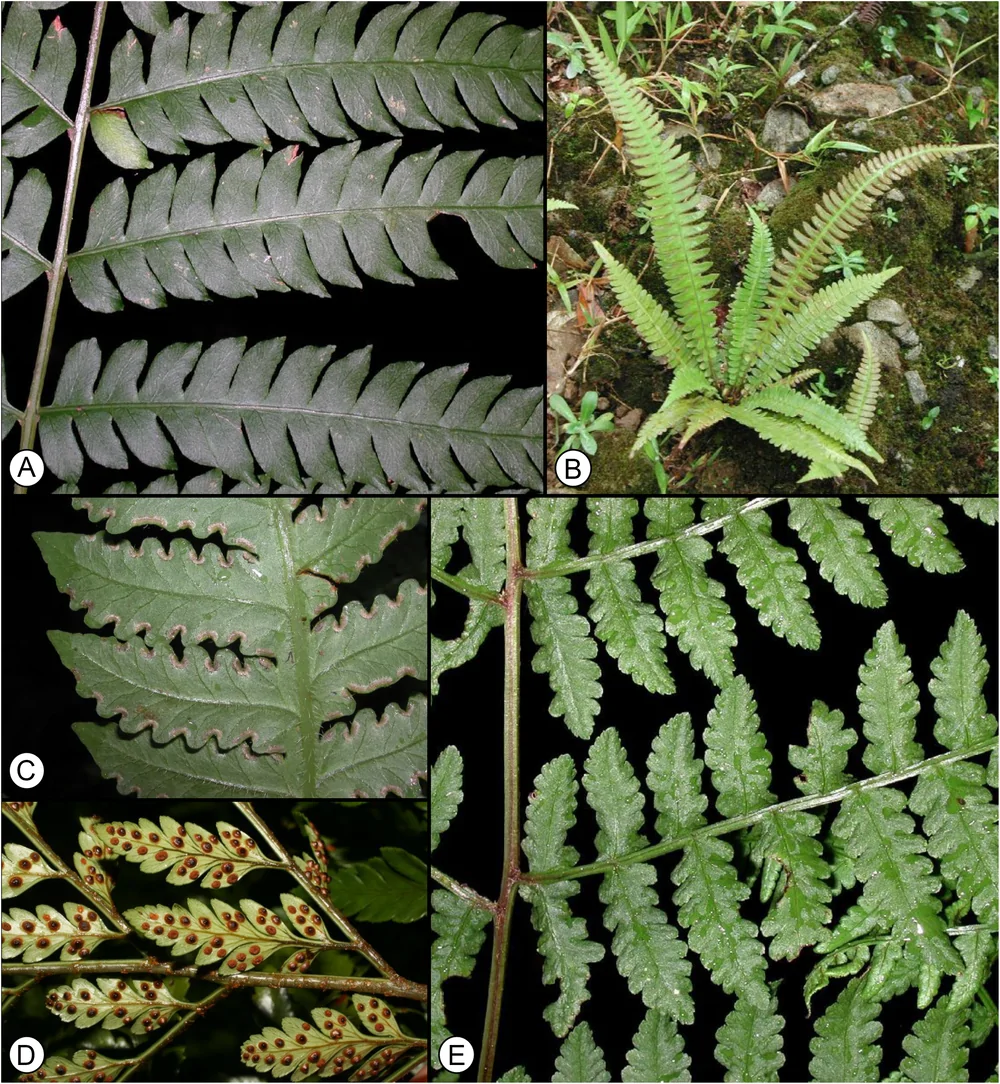
Fern31 “*Diplazium*” *crossii* modern comparisons. (A) Osmundaceae, *Osmunda cinnamomea* L. (B) Blechnaceae, *Blechnum polypodioides* (Sw.) Kuhn, (C) Lonchitidaceae, *Lonchitis hirsuta* L. (D) Dryopteridaceae, *Rumohra adiantiformis* (G. Forst.) Ching. (E) Athyriaceae, *Diplazium squamigerum* (Mett.) Matsum. Modern fern images: Robbin Moran via plantsystematics.org.

We suggest that family diagnoses involving strongly dimorphic fronds require a high threshold of evidence for strong and even moderate hypotheses. For example, Osmundaceae is the best‐supported hypothesis for Fern31 in our study (Figures 9, 10), but this hypothesis remains weak without associated dimorphic fertile structures.

### Extinction and turnover

Due to the limitations of fossil preservation both spatially and temporally, we cannot definitively attribute morphotype losses to the K–Pg mass extinction. Some morphotypes that occurred only in the Cretaceous may have gone extinct before the mass extinction. Others may not be present in the Paleogene of the basins we studied, but may have persisted in other areas around the world. Changes in depositional setting or preservation bias across the K–Pg boundary may also affect floral composition, although Wilson Deibel et al.'s (2024) analysis of census collections from 11 sites spanning the K–Pg boundary in the Williston Basin found no significant effect of depositional environment on floral composition. Our data also provide little support for depositional environment as a primary driver of temporal changes in fern composition. Of the 19 Cretaceous localities yielding identifiable fern specimens, four are channel deposits (21%), 12 are overbank deposits (63%), and three could not be assigned to a depositional environment (16%). Similarly, among 83 Paleogene localities, 13 are channel (16%), 51 are overbank (61%), and 19 are unknown (23%). Both depositional settings are represented in the Cretaceous and the Paleogene in the Denver and Williston basins; all localities from the Raton Basin are Paleogene, and all are overbank deposits.

In this study, we compared fossil morphotypes to extant fern families and chose not to establish new extinct families for morphotypes with uncertain affiliations. This approach allows for comparisons with living taxa but may obscure our interpretation of family‐level extinction at the K–Pg boundary because extant families cannot, by definition, have gone extinct. The resulting low extinction at higher fern taxonomic levels is consistent with Thompson and Ramírez‐Barahona (2023), who demonstrated that K–Pg extinction in angiosperms was more pronounced at the species level than at the family or order level.

**Figure 11:**
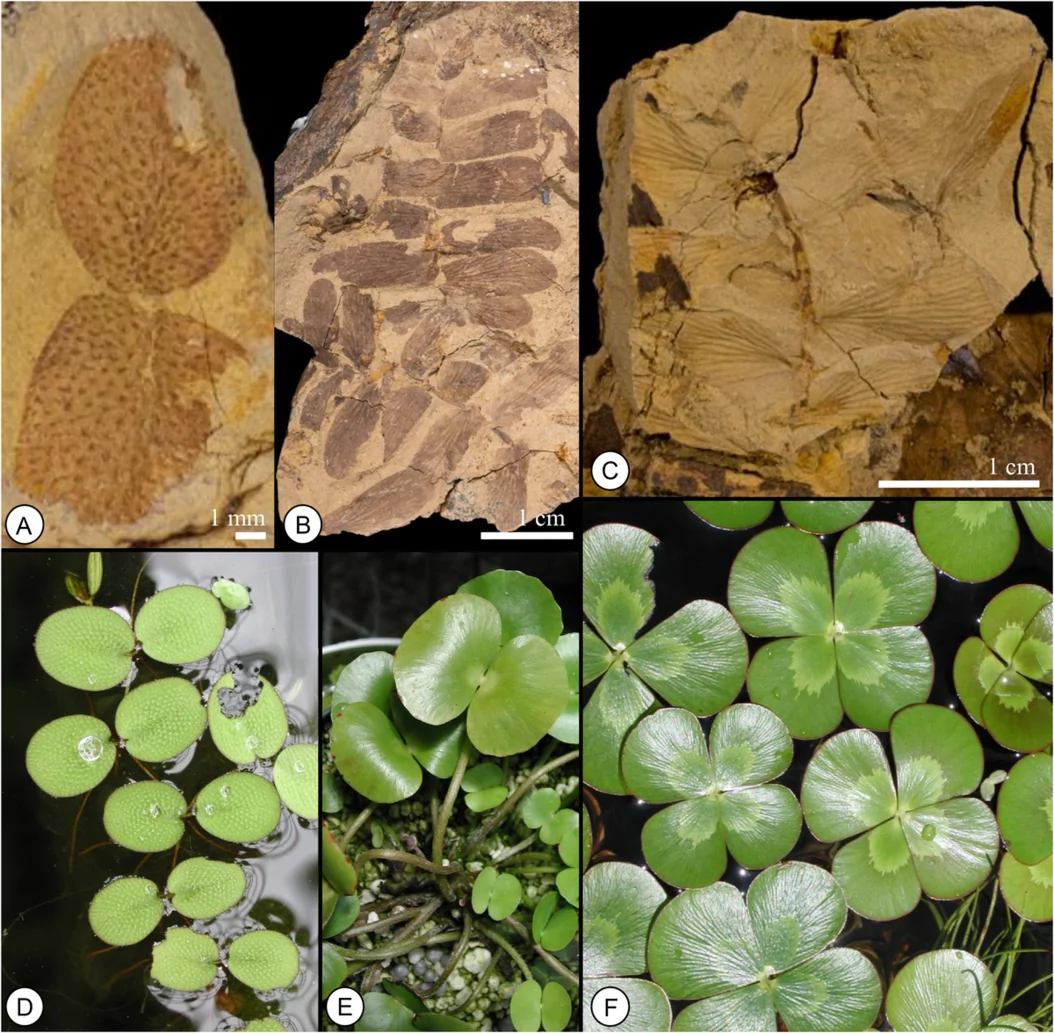
Fern27 *Salvinia* sp., Fern28 *Hydropteris pinnata* Rothwell & Stockey, and modern comparisons. (A) Paired blades/ultimate segments (Fern27), DMNH.EPI.23743; (B) penultimate segments (Fern28), DMNH.EPI.51240. (C) Penultimate segment (Fern28), DMNH.EPI.23744; (D) Salviniaceae, *Salvinia minima* Baker (Fern27, Fern28). (E) Marsileaceae, *Regnellidium diphyllum* Lindm. (Fern28). (F) Marsileaceae, *Marsilea mutica* Mett. (Fern28). Modern fern images: Robbin Moran via plantsystematics.org.

Only two of 11 families observed in the Cretaceous did not also occur in the Paleogene. These were Salviniaceae, represented by Fern27 (*Salvinia* sp.), and Marsileaceae represented by Fern28 (*Hydropteris pinnata* Rothwell & Stockey; Figure 11). They represent extant aquatic fern families in the order Salviniales (Pteridophyte Phylogeny Group, 2016; see Appendix S4, Fern28: Family assignment deliberations for discussion of alternative familial assignments). Fern27 is rare in our data set, represented by only two of the 168 Cretaceous specimens that could be assigned to a morphotype. It has nearly identical character states to modern *Salvinia* (see Appendix S4, Fern27: Family assignment deliberations), and fossil ferns identified as *Salvinia* are well known from the Paleocene and Eocene of western North America (Pigg and Devore, 2010). Salviniaceae is extant; however, members of the family may have faced extirpation, a shift in geographic distribution, and/or a brief decline. Additionally, it is possible that individual species of Salviniaceae may have gone extinct. On the other hand, *Hydropteris pinnata* has been reported from Late Cretaceous sites ranging from Utah, USA, to Alberta, Canada (Hermsen, 2025), and our data set includes 14 specimens from a total of eight localities in the Denver and Williston basins. If *Hydropteris* is placed into the extinct Hydropteridaceae rather than the extant Marsileaceae, it is possible that Hydropteridaceae went extinct. It is beyond the scope of this study, however, to determine whether this family went extinct due to the K–Pg mass extinction or to a background extinction earlier in the Cretaceous (Rothwell and Stockey, 1994; Pryer, 1999).

The only family that appears to have increased in diversity in the western interior of North America after the K–Pg boundary based on our data is Hymenophyllaceae, with three new morphotypes identified in the Paleogene. However, one of these morphotypes (Fern16) has a low MQI, a weak hypothesis strength, and is represented by only one specimen. Fern14 and Fern15 have medium MQIs and strong hypothesis strengths, but are represented by one and six specimens, respectively. Additionally, ferns in Hymenophyllaceae often have very delicate fronds that are one cell thick (Farrar, 2020), which reduces their capacity for preservation, potentially explaining why there are no specimens of this family from the Cretaceous. Conversely, there may have been more fossils in Hymenophyllaceae preserved in the Paleogene due to our larger sample size of this period.

### Morphological change across the K–Pg boundary

Our results indicate a contraction of occupied morphospace in taxa that crossed the K–Pg boundary, relative to taxa that occur only in the Cretaceous. This result is most pronounced if a data completeness threshold is set (i.e., the use of Subset 1 or Subset 2 dataset), but is also supported by the average Gower pairwise distance metric in the All Taxa analysis. Hypercuboid volume, sum of ranges of PCoA axes 1‐4, and maximum Gower pairwise distances are slightly higher for K–Pg taxa than for K taxa in the All Taxa analysis, but these metrics have high sensitivity to missing data (Ciampaglio et al., 2001). The values are likely inflated due to a single outlying taxon, Fern21, in which only 15% of characters could be scored. In contrast, the average Gower pairwise distance metric, which is more robust against missing data (Ciampaglio et al., 2001), decreases from 0.50 in K taxa to 0.37 in K–Pg taxa. Morphological characters that are more variable in K taxa than in K–Pg taxa include penultimate segment division, tertiary ultimate segment division, ultimate segment shape, and penultimate segment symmetry. The characters penultimate segment shape, vein type, and margin type are more variable in K–Pg than in K taxa.

Compared to morphotypes that only occur in the Cretaceous, those that cross the K–Pg boundary are more likely to have pinnatifid penultimate segment division, penultimate and ultimate segments that are long tapering at the tip, symmetrical penultimate segments, and serrate margins. Research on angiosperm leaves from the Williston Basin suggests that post‐impact conditions favored quick‐return taxa with low carbon investment and high carbon assimilation rate, as evidenced by low leaf mass per area and high vein density (Blonder et al., 2014; but note that Butrim et al., 2022, found no change in leaf mass per area across the K–Pg boundary in the Denver Basin). Pinnatifid segment division and long‐tapering apices may similarly allow for increased photosynthetic tissue at minimal carbon cost because leaf tissue is supported by the rachis or major venation, respectively. Within woody angiosperms, taxa with serrate margins are more abundant in cooler climates and disturbed habitats (e.g., Bailey and Sinnott, 1916). While margin type in ferns does not appear to correlate with climate, perhaps because modern ferns often grow in the understory and experience a different microclimate (Peppe et al., 2011), research on the functional significance of serrations in woody angiosperms may be relevant still. Baker‐Brosh and Peet (1997) documented increased photosynthetic activity in the serrations of temperate angiosperm trees, particularly early in the growing season, and Feild et al. (2005) proposed that leaf teeth in the herbaceous angiosperm *Chloranthus japonicus* facilitate release of guttation sap when roots absorb more water than the plant can transpire. It is important to note, though, that fern leaves are fundamentally different from angiosperm leaves in that they are the site of both photosynthesis and reproduction; additionally, they exhibit marginal growth, whereas angiosperm leaves grow via cell division throughout the leaf (Boyce, 2005). Linking fern morphological traits with environmental conditions is a promising avenue for future research. Ecological surveys could determine whether the characters highlighted here for K–Pg taxa are shared by extant taxa that colonize landscapes following volcanic eruptions, hurricanes, or other disturbances. Experiments could test whether these leaf characters correlate to physiological parameters such as photosynthesis, transpiration, respiration, or growth rates or to demographic variables such as fecundity or mortality.

Morphospace occupancy in Pg‐only taxa is similar to that of K‐only taxa (Table 3), and only the Subset 1 data provide evidence for a decrease in morphospace occupancy if boundary‐crossing taxa are added to Pg‐only and K‐only groups. Like the K–Pg taxa and in contrast to the K taxa, Pg taxa are more likely to have pinnatifid penultimate segment division, symmetrical penultimate segment division, and long tapering ultimate and penultimate segment tips. Additionally, most Pg taxa had penultimate segments with long tapering at the base, vs. predominantly short for both the K and K–Pg taxa. All Pg taxa have free and forked vein type.

### Macrofossils around the K–Pg boundary and the fern spore spike

*Cyathidites* and *Laevigatosporites* have been recognized as the most abundant fern spore types during the fern spore spike at the K–Pg boundary in western North America (Berry, 2023) and have also been observed worldwide, such as in New Zealand (Vajda et al., 2001). These spore types are likely not restricted to a single taxon and are considered “form genera” representing morphological similarity rather than a taxonomic grouping.

More traditional approaches have associated *Cyathidites* with “tree ferns” and *Laevigatosporites* with “ground ferns” (Vajda et al., 2001). Other interpretations have associated *Cyathidites* with the macrofossil *Dennastra sorimarginata* McIver and Basinger (represented by Fern12 and Fern13 in this study, but likely associated with Saccolomataceae, not Dennstaedtiaceae), and *Laevigatosporites* with *Woodwardia arctica* (Heer) RW Brown (Fern02; Berry, 2023). Similarly, *Anemia elongata* (Newberry) Knowlton (Fern30, Fern31) and *Allantodiopsis erosa* (Fern01) have been suggested as candidates for *Cyathidites* and *Laevigatosporites*, respectively (Berry, 2021).

Although we did not directly examine spores or associate them with macrofossils in this study, our macrofossil data surrounding the boundary may provide insight into the K–Pg fern spore spike. Most of the fossils examined in this study were not observed directly at the fern spore spike because this period was geologically brief, estimated to have occurred over the span of 1000–71,000 years (Clyde et al., 2016). The exception is for the sites at Clear Creek South (ECCC.2101, ECCC.2102, ECCC.2102 L, ECCC.2102U, ECCC.2103, ECCC.2103 L, ECCC.2103U, ECCC.2104) in the Raton Basin, where palynological samples were collected at the same time as macrofossils (A. Bercovici, Denver Museum of Nature and Science, personal communication). The only fern macrofossil morphotype we observed at Clear Creek South was Fern31, and the dominant fern spore was *Cyathidites*. We are uncertain of the familial affiliation of Fern31, but our results likely discredit the notion that the fern spore spike reflects a tree‐fern‐dominated landscape. This interpretation is bolstered by the fact that none of our sites had preservation of the hardy sclerenchyma tissue associated with tree fern “trunks”. The morphology of Fern31 does not reflect the morphology of any modern tree ferns, and none of our macrofossil morphotypes represent tree fern family associations in either the Cretaceous or Paleogene. Only one specimen in our data set (Fern19) had the best‐supported, but weak hypothesis of a tree fern in Cyatheaceae (Appendix S4: Plates 19 C and 19D, DMNH.EPI.51030). It is also plausible that Fern09 and Fern18 have misidentified best‐supported hypotheses, which could affirm a tree fern spore spike, because these morphotypes were both common and persist across the boundary. However, we find it unlikely that tree ferns were common in western North America in the early Paleogene, due to the lack of strong associations between our morphotypes and the various tree fern families.

In addition to Fern31, several morphotypes identified in this study may better correspond with *Cyathidites* in the fern spore spike rather than tree ferns, based on hypotheses from previous research (Berry, 2021). For example, Fern12 was found only in the Paleogene and Fern13 (representing *Dennastra sorimarginata*) persisted across the boundary. *Anemia elongata* has been suggested to be a plausible candidate of *Cyathidites* as well (Berry, 2021) and may be represented by both Fern30 and Fern31. In agreement with Berry (2021), we suggest that Fern12, Fern13, Fern30, and Fern31 may represent *Cyathidites* in the fern spore spike, with Fern31 directly collected with these palynological samples. For *Laevigatosporites*, Fern01 (*Allantodiopsis erosa*) and Fern02 (*Woodwardia arctica*) are abundant and span the boundary, supporting the possibility of this role in the fern spike. Further assessment of spores directly sampled from macrofossil sori and sporangia would help to better correspond macrofossil affiliations to the K–Pg fern spore spike.

Overall, we suggest that there is not a strong phylogenetic signal in the families/groups of ferns that are prevalent in the K–Pg fern spike records, which is consistent with modern fern spikes (Thomas and Cleal, 2022). Instead, the success of ferns during ecological upheavals may be driven by species‐specific adaptations that enabled particular species of ferns to survive challenging post‐extinction conditions as well as by pre‐existing geographical distributions before the disturbance.

### Herbivory and pathogen damage

The seemingly greater frequency and richness of damage types in the Paleogene is likely due to the larger sample size for the Paleogene (*N* = 540) compared to the Cretaceous (*N* = 197). The only two functional feeding groups observed in the Paleogene and not the Cretaceous, galling and piercing and sucking, had a total of one and two occurrences, respectively. Pathogen damage (DT102) seems to be abundant in the Cretaceous, but all occurrences are on the same morphotype (Fern03) at the same site (DMNH 2087; see Appendix S4, Plate 5 for image of the possible pathogen damage). Further, it is possible that these features are irregular clasts within the larger sedimentary rock matrix, rather than DT102. Because our study represents a survey of museum collections and did not follow standard practices for quantitative analyses of damage (Wilf and Labandeira, 1999), we did not pursue further analyses and caution against over‐interpretation of our results.

We suggest that the different attributes of the fern species influenced the occurrences of herbivory and pathogen damage we observed. Morphotypes with small segment sizes seemed to have fewer occurrences of damage than those with large segment sizes, which may be due to the lower nutritional value of smaller segments or to greater difficulty in recording herbivory on smaller segments. Marsiliaceae/Hydropteridaceae, along with Salviniaceae, may have had no occurrences of herbivory due to their water habit. Equisetaceae (Fern33) may also have had fewer occurrences due to the lower nutritional value of its branches. Many specimens in this family lacked leaves entirely, instead preserving other plant organs, such as axes, leaf sheaths, and tubers, making leaf damage unobservable. Leaf herbivory likely would also not be observed for Equisetaceae because feeding would be expected to be more frequent on stems and tubers, rather than the leaves.

Fern30 and Fern31 (both hypothesized to be Osmundaceae and potentially of the same species, but the actual affinity is uncertain, see Appendix S4, Fern30 and Fern31: Family assignment deliberations) had no occurrences of herbivory, despite their high abundance and large segments. This observation contrasts with our earlier hypothesis that larger segments would be associated with a greater occurrence of damage. Interestingly, most samples of Fern31 were found in Raton Basin sites within 1 m of the K–Pg boundary and, in many cases, from the same stratigraphic horizons as palynological samples of the fern spore spike. Because these samples are from shortly after the K–Pg extinction event, the absence of herbivory may reflect insect herbivore extinctions following the mass extinction. Alternatively, Fern30 and Fern31 could have had phytochemical defenses that deterred herbivores, allowing Fern31 to flourish during the period of post‐extinction ecological disruption. However, our ability to infer specific phytochemical defenses is limited by the uncertain taxonomic placement of these morphotypes. Osmundaceae, our best‐supported family hypothesis, have compounds such as phytoecdysteroids, flavonoids, and cyanogenic glycosides. However, this family is not unique in having chemical defenses; many other families have a wider diversity of compounds (Castrejón‐Varela et al., 2022). Ultimately, a more confident resolution of the taxonomic affiliation of Fern30 and Fern31 may allow for better understanding of potential phytochemical defenses based on the compounds in extant members of their lineage.

The observed pattern of low damage frequency and richness immediately after the K–Pg boundary is consistent with the findings of Labandeira et al. (2002), who studied damage on non‐monocotyledonous angiosperm leaves across the K–Pg boundary in the Williston Basin. They documented a ~42% decline in damage richness from the Cretaceous to the Paleogene, and floras collected within 1 m of the K–Pg boundary showed a ~25% less overall damage frequency and 75% lower frequency of DTs made by insects with restricted dietary breadth (i.e., intermediate or specialist feeders). Wilf et al. (2006) placed the results of Labandeira et al. (2002) in the context of Cretaceous to early Eocene floras from the western interior of the United States, including sites in the Williston and Denver basins, and found that herbivory remained depressed throughout the Paleocene, with the exception of a single site in Montana. Damage richness and frequency reported in these studies for non‐monocotyledonous angiosperms are significantly higher than our results for ferns, but we caution against direct comparisons given the very different sampling methods. New field collections that document damage richness and frequency in ferns around the K–Pg boundary represent a promising avenue for future research. In concert with studies of damage on other plant taxa, such work can document variations among basins and potentially disentangle whether spatial variations in damage richness in the Early Paleogene reflect insect survival in refugia or early diversification of Paleogene taxa (Donovan et al., 2014).

### Recommendations for nomenclature

We provide nomenclature recommendations for morphotypes with established names in the literature. Morphotypes without established names were not assigned recommendations. Recommendations and their rationale are summarized in Table 4. Appendix S4 provides more detailed explanations, including MQI, diagnostic characters, descriptions, other competing hypotheses, and family assignment deliberations.

**Table 4:** Nomenclature recommendations for fern fossil morphotypes examined in this study. Recommendations are based on our best‐supported family hypotheses for each morphotype, along with preferred “reference genera.” Morphotypes not included in this table have not been named in the literature.

Morphotype	Established name(s) in literature	Nomenclature recommendations and rationale
Fern01	*Allantodiopsis erosa*	No change recommended; *Allantodiopsis* is an extinct genus.
Fern02	*Onoclea hesperia; Woodwardia latiloba serrata; Woodwardia arctica*	Placement in Blechnaceae is correct. *Woodwardia* is inconsistent; *Blechnum* and *Salpichlaena* are better representations. *Onoclea* (Onocleaceae) is inconsistent.
Fern04	*Salpichlaena anceps*	No change recommended; morphotype is consistent with modern *Salpichlaena* (Blechnaceae).
Fern05	*Dennstaedtia crossiana*	No change recommended; morphotype is consistent with modern *Dennstaedtia* (Dennstaedtiaceae).
Fern12	*Saccoloma gardneri*	No change recommended; morphotype is consistent with modern *Saccoloma* (Saccolomataceae).
Fern18	*Dryopteris lakesii*	No change recommended; morphotype is consistent with modern *Dryopteris* (Dryopteridaceae).
Fern20	*Dryopteris integra*	No change recommended; morphotype is consistent with modern *Dryopteris* (Dryopteridaceae), but its low MQI warrants caution.
Fern27	*Salvinia* sp.	No change recommended; morphotype is consistent with modern *Salvinia* (Salviniaceae). Specific epithet not recommended due to unknown reproductive character states.
Fern28	*Hydropteris pinnata*	No change recommended; *Hydropteris* is an extinct genus.
Fern30	*Anemia elongata*	Name should not be retained. No strong evidence to suggest the morphotype is consistent with modern *Anemia* (Anemiaceae). No strong alternatives have been proposed in this study.
Fern31	*Diplazium crossii*	Name should not be retained. No strong evidence to suggest the morphotype is consistent with modern *Diplazium* (Athyriaceae). No strong alternatives have been proposed in this study.
Fern33	*Equisetum* sp.	No change recommended; morphotype is consistent with modern *Equisetum* (Equisetaceae). Specific epithet not recommended because the morphotype may represent multiple species of *Equisetum*.

### Recommendations for familial hypotheses in databases

Moving forward with our proposed best‐supported familial hypotheses, we recommend incorporating them into databases (e.g., the Integrative Paleobotany Portal (PBot) (Currano et al., 2021), Pteridophyte Portal (Rothfels et al., 2025), Paleobiology Database (Uhen et al., 2023)) and other resources according to their hypothesis strength. Strong hypotheses can be used in databases as accurate taxonomic placements because we are confident in these affiliations. Moderate hypotheses should be included in databases as suggestions, but should be clearly indicated as a possible taxonomic affiliation and should be investigated further. Weak hypotheses should not be included in databases to avoid propagating uncertain classifications, but should be investigated in future research. Additionally, higher taxonomic affiliations such as orders or suborders could be used in databases if competing hypotheses are all within a particular clade.

## CONCLUSIONS

In this study, we identified 698 fern macrofossils around the K–Pg boundary in western North America collected from the Raton, Denver, and Williston Basins. Specimens were organized into 33 distinct morphotypes. We proposed family affiliations for each morphotype and examined changes in morphology across the K–Pg using a newly developed schema for fossilized ferns. Additionally, we recorded evidence of herbivory and pathogen damage, finding that different families exhibited varying damage frequencies. This work contributes to our understanding of fern morphology, adaptation, and taxonomy in the context of one of Earth's most significant mass extinction events.

## AUTHOR CONTRIBUTIONS

F.B.H., E.D.C., E.B.S., R.E.D., J.L.G., and J.P. designed the research. F.B.H., R.S.B., R.E.D., K.R.J., S.A.M., I.M.M., and E.D.C. contributed to data collection for this project. F.B.H., E.D.C., and E.B.S. formulated the descriptive schema. F.B.H. delineated and described the fern morphotypes and conducted the family affiliations analyses, with input from E.D.C. and E.B.S. E.D.C. conducted the morphological analyses. All authors contributed to data interpretation. F.B.H. drafted the manuscript and made figures. All authors edited the manuscript.

## Supporting information


**Appendix S1:** Data tables of localities, specimens, morphotype character state summaries, matrices used for morphospace occupation analyses, and herbivory.

**Appendix S2:** Descriptive schema for fossilized ferns.

**Appendix S3:** R code for morphospace occupation analyses.

**Appendix S4:** Morphotype diagnostic characters, descriptions, family assignments, and plates.

## Data Availability

All data generated for this study can be found within the manuscript and the online supporting information.
