# Supplementary material for: Application of a novel schema for describing fossil fern foliage and investigating taxonomic and morphological change across the Cretaceous–Paleogene boundary in western North America
Source: Am J Bot. 2026 Jul 30;113(8):e70243. doi: 10.1002/ajb2.70243 (PMC13496116; doi:10.1002/ajb2.70243)
Supplement: Supplementary file 1 [file AJB2-113-e70243-s001.docx]

# **Appendix S4.** Morphotype diagnostic characters, descriptions, family assignments, and plates.

## ***Morphotype:*** Fern01

***Synonymous Morphotypes:*** CS1 (Lyson et al., 2019)

***Nomenclature to be Evaluated:*** *Allantodiopsis erosa* (Lesquereux) Knowlton et Maxon

***Age:*** Cretaceous and Paleocene

***Morphotype Quality Index:*** High

***Reference Specimens:*** DMNH.EPI.40382, DMNH.EPI.51033, DMNH.EPI.51320, DMNH.EPI.40385

***Number of Specimens:*** 123

***Observed Damage Types:*** Hole feeding DT001, DT002, DT003; margin feeding DT012, DT014, DT015; skeletonization DT016, DT017; surface feeding DT029, DT031; gall DT034; pathogen/environment DT058.

***Percent of Specimens with Herbivory/Pathogen Damage:*** 20.33%

***Study Sites:*** Denver Basin (Corral Bluffs, Plum Creek Parkway), Williston Basin, Raton Basin

***Diagnostic Characters:*** Ultimate segments elliptic, typically four times as long as wide. Venation free and forked, with each vein branching once or twice after departing the ultimate segment axial vein. Fronds are monomorphic, including fertile ultimate segments. Sori are elongated and centered between the axial vein of the ultimate segment and the margin, resting directly along veins.

***Description:*** Penultimate segments pinnate, *complete blade division not observed but likely pinnate,* ultimate segment shape elliptic, ultimate segment long tapering at the tip, ultimate segment short tapering at the base, ultimate segments symmetrical*, penultimate segment shape not observed,* penultimate segment long tapering at the tip*,* *penultimate segment tapering at the base not observed,* *penultimate segment symmetry not observed,* *complete frond shape not observed,* venation free and forked, veins terminate at the margin, *vein termination in lobe/segment and clefts between segments not applicable*, margin serrate, sori elongated, sori centered between the ultimate segment axial vein and margin on ultimate segments, true indusia elongate and originating on both sides of the sorus.

| ***Fern01 Family Assignment Hypotheses and Reference Genera*** | | |
| --- | --- | --- |
| **Hypothesis Strength** | **Family** | **Reference Genus** |
| Strong | Aspleniaceae | *Asplenium* |
| Weak | Athyriaceae | *Diplazium* |
| Weak | Diplaziopsidaceae | *Diplaziopsis; Hemidictyum* |
| Rejected | Thelypteridaceae | *Goniopteris* |
| Rejected | Osmundaceae | *Osmunda* |
| Rejected | Polypodiaceae | *Pleopeltis* |

***Family Assignment Deliberations:*** Multiple familial affinities of *Allantodiopsis erosa* (Lesquereux) Knowlton et Maxon have been proposed in the literature, resulting in an uncertain affinity (Manchester, 2014). Other studies have explored the resemblance of *A. erosa* to Athyriaceae or Aspleniaceae (Berry, 2021; Collinson, 2001; Knowlton, 1930; Lee and Knowlton, 1917; Lovis, 1977). Berry (2021) concluded that *A. erosa* is a *Diplaziopsis*-like asplenioid fern, implying similarity to another family, Diplaziopsidaceae. The elongated sori is the most notable character state on which to base a family assignment for *A. erosa*. The sori character states suggest that it is likely affiliated with one of the following families: Aspleniaceae, Athyriaceae, or Diplaziopsidaceae. These families all belong to the suborder Aspleniineae (eupolypods II) (Pteridophyte Phylogeny Group, 2016); therefore, we can confidently state that *A. erosa* belongs to Aspleniineae. Below, we assess the strengths of these family hypotheses and rejected hypotheses of other suggested taxa from our survey (Osmundaceae, Thelypteridaceae) based on morphological comparisons.

***Aspleniaceae:*** We propose the family Aspleniaceae as the strongest hypothesis for *A. erosa*, following Berry (2021). This classification is supported by the striking similarity between the sori of *A. erosa* and *Asplenium*, particularly the modern fern *Asplenium feei* Kunze ex Fée (Plate 2A). In both, the sori are elongated, appear to be embedded within the leaf tissue, and are directly borne on veins, all features that are characteristic of many modern species of *Asplenium*.

Although the indusia of *A. erosa* were poorly preserved, it can be reasonably inferred that its sori possess true indusia and possibly originate on both sides of the sorus, like in some members of *Asplenium.* Additionally, ferns in *Asplenium* are typically pinnate or are further divided with pinnate segmentation (Wagner et al., 2020), with *A. erosa* presumed to be at least pinnate. There are no strong morphological discrepancies between *A. erosa* and modern Aspleniaceae. Aspleniaceae is, therefore, concluded to be the strongest hypothesis for the classification of *Allantodiopsis erosa*.

***Athyriaceae:*** While it is plausible that *Allantodiopsis erosa* is affiliated with Athyriaceae due to its similarity to *Diplazium*, there are various inconsistencies that weaken this hypothesis. Modern *Diplazium* tend to exhibit elongated to linear sori, and in some cases, possess free forked venation and a pinnate structure (Plate 2B), which are all distinguishing features of *A. erosa* (Kato, 2020). However, modern *Diplazium* species exhibit a diverse range of sori morphologies, including single or paired sori, straight or slightly falcate in shape, and often display all of these character states within a single species. In contrast, *A. erosa* only has single, straight sori. Additionally, *Diplazium* can be pinnate like *A. erosa*, but the apex of the frond often transitions to a pinnatifid state, which is not observed in *A. erosa.* Athyriaceae cannot be completely ruled out, as character state differences with *A. erosa* are only exhibited in some species.

***Diplaziopsidaceae:*** Berry (2021) and Knowlton (1930) have suggested that *A. erosa* is a *Diplaziopsis*-like asplenioid fern. Modern Diplaziopsidaceae shares several characteristics with *A. erosa,* including elongated sori and a pinnate blade structure (Plate 2D). However, Diplaziopsidaceae often have anastomosing venation and elongated sori that are located along or near the axial veins of the ultimate segments (He and Kato, 2013). Conversely, *A. erosa* has sori centered between the ultimate segment axial veins and the margin, which is more characteristic of Aspleniaceae. This discrepancy indicates that Diplaziosidaceae is a weak hypothesis for *A. erosa*.

***Thelypteridaceae:*** Some species of *Goniopteris* (Thelypteridaceae) share some similar structures to *A. erosa,* such as ultimate segment shape, pinnate blade structure, and free venation, as seen in *Goniopteris falcatum* (Plate 2F). While species in Thelypteridaceae can have elongated or oblong sori, the sori rarely rest directly on veins (Smith, 2020b), like in *A. erosa*. Because there are wide inconsistencies between this family and *A. erosa*, this hypothesis can be rejected.

***Osmundaceae:*** The sterile ultimate segments of *A. erosa* morphologically resemble the sterile ultimate segments of some species of *Osmunda* (Osmundaceae), such as *Osmunda regalis* L. (Plate 2C), with no apparent morphological differences. However, a fundamental distinction between the two lies in the dimorphic fronds of Osmundaceae. The fertile fronds of Osmundaceae are reduced to sori borne on a midrib without the lamina (Zhang et al., 2013). Because *A. erosa* has monomorphic fronds where fertile fronds have sori borne on the leaf tissue, Osmundaceae is not a reasonable hypothesis and is rejected.

***Polypodiaceae:*** Ellis et al. (2003) suggested the hypothesis of Polypodiaceae for the affinity of *Allantodiopsis erosa*. However, Pelosi et al. (2022) calculated the stem age of Polypodiaceae to be 54.759 Ma based on molecular data, which is much younger than the K-Pg boundary to which the fossil is dated. Another estimate based on plastid data, however, has suggested the median age of Polypodiaceae to be 90.967 Ma (Testo and Sundue, 2016). Regardless of the age of the family, there are no specific modern genera or species in Polypodiaceae that have strong commonalities with *A. erosa*. *Pleopeltis astrolepis* (Liebm.) E.Fourn. (Plate 2E) exhibits elongated sori, but they are notably rounder than those in *A. erosa*. The sori are oriented parallel to the axial vein of the ultimate segment, rather than nearly perpendicular as in *A. erosa*. Based on the hypothesized stem age and morphology of Polypodiaceae, it seems unlikely that *Allantodiopsis erosa* belongs to this family, and this hypothesis can be rejected.

## ***Morphotype:*** Fern02

***Synonymous Morphotypes:*** CS7 (Lyson et al., 2019)

***Nomenclature to be Evaluated:*** *Onoclea hesperia* Brown, *Woodwardia latiloba serrata* Knowlton, & *Woodwardia arctica* (Heer) RW Brown

***Age of Rocks:*** Cretaceous and Paleocene

***Morphotype Quality Index:*** Medium

***Reference Specimens:*** DMNH.EPI.51254, DMNH.EPI.50996, DMNH.EPI.22660, UWBMPB.99706

***Number of Specimens:*** 86

***Observed Damage Types:*** Hole feeding DT001, DT002; margin feeding DT012; skeletonization DT016; surface DT029; piercing and sucking DT047?; pathogen/environment DT058, DT102.

***Percent of Specimens with Herbivory/Pathogen Damage:*** 15.11%

***Study Sites:*** Denver Basin (Corral Bluffs, Plum Creek Parkway), Williston Basin

***Diagnostic Characters:*** Tertiary ultimate segments bipinnatifid. Ultimate segments oblong to ovate. Venation anastomosing with areoles around the axial veins of penultimate and ultimate segments. Margin serrate.

***Description:*** Tertiary ultimate segments bipinnatifid, *complete blade structure not observed*, ultimate segment shape oblong to ovate, ultimate segment long tapering at the tip*, ultimate segment tapering at the base not applicable*, ultimate segments symmetrical, penultimate segment shape oblong to ovate, penultimate segment long tapering at the tip, *penultimate segment tapering at the base not applicable,* penultimate segments symmetrical, *tertiary ultimate segment shape not observed, tertiary ultimate segment tapering at the tip not observed, tertiary ultimate segment tapering at the base not observed, complete frond shape not observed,* venation anastomosing, veins terminate at the margin, vein termination in lobes/segments and clefts between segments, margin serrate, sporangia linear, sporangia along the sides of penultimate segment axial vein and (ultimate segment?) axial veins*, indusia not observed.*

| ***Fern02 Family Assignment Hypotheses and Reference Genera*** | | |
| --- | --- | --- |
| **Hypothesis Strength** | **Family** | **Reference Genus** |
| Strong | Blechnaceae | *Woodwardia* |
| Rejected | Onocleaceae | *Onoclea* |
| Rejected | Pteridaceae | *Pteris* |

***Family Assignment Deliberations:*** *Onoclea hesperia* Brown (Brown 1962), *Woodwardia latiloba serrata* Knowlton (Knowlton, 1930), and *Woodwardia arctica* (Heer) RW Brown (Brown, 1962) are names in the literature that match our identification of this morphotype. In our study, two specimens were identified to possibly represent the fertile foliage of Fern02 (DMNH.EPI.22660 (Plate 3C, Plate 3E); UWBMPB.99706 (Plate 3D)). Although both specimens are poorly preserved, all character states are consistent with the sterile foliage of this morphotype. They were found at two separate sites, which contained an abundance of sterile specimens of Fern02 (Das Goods, DMNH.2217; Biscuit Springs). We are confident that the fertile specimens belong to the same morphotype as the sterile ones, but have considered alternative hypotheses. ***Blechnaceae:*** The fertile foliage of this morphotype is most certainly affiliated with Blechnaceae. This is the only modern family of ferns that can exhibit linear sporangia positioned along both sides of an axial vein (Cranfill, 2020a). All descriptive character states of the sterile and fertile foliage of Fern02 are in congruence with the modern description of Blechnaceae as well. However, the use of the genus name *Woodwardia* (*Woodwardia latiloba serrata* Knowlton and *Woodwardia arctica* (Heer) RW Brown) is not a completely accurate depiction. Modern *Woodwardia* (Plate 4A) is often monomorphic and pinnatifid, with anastomosing venation forming distinctive areoles, similar to Fern02. However, they have discrete and usually elongated sori lined along both sides of an axial vein (Cranfill, 2020c). Fern02 does not have discrete sori, but instead linear sporangia. This feature is common in other genera of Blechnaceae, such as *Blechnum* (Plate 4B) or *Salpichlaena*. But, other character states of these genera are not consistent with Fern02, so we have not considered them to be preferred reference genera. Although *Woodwardia* is the modern genus most consistent with Fern02, its description is not completely consistent with this morphotype, making it an unsuitable name.

***Onocleaceae:*** *Onoclea hesperia* Brown is another name commonly used for this morphotype. The modern *Onoclea sensibilis* L. (Plate 4C) has a similar anastomosing venation pattern with distinctive areoles and a bipinnatifid structure, consistent with Fern02. *Onoclea* also has strongly dimorphic fronds with very reduced fertile fronds (Johnson, 2020). If the two fertile specimens are considered not to belong in the same morphotype as the sterile fronds, Onocleaceae would be a reasonable hypothesis, as the morphology of its sterile fronds is generally consistent with the fossils. However, with the inclusion of the fertile segments, Onocleaceae is a rejected hypothesis because it does not exhibit the characteristic linear sporangia.

***Pteridaceae:*** Similarly, when only considering the sterile foliage, Pteridaceae is an alternative hypothesis. Many modern species of Pteridaceae feature anastomosing venation and exhibit a wide diversity of blade structures, including bipinnatifid forms. Within Pteridaceae, *Pteris* (Plate 4D) is the closest resemblance to Fern02. However, *Pteris* and related genera tend to have monomorphic fronds (Nauman, 2020c), in which case we would likely find fertile foliage alongside sterile foliage and easily associate them as the same morphotype. But, there are no observed fronds with reproductive structures that resemble the sori or sporangia of *Pteris*, making this hypothesis less likely. Alternatively, if we include the fertile foliage as part of Fern02, then Pteridaceae as a hypothesis is completely discredited.

## ***Morphotype: Fern03***

***Synonymous Morphotypes:*** NA

***Nomenclature to be Evaluated:*** NA

***Age of Rocks:*** Cretaceous

***Morphotype Quality Index:*** Medium

***Reference Specimens:*** DMNH.EPI.21114, DMNH.EPI.21187

***Number of Specimens:*** 42

***Observed Damage Types:*** Hole feeding DT001?; margin feeding DT012; surface feeding DT029; pathogen/environment DT102.

***Percent of Specimens with Herbivory/Pathogen Damage:*** 28.57%

***Study Sites:*** Williston Basin

***Diagnostic Characters:*** Tertiary ultimate segment bipinnatifid. Venation anastomosing, axial vein is absent in ultimate segments.

***Description:*** Tertiary ultimate segments bipinnatifid, *complete blade structure not observed*, ultimate segment shape elliptic, ultimate segment short tapering at the tip, *ultimate segment tapering at the base not applicable,* ultimate segments symmetrical*,* penultimate segment shape elliptic, penultimate segment short tapering at the tip, *penultimate segment tapering at the base not applicable,* penultimate segments symmetrical, *tertiary ultimate segment shape not observed,* tertiary ultimate segment short tapering at the tip, *tertiary ultimate segment tapering at the base not observed, complete frond shape not observed,* venation anastomosing, veins terminate at the margin, vein termination in lobes/segments and clefts between segments, margin entire, *reproductive structures not observed.*

***Discussion:*** For this morphotype, we describe what might be interpreted by others as ‘lobes’ or ‘serrations’, instead as ultimate segments (see Plate 5A). This is for the sake of consistency and simplicity in our descriptions and applying our description schema (also see Appendix S2).

| ***Fern03 Family Assignment Hypotheses and Reference Genera*** | | |
| --- | --- | --- |
| **Hypothesis Strength** | **Family** | **Reference Genus** |
| Moderate | Blechnaceae | *Woodwardia* |
| Moderate | Onocleaceae | *Onoclea* |

***Family Assignment Deliberations:***

***Blechnaceae:*** Fern03 is similar to modern *Woodwardia* (Plate 5C) due to its bipinnatifid structure and anastomosing venation. Members of this family typically do not have lobes/slightly incised ultimate segments, as observed in Fern03. The leaf and segment shapes of Fern03 are not typical of Blechnaceae, but these are not character states that generally define this family. This hypothesis is moderate because no reproductive structures have been observed, and there are no clear inconsistencies between Fern03 and Blechnaceae descriptions.

***Onocleaceae:*** Onocleaceae is an alternative hypothesis to Blechnaceae and may be slightly weaker. Modern *Onoclea sensibilis* (Plate 5D) also exhibits anastomosing venation and a bipinnatifid structure. The ultimate segments are elliptic and can appear lobe-like. Additionally, there is a thin layer of leaf tissue surrounding the rachis and connecting penultimate segments, which is also in common with Fern03. This hypothesis adds the similarity in lobe/segment shape that is not observed in Blechnaceae. Reproductive structures would need to be preserved to confirm this moderate hypothesis.

## ***Morphotype: Fern04***

***Synonymous Morphotypes:*** CS15 (Lyson et al., 2019)

***Nomenclature to be Evaluated:*** *Salpichlaena anceps* (Lesquereux) Knowlton

***Age of Rocks:*** Paleocene

***Morphotype Quality Index:*** Medium

***Reference Specimens:*** DMNH.EPI.51248, DMNH.EPI.51182, DMNH.EPI.51184

***Number of Specimens:*** 5

***Observed Damage Types:*** None

***Percent of Specimens with Herbivory/Pathogen Damage:*** 0%

***Study Sites:*** Denver Basin (Corral Bluffs)

***Diagnostic Characters:*** Penultimate segments pinnate. Ultimate segments long and linear. Venation free and forked.

***Description:*** Penultimate segments pinnate, *complete blade structure not observed*, ultimate segment shape linear, ultimate segment short tapering at the tip, ultimate segment short tapering at the base, *ultimate segment symmetry not observed, penultimate segment shape not observed, penultimate segment tapering at the tip not observed, penultimate segment tapering at the base not observed,* *penultimate segment symmetry not observed,* *complete frond shape not observed,* venation free and forked, veins terminate at the margin, *vein termination in lobes/segments and clefts between segments not applicable,* margin crenate, sporangia linear, sporangia along both sides of the ultimate segment axial vein, *indusia not observed but likely true linear indusia.*

| ***Fern04 Family Assignment Hypotheses and Reference Genera*** | | |
| --- | --- | --- |
| **Hypothesis Strength** | **Family** | **Reference Genus** |
| Strong | Blechnaceae | *Salpichlaena; Parablechnum* |

***Family Assignment Deliberations:***

***Blechnaceae:*** The character states observed in Fern04 match those of *Salpichlaena anceps* (Knowlton) Lesquereux. Our collection includes a fertile specimen, whose sporangial shape and placement confirm its close relationship to modern Blechnaceae. Fern04 has long ultimate segments and free forked venation, like the modern species *Parablechnum cordatum* (Desv.) Gasper & Salino (Plate 6E) and *Salpichlaena papyrus* G.G.Cárdenas, Tuomisto & Lehtonen (Plate 6D). The fertile specimen closely resembles *S. papyrus* as well due to its linear sporangia surrounding the axial vein of the ultimate segment.

We are confident that the fertile and sterile specimens belong to the same morphotype because they were found at the same site (Matt’s Meeting, DMNH.3740) and they exhibit no distinguishing character states. Because the reproductive structure character states are diagnostic of Blechnaceae, we consider this classification as a strong hypothesis.

## ***Morphotype: Fern05***

***Synonymous Morphotypes:*** NA

***Nomenclature to be Evaluated:*** *Dennstaedtia crossiana* Knowlton

***Age of Rocks:*** Cretaceous

***Morphotype Quality Index:*** High

***Reference Specimens:*** DMNH.EPI.52058, DMNH.EPI.52059, DMNH.EPI.52074

***Number of Specimens:*** 15

***Observed Damage Types:*** None

***Percent of Specimens with Herbivory/Pathogen Damage:*** 0%

***Study Sites:*** Williston Basin

***Diagnostic Characters:*** Quaternary ultimate segments bipinnate-pinnatifid. Penultimate and ultimate segments asymmetrical across their axial veins. Small ultimate segments <2 mm; small penultimate segments <1 cm. Ultimate segments are asymmetrical in size across the axial vein of the penultimate segment. Venation free and simple. Sori round and on the margins, likely with cup-shaped indusia.

***Description:*** Quaternary ultimate segments bipinnate-pinnatifid, *complete blade structure not observed but likely bipinnate-pinnatifid*, ultimate segment shape oblong, ultimate segment short tapering at the tip, *ultimate segment tapering at the base not applicable,* ultimate segments symmetrical, penultimate segment shape oblong, penultimate segment short tapering at the tip, penultimate segment long tapering at the base, penultimate segments asymmetrical, tertiary ultimate segment shape lanceolate, tertiary ultimate segment long tapering at the tip, tertiary ultimate segment short tapering at the base, *quaternary ultimate segment shape not observed, quaternary ultimate segment tapering at the tip not observed, quaternary ultimate segment tapering at the base not observed, complete frond shape not observed*, venation free and forked, *vein termination at the margin not observed,* vein termination lobes/segments only, margin entire, sori round, sori discrete and not continuous on margin, *indusia not observed but likely true, cup-shaped indusia.*

| ***Fern05 Family Assignment Hypotheses and Reference Genera*** | | |
| --- | --- | --- |
| **Hypothesis Strength** | **Family** | **Reference Genus** |
| Strong | Dennstaedtiaceae | *Dennstaedtia* |

***Family Assignment Deliberations:***

***Dennstaedtiaceae:*** Fern05 matches *Dennstaedtia crossiana* Knowlton, and this published species undoubtedly belongs to Dennstaedtiaceae. Modern *Dennstaedtia* share key characteristics with the fossil in that they have sori on the margins that are round or cylindrical (Plate 7E). Additionally, their fronds are often highly divided and have free forked venation. (Nauman and Evans, 2020). *D. crossiana* appears nearly identical to modern species of *Dennstaedtia,* with no notable morphological differences.

## ***Morphotype: Fern06***

***Synonymous Morphotypes:*** NA

***Nomenclature to be Evaluated:*** NA

***Age of Rocks:*** Cretaceous

***Morphotype Quality Index:*** Medium

***Reference Specimens:*** DMNH.EPI.52098; DMNH.52059

***Number of Specimens:*** 33

***Observed Damage Types:*** None

***Percent of Specimens with Herbivory/Pathogen Damage:*** 0%

***Study Sites:*** Williston Basin

***Diagnostic Characters:*** Tertiary ultimate segments pinnate-pinnatifid. Penultimate and ultimate segments asymmetrical across their axial veins. Ultimate segment shapes ovate to elliptic. Venation free and forked without an axial vein on ultimate segments.

***Description:*** Tertiary ultimate segment pinnate-pinnatifid, *complete blade structure not observed*, ultimate segment shape ovate to elliptic, ultimate segment short tapering at the tip, *ultimate segment tapering at the base not applicable*, ultimate segments symmetrical, penultimate segment shape lanceolate to elliptic, penultimate segment short tapering at the tip, penultimate segment short tapering at the base, penultimate segments asymmetrical, *tertiary ultimate segment shape not observed but likely lanceolate,* tertiary ultimate segment long tapering at the tip, tertiary ultimate segment short tapering at the base, *complete frond shape not observed,* venation free and forked, veins terminate at the margin, vein termination in lobes/segments only, margin entire, *reproductive structures not observed.*

| ***Fern06 Family Assignment Hypotheses and Reference Genera*** | | |
| --- | --- | --- |
| **Hypothesis Strength** | **Family** | **Reference Genus** |
| Moderate | Dennstaedtiaceae | *Dennstaedtia* |
| Weak | Dryopteridaceae | *Dryopteris* |

***Family Assignment Deliberations:***

***Dennstaedtiaceae:*** A key challenge in interpreting Fern06 is determining the possible association with the fertile fronds of Fern05 (*Dennstaedtia crossiana*). The two morphotypes have strong similarities, and they are the only fern types to occur at the Petty Ferns site (DMNH.2798) in the Williston Basin, including even specimens on the same block (DMNH EPI.52059, Plate 7C). If these morphotypes actually represent one species, then this morphotype belongs to Dennstaedtiaceae, similar to modern *Dennstaedtia* (Plate 7E). Fern06 appears similar to modern Dennstaedtiaceae as well. It has free forked venation, at least a pinnate-pinnatifid structure, and no characteristics that could exclude it from this family.

 While Fern05 and Fern06 appear morphologically similar, they are not identical. In Fern06, the ultimate segments are more rounded. Fern05 has more narrow, and sometimes lobed, ultimate segments that support the sori. Because ferns in Dennstaedtiaceae have monomorphic fronds (Cranfill, 2020b), the association of the fertile and the sterile fronds cannot be fully resolved, and we treat Fern05 and Fern06 as separate morphotypes. The hypothesis of Dennstaedtiaceae for Fern06 remains moderate.

***Dryopteridaceae:*** Fern06 has a morphological appearance that is similar to modern *Dryopteris.* *Dryopteris patula* (Sw.) Underw. (Plate 7F) has lanceolate penultimate segments and elliptic ultimate segments, like Fern06. In addition, this species exhibits free forked venation and an entire margin. Dryopteridaceae is a weak hypothesis because the fertile foliage that resembles this family has not been observed or associated with Fern06, and we favor Dennstaedtiaceae due to the potential association with the fertile foliage of Fern05.

## ***Morphotype: Fern07***

***Synonymous Morphotypes:*** CS8 (Lyson et al., 2019)

***Nomenclature to be Evaluated:*** NA

***Age of Rocks:*** Paleocene

***Morphotype Quality Index:*** Medium

***Reference Specimens:*** DMNH.EPI.51189, DMNH.EPI.51191, DMNH.EPI.51242

***Number of Specimens:*** 4

***Observed Damage Types:*** None

***Percent of Specimens with Herbivory/Pathogen Damage:*** 0%

***Study Sites:*** Denver Basin (Corral Bluffs)

***Diagnostic Characters:*** Quaternary ultimate segments bipinnate-pinnatifid. Penultimate and ultimate segments symmetrical across their axial veins. Ultimate segment shapes rounded and ovate and typically 2-3 mm in height and 3-4 mm in width. Venation free and forked without an axial vein on ultimate segments.

***Description:*** Quaternary ultimate segment bipinnate-pinnatifid, *complete blade structure not observed*, ultimate segment shape ovate, ultimate segment short tapering at the tip, *ultimate segment tapering at the base not applicable*, ultimate segments symmetrical, penultimate segment shape elliptic, penultimate segment long tapering at the tip, penultimate segment short tapering at the base, penultimate segments symmetrical, *tertiary ultimate segment shape not observed,* *tertiary ultimate segment tapering at the tip not observed,* tertiary ultimate segment short tapering at the base, *quaternary ultimate segment shape not observed, quaternary ultimate segment tapering at the tip not observed, quaternary ultimate segment tapering at the base not observed, complete frond shape not observed,* venation free and forked, veins terminate at the margin, vein termination in lobes/segments only, margin entire, *reproductive structures not observed.*

| ***Fern07 Family Assignment Hypotheses and Reference Genera*** | | |
| --- | --- | --- |
| **Hypothesis Strength** | **Family** | **Reference Genus** |
| Weak | Dennstaedtiaceae | *Dennstaedtia* |
| Weak | Dryopteridaceae | *Dryopteris* |
| Weak | Anemiaceae | *Anemia* |
| Rejected | Davalliaceae | *Davallia* |

***Family Assignment Deliberations:***

***Dennstaedtiaceae:*** Many species in *Dennstaedtia* can exhibit an at least bipinnate-pinnatifid structure, as observed in Fern07. Their ultimate segments can be rounded, and the penultimate segments lanceolate to elliptic, as seen in *Dennstaedtia obtusifolia* (Willd.) T.Moore (Plate 8F). Additionally, this species has an entire margin and free forked venation. There are no characteristics that can distinguish this morphotype from *D. obtusifolia*; however, the absence of reproductive structures inhibits definitive confirmation. Dennstaedtiaceae is the best hypothesis for Fern07, but remains a weak hypothesis.

***Dryopteridaceae:*** Fern07 is similar to *Dryopteris marginalis* (L.) A.Gray (Plate 8C) in its ultimate and penultimate segment shapes and free forked venation. However, there is no axial vein in the ultimate segments of Fern07, like in *D. marginalis*. *Dryopteris* species commonly have a serrate margin or lobed, pointed segments. Fern014 has a strictly entire margin, which may differentiate it from Dryopteridaceae. Because this morphotype only has sterile segments and the margins are generally dissimilar, Dryopteridaceae as a hypothesis remains weak.

***Anemiaceae:*** Modern Anemiaceae species also have similar penultimate and ultimate segment shapes along with free forked venation, as observed in *Anemia irwinii* Mickel (Plate 8G). This species also exhibits a bipinnate-pinnatifid blade structure. If Fern07 belongs to Anemiaceae, it would need to have dimorphic fronds because this is a characteristic of the family, and could explain the lack of fertile fronds associated with it. However, with only four specimens identified, we have not strongly suggested that this species is dimorphic, and it is possible that fertile monomorphic fronds have not been found and identified yet. Ultimately, this is a weak hypothesis because the state of the reproductive structures has not been confirmed.

***Davalliaceae:*** The sterile ultimate and penultimate segments of *Davallia tyermanii* T.Moore (Plate 8E) have similar morphology to Fern07. However, no other members of this family bear a striking resemblance. Furthermore, the stem molecular age of Davalliaceae dates almost precisely at 66 Ma (Pelosi et al., 2022), whereas the site (Matt’s Meeting, DMNH.3740) where Fern07 dates to 65.463 Ma (Lyson et al., 2019). It is plausible that this family was established and resembled modern Davalliaceae during the Paleocene, but it is unlikely. Ultimately, due to the lack of striking resemblance to Davalliaceae, we reject this hypothesis.

## ***Morphotype: Fern08***

***Synonymous Morphotypes:*** CS3 (Lyson et al., 2019)

***Nomenclature to be Evaluated:*** NA

***Age of Rocks:*** Cretaceous and Paleocene

***Morphotype Quality Index:*** Medium

***Reference Specimens:*** DMNH.EPI.23742, DMNH.EPI.34378

***Number of Specimens:*** 4

***Observed Damage Types:*** None

***Percent of Specimens with Herbivory/Pathogen Damage:*** 0%

***Study Sites:*** Denver Basin (Corral Bluffs, Plum Creek Parkway), Williston Basin

***Diagnostic Characters:*** Tertiary ultimate segments pinnate-pinnatifid. Penultimate segments linear to elliptic, often overlapping, typically >7 cm in length. Ultimate segments symmetrical across the penultimate segment axial vein, with rounded tips, oblong, with a slight curved upturn, typically >1 cm in length. Venation free and simple.

***Description:*** Tertiary ultimate segment pinnate pinnatifid, *complete blade structure not observed*, ultimate segment shape oblong, ultimate segment short tapering at the tip, *ultimate segment tapering at the base not applicable*, ultimate segments symmetrical, penultimate segment shape linear to elliptic, penultimate segment long tapering at the tip, penultimate segment short tapering at the base, penultimate segments symmetrical, *tertiary ultimate segment shape not observed*, *tertiary ultimate segment tapering at the tip not observed, tertiary ultimate segment tapering at the base not observed,* *complete frond shape not observed,* venation free and simple, veins terminate at the margin, vein termination in lobes/segments only, margin entire, *reproductive structures not observed.*

| ***Fern08 Family Assignment Hypotheses and Reference Genera*** | | |
| --- | --- | --- |
| **Hypothesis Strength** | **Family** | **Reference Genus** |
| Strong | Thelypteridaceae | *Goniopteris* |
| Weak | Osmundaceae | *Osmunda* |
| Weak | Athyriaceae | *Diplazium* |

***Family Assignment Deliberations:*** The most prominent uncertainty in assigning Fern08 to a family is the unknown appearance of its fertile fronds. Fern09, represented by only one fragmentary specimen, may represent part of a fertile frond of Fern08; yet, we retain these as separate morphotypes because of slight differences in ultimate segment shape. Thelypteridaceae tends to be monomorphic or can be slightly dimorphic (Smith, 2020b), so it is reasonable to suggest that these two morphotypes may represent a single morphotype instead. If Fern08 and Fern09 are considered to be the same morphotype, this further strengthens the hypothesis of Thelypteridaceae for Fern08 (see Fern09 Family Assignment Deliberations).

***Thelypteridaceae:*** The strongest hypothesis for this morphotype is Thelypteridaceae due to the similarity to modern *Goniopteris* with the nearly identical appearance to *Goniopteris curta* (Christ) [A.R.Sm.](http://a.r.sm/) (Plate 9C). Both have free, simple venation that only terminates within the ultimate segments of the penultimate segments. Also in common, the penultimate segments vary in shape, with linear-shaped segments with near parallel margins and elliptic segments that widen toward the center. Additionally, the penultimate segments appear to be slightly overlapping. Ultimate segments are oblong with a slight curved upturn. Finally, they both possess at least a pinnate-pinnatifid tertiary ultimate segment structure. There are no characteristics of Thelypteridaceae (*Goniopteris*) that are inconsistent with Fern08. The plausibility that Fern09 contains the fertile frond of Fern08 supports this hypothesis as well.

***Osmundaceae:*** Osmundaceae, specifically *Osmunda*, is a weak hypothesis for Fern08. This morphotype has notable similarities to modern *Osmunda claytoniana* (Plate 9E). The penultimate segment shapes of *O. claytoniana* are linear, and the ultimate segment shapes are oblong and slightly falcate. This species additionally has a pinnate-pinnatifid tertiary ultimate segment structure. However, Osmundaceae typically is restricted to free forked venation (Zhang et al., 2013), whereas Fern08 has exclusively free simple venation. Although it is reasonable that Fern003 fits into Osmundaceae, the difference in venation makes this family an unlikely hypothesis. Finally, if Fern08 belongs to Osmundaceae, this rules out the hypothesis of Fern005 exhibiting the fertile frond of this morphotype due to the strongly dimorphic nature of Osmundaceae.

***Athyriaceae:*** There are no strong differences between the reference genus *Diplazium* and Fern08. Species in *Diplazium* can have a pinnate-pinnatifid tertiary ultimate segment structure, oblong ultimate segment shapes that are slightly falcate, linear penultimate segment shapes, and simple venation (Kato, 2020). However, no modern species of Athyriaceae (*Diplazium*) have a striking resemblance to Fern08, as observed in Thelypteridaceae. The closest modern match to Fern08 we have identified is *Diplazium striatastrum* Lellinger (Plate 9D); however, the clefts of the ultimate segments are not as deeply incised as in Fern08, the margin is serrate, and the penultimate segments have much longer tapering. This hypothesis cannot be completely ruled out, but it remains weak because the similarities are not overwhelmingly consistent with any one modern species.

## ***Morphotype: Fern09***

***Synonymous Morphotypes:*** NA

***Nomenclature to be Evaluated:*** NA

***Age of Rocks:*** Cretaceous and Paleocene

***Morphotype Quality Index:*** Medium

***Reference Specimens:*** DMNH.EPI.58775

***Number of Specimens:*** 19

***Observed Damage Types:*** None

***Percent of Specimens with Herbivory Damage:*** 0%

***Study Sites:*** Denver Basin (Corral Bluffs)

***Diagnostic Characters:*** Penultimate segment pinnatifid. Venation free and simple. Ultimate segments bear small, round sori centered between the axial vein of the ultimate segment and the margin, resting directly on veins.

***Description:*** Pinnatifid penultimate segment, *complete blade structure not observed*, ultimate segment shape oblong, ultimate segment long tapering at the tip, *ultimate segment tapering at the base not applicable*, ultimate segments symmetrical, *penultimate segment shape not observed*, penultimate segment long tapering at the tip, *penultimate segment tapering at the base not observed*, *penultimate segment symmetry not observed, complete frond shape not observed,* venation free and simple, veins terminate at the margin, vein termination in lobes/segments only, margin entire, sori round, sori centered between ultimate segment axial vein and margin, *indusia not observed*.

| ***Fern08 Family Assignment Hypotheses and Reference Genera*** | | |
| --- | --- | --- |
| **Hypothesis Strength** | **Family** | **Reference Genus** |
| Strong | Thelypteridaceae | *Goniopteris* |
| Weak | Cyatheaceae | *Cyathea* |

***Family Assignment Deliberations:***

***Thelypteridaceae:*** Fern09 appears most similar to the modern species *Goniopteris curta* (Plate 10C) in Thelypteridaceae. They both have pinnatifid penultimate segments, simple venation, and small, round sori, centered between the ultimate segment axial vein and the margin, that rest directly on veins. This includes the oblong ultimate segments with the slight falcate upturn. There are no strong inconsistencies between Thelypteridaceae and Fern09, suggesting this family is a strong hypothesis.

We propose Fern08 and Fern09 belong in Thelypteridaceae and are considered *Goniopteris*-like (also see Fern08 Family Assignment Deliberations). The lack of observed differences in descriptions and character states between these two groups, suggests that there is a moderately strong indication they could combine to the same morphotype. But, due to slight differences in the ultimate segment shapes between the two, Fern08 and Fern09 have been kept as separate morphotypes.

***Cyatheaceae:*** An alternative family proposed for this morphotype is Cyatheaceae, with a resemblance to *Cyathea*. The most similar in morphology is *Cyathea bipinnatifida* Copel. (Plate 10D). Like Fern09, this species has small, round sori. Additionally, it has a pinnate-pinnatifid blade structure, which is a plausible higher segment division of Fern005. Yet, there is a range of morphological diversity in Cyatheaceae. Many species have a serrate to crenate margin or are lobed. Sori are often positioned directly along both sides of the axial vein of the ultimate segment, but can be centered between the ultimate segment axial vein and margin. Finally, there is a wide range of sorus size in Cyatheaceae; *C. bipinnatifida* representing the smaller end of this spectrum. There are no characters that can specifically rule out Cyatheaceae as a hypothesis, but there are no modern species that overwhelmingly resemble Fern09, unlike in Thelypteridaceae.

## ***Morphotype: Fern10***

***Synonymous Morphotypes:*** NA

***Nomenclature to be Evaluated:*** NA

***Age of Rocks:*** Paleocene

***Morphotype Quality Index:*** Medium

***Reference Specimens:*** DMNH.EPI.40369, DMNH.EPI.40370

***Number of Specimens:*** 5

***Observed Damage Types:*** Margin feeding DT012; piercing and sucking DT047; pathogen/environment DT058.

***Percent of Specimens with Herbivory/Pathogen Damage:*** 20%

***Study Sites:*** Denver Basin (Corral Bluffs, West Bijou)

***Diagnostic Characters:*** Penultimate segment pinnatifid. Penultimate segment shape lanceolate (approx. 7 cm in length and 1-2 cm in width). Venation free and forked. Ultimate segments bear small, round sori centered between the axial vein of the ultimate segment and the margin, resting directly on veins.

***Description:*** Penultimate segment pinnatifid, *complete blade structure not observed*, ultimate segment shape oblong, ultimate segment long tapering at the tip, *ultimate segment tapering at the base not applicable*, ultimate segments symmetrical, penultimate segment shape lanceolate, penultimate segment long tapering at the tip, penultimate segment short tapering at the base, *penultimate segment symmetry not observed, complete frond shape not observed,* venation free and forked, veins terminate at the margin, vein termination in lobes/segments only, margin entire, sori round, sori centered between ultimate segment axial vein and margin of ultimate segments, *indusia not observed***.**

| ***Fern10 Family Assignment Hypotheses and Reference Genera*** | | |
| --- | --- | --- |
| **Hypothesis Strength** | **Family** | **Reference Genus** |
| Strong | Thelypteridaceae | *Goniopteris* |
| Moderate | Dryopteridaceae | *Dryopteris* |
| Rejected | Nephrolepidaceae | *Nephrolepis* |

***Family Assignment Deliberations:***

***Thelypteridaceae:*** Fern10 has similarities to the family Thelypteridaceae, particularly the modern species, *Goniopteris curta* (Plate 11C)*.* In congruence with *G. curta*, Fern10 has small round sori centered between the axial vein of the ultimate segment and the margin, oblong, slightly falcate ultimate segments, and at least a pinnatifid penultimate segment. *G. curta* does not have free forked venation as observed in Fern10, but this is a character state observed in other members of the Thelypteridaceae, such as *Thelypteris* (Plate 11D). No character states distinguish Thelypteridaceae from Fern10, making this family a strong hypothesis.

***Dryopteridaceae:*** A weaker alternative hypothesis for Fern10 is Dryopteridaceae, with the similarity to the genus *Dryopteris.* Some modern species of *Dryopteris* have a similar pinnatifid penultimate segment structure and round sori, as seen in *Dryopteris goldiana* Dowell (Plate 11E). However, the sori of modern *Dryopteris* tend to not be generally placed directly along veins and have a diversity of sizes. *Dryopteris* often has a serrate margin, lobes, or is highly divided into small segments with these characters grading into each other. This contrasts with the smooth, entire margin of Fern10. These minor differences make Dryopteridaceae reasonable but not the best-supported hypothesis.

***Nephrolepidaceae:*** Nephrolepidaceae was another suggested family in the survey we conducted. In common with Fern10, members of the genus *Nephrolepis* (Plate 11F) often have round sori and free forked venation. Ferns in *Nephrolepis* are typically pinnate but can have a minutely pinnatifid attachment of ultimate segments to the penultimate segment (Nauman, 2020b), similar to Fern10. Despite this, the ultimate segments of *Nephrolepis* tend to have a distinctly asymmetrical shape, with an uncentered axial vein. These discrepancies lead us to reject the hypothesis of Nephrolepidaceae for Fern10.

## ***Morphotype: Fern11***

***Synonymous Morphotypes:*** NA

***Nomenclature to be Evaluated:*** NA

***Age of Rocks:*** Paleocene

***Morphotype Quality Index:*** Low

***Reference Specimens:*** DMNH.EPI.40376, DMNH.EPI.51024

***Number of Specimens:*** 2

***Observed Damage Types:*** None

***Percent of Specimens with Herbivory/Pathogen Damage:*** 0%

***Study Sites:*** Denver Basin (Corral Bluffs)

***Diagnostic Characters:*** Tertiary ultimate segments pinnate-pinnatifid. Penultimate segments very straight and linear, typically >11 cm in length. Ultimate segments very straight and oblong with rounded tips, typically 1-2 cm in length.

***Description:*** Tertiary ultimate segment pinnate-pinnatifid, *complete blade structure not observed*, ultimate segment shape oblong, ultimate segment short tapering at the tip, *ultimate segment tapering at the base not applicable*, ultimate segments symmetrical, penultimate segment shape linear, penultimate segment long tapering at the tip, penultimate segment short tapering at the base, penultimate segments symmetrical, *tertiary ultimate segment shape not observed*, *tertiary ultimate segment tapering at the tip not observed, tertiary ultimate segment tapering at the base not observed, complete frond shape not observed, venation not observed*, margin entire, *reproductive structures not observed.*

***Discussion:*** Fern11 was originally placed in the same morphotype as Fern08, but upon further inspection, there are notable differences in segment morphology. The ultimate segments of Fern11 are very straight and oblong, as opposed to the falcate and oblong ultimate segments in Fern08. Additionally, the penultimate segments of Fern11 are likely much longer, being >11 cm in length compared to >7 cm in length of Fern08. Therefore, because most other characters are consistent with Fern08, it is hypothesized that these two morphotypes are closely related but distinct.

| ***Fern11 Family Assignment Hypotheses and Reference Genera*** | | |
| --- | --- | --- |
| **Hypothesis Strength** | **Family** | **Reference Genus** |
| Weak | Thelypteridaceae | *Steiropteris* |
| Weak | Gleicheniaceae | *–* |
| Rejected | Athyriaceae | *Diplazium* |
| Rejected | Osmundaceae | *Osmunda* |

***Family Assignment Deliberations:***

***Thelypteridaceae:*** The best, albeit weak, hypothesis for Fern11 is Thelypteridaceae as it resembles the modern genus *Steiropteris*. *Steiropteris decussata* (L.) A.R.Sm. (Plate 12C) comparably has the straight, oblong ultimate segments seen in Fern11. Both appear to have at least pinnate-pinnatifid tertiary ultimate segments, and it is possible that Fern11 has free simple venation, although the preservation is poor. There are no notable differences between the character states of Thelypteridaceae and Fern11, but because the lack of well-preserved fossils and no fertile fronds or structures have been observed, this remains a weak hypothesis.

***Gleicheniaceae:*** Some modern species within Gleicheniaceae also exhibit straight, oblong penultimate and ultimate segments. For instance, the modern *Diplopterygium bancroftii* (Hook.) A.R.Sm. (Plate 12E) presents these characteristics. However, many modern Gleicheniaceae species have penultimate segments with long tapering at the base, whereas in modern *Steiropteris* (Thelypteridaceae) and Fern11, there is strictly short tapering. Additionally, there is no evidence of forked segments, meaning that segments do not divide or split into two or more separate parts, in Fern11 which is a common feature of Gleicheniaceae (Jin et al., 2013). There are no character states that can completely rule out Gleicheniaceae as a hypothesis, but the features of this family do not strongly align with Fern11. Thelypteridaceae is best-supported despite both hypotheses being weak.

***Athyriaceae:*** Fern11 shares some similarities with select species of *Diplazium.* For example, the modern species *Diplazium lindbergii* (Mett.) Christ (Plate 12D) has a pinnate-pinnatifid tertiary ultimate segment structure and appears to be the closest in morphology to Fern11. However, the ultimate segment shape is not consistent between the two, where *D. lindbergii* exhibits widening at the base of ultimate segments, compared to the straight, oblong ultimate segments of Fern11. Furthermore, no fertile fronds are preserved, confirming this morphotype as Athyriaceae. Although there is a wide diversity of morphological features of modern Athyriaceae and therefore *Diplazium*, no modern species have strong consistencies with Fern11, so the hypothesis is rejected.

***Osmundaceae:*** Modern ferns in Osmundaceae share some characteristics with Fern11, such as the pinnate-pinnatifid tertiary ultimate segment structure, shown in *Osmunda claytoniana* L. (Plate 12F). However, Fern11 likely has free simple venation, a character state that is atypical of Osmundaceae, which usually has free forked venation (Zhang et al., 2013). The straight, oblong ultimate segment shape of Fern11 is inconsistent with most Osmundaceae, leading us to reject this hypothesis.

## ***Morphotype: Fern12***

***Synonymous Morphotypes:*** NA

***Nomenclature to be Evaluated:*** *Saccoloma gardneri* (Lesquereux) Knowlton

***Age of Rocks:*** Paleocene

***Morphotype Quality Index:*** Medium

***Reference Specimens:*** DMNH.EPI.51185, DMNH.EPI.51186

***Number of Specimens:*** 3

***Observed Damage Types:*** Hole feeding DT003; surface feeding DT031.

***Percent of Specimens with Herbivory/Pathogen Damage:*** 33.33%

***Study Sites:*** Denver Basin (Corral Bluffs)

***Diagnostic Characters:*** Ultimate segments long, but unknown segment shape. Venation free and forked. Sori round to kidney bean shaped, continuous and linearly arranged along the margin with true conduplicate? indusia.

***Description:*** *Segment division not observed, complete blade structure not observed, ultimate segment shape not observed but likely linear, ultimate segment tapering at the tip not observed*, *ultimate segment tapering at the base not observed*, *ultimate segment symmetry not observed,* *complete frond shape not observed,* venation free and forked, veins terminate at the margin, *vein termination in lobes/segments and clefts between segments not applicable,* margin entire, sori round to kidney bean shaped, sori continuous and linearly arranged on margin, true conduplicate shaped? indusia.

| ***Fern12 Family Assignment Hypotheses and Reference Genera*** | | |
| --- | --- | --- |
| **Hypothesis Strength** | **Family** | **Reference Genus** |
| Strong | Saccolomataceae | *Saccoloma* |
| Rejected | Pteridaceae | *Pteris* |
| Rejected | Dennstaedtiaceae | *Dennstaedtia* |

***Family Assignment Deliberations:***

***Saccolomataceae:*** Saccolomataceae is the strongest hypothesis for Fern12, and the name *Saccoloma gardneri* (Lesquereux) Knowlton is an accurate representation of the morphotype. Species in Saccolomataceae often have continuous sori along the margin and crescent to conduplicate-shaped indusia, as seen in *Saccoloma elegans* Kaulf. (Plate 13C) and in Fern12 (Plate 13B). These two also share free forked venation and long ultimate segment shapes, although ultimate segment shape is not preserved in the fossils. Because of the similarity in continuous sori along the margin and crescent-shaped indusia, we are confident that Fern12 belongs in Saccolomataceae.

***Pteridaceae:*** Fern12 has similar morphology to modern *Pteris nipponica* Shieh (Plate 13E). They have free forked venation, long ultimate segments, and reproductive structures placed linearly along the margin. The major difference is that species of *Pteris* that have sporangia along the margins do not have them arranged into sori with conduplicate-shaped indusia, leading us to reject this hypothesis.

***Dennstaedtiaceae:*** *Dennstaedtia coronata* (Sodiro) C.Chr. (Plate 13D) has defined, semi-continuous round sori along the margins with a pouch-shaped indusium, making it the most similar modern Dennstaedtiaceae to *S. gardneri*. However, most Dennstaedtiaceae, including *D. coronata,* have highly divided fronds, often with lobed segments. Because there is no strong consistency between Fern12 and Dennstaedtiaceae, we reject this hypothesis.

## ***Morphotype: Fern13***

***Synonymous Morphotypes:*** NA

***Nomenclature to be Evaluated:*** NA

***Age of Rocks:*** Cretaceous and Paleocene

***Morphotype Quality Index:*** Medium

***Reference Specimens:*** DMNH.EPI.24389, DMNH.EPI.23896

***Number of Specimens:*** 11

***Observed Damage Types:*** Margin feeding DT015; surface feeding DT027.

***Percent of Specimens with Herbivory/Pathogen Damage:*** 9.09%

***Study Sites:*** Denver Basin (Corral Bluffs, West Bijou)

**Diagnostic Characters:** Penultimate segments pinnate. Venation anastomosing. Margin entire. Sori round and linearly arranged along the margin with a true conduplicate-shaped? indusia.

**Description:** Penultimate segment pinnate, *Complete blade structure not observed*, *ultimate segment shape not observed*, *ultimate segment tapering at the tip not observed,* ultimate segment long tapering at the base, *ultimate segment symmetry not observed, penultimate segment shape not observed, penultimate segment tapering at the tip not observed, penultimate segment tapering at the base not observed, penultimate segment symmetry not observed, complete frond shape not observed,* venation anastomosing, veins terminate at the margin, *vein termination in lobes/segments and clefts between segments not applicable*, margin entire*,* sori round, sori linearly arranged along the margin, true conduplicate-shaped? indusia.

| ***Fern13 Family Assignment Hypotheses and Reference Genera*** | | |
| --- | --- | --- |
| **Hypothesis Strength** | **Family** | **Reference Genus** |
| Moderate | Saccolomataceae | *Saccoloma* |
| Weak | Pteridaceae | *Pteris* |

***Family Assignment Deliberations:***

***Saccolomataceae:*** Saccolomataceae is the closest match to Fern13 in morphology. This is because they share round, linearly arranged sori along the margin with conduplicate-shaped indusia, such as in *Saccoloma elegans* (Plate 14C). A key distinction between the two is that modern Saccolomataceae has not been observed to exhibit anastomosing venation, which is a defining character state of Fern13. Other families, such as Dennstaedtiaceae, particularly *Dennstaedtia coronata* (Plate 14E)*,* can have similar soral character states. However, the sori of this species are not linearly arranged, and this family typically has free venation. Because reproductive structure character states are often most diagnostic of a family and are prioritized in this study, Saccolomataceae is our best hypothesis, despite it being moderate in strength.

***Pteridaceae:*** A weaker familial affinity hypothesis for this morphotype is Pteridaceae. In many *Pteris* species, exemplified by *P. altissima* (Plate 14F), sporangia are linearly arranged along the margin, and the venation anastomoses. However, no *Pteris* species have linearly arranged sporangia along the margin that are congregated into discrete sori with the conduplicate indusia, as seen in Fern13, keeping this a weak hypothesis.

## ***Morphotype: Fern14***

***Synonymous Morphotypes:*** CS4 (Lyson et al., 2019)

***Nomenclature to be Evaluated:*** NA

***Age of Rocks:*** Paleocene

***Morphotype Quality Index:*** Medium

***Reference Specimens:*** DMNH.EPI.51031

***Number of Specimens:*** 1

***Observed Damage Types:*** None

***Percent of Specimens with Herbivory/Pathogen Damage:*** 0%

***Study Sites:*** Denver Basin (Corral Bluffs)

***Diagnostic Characters:*** Pinnate penultimate segments. Venation extensively forked. Penultimate and ultimate segment shapes irregular and often elliptic. Delicate, nearly transparent leaf tissue.

***Description:*** Pinnate penultimate ultimate segment, *complete blade structure not observed but likely pinnate*, ultimate segment shape irregular and elliptic?, *ultimate segment tapering at the tip not observed*, ultimate segment long tapering at the base, ultimate segments symmetrical, penultimate segment shape elliptic?, penultimate segment long? tapering at the tip, penultimate segment long tapering at the base, *penultimate segment symmetry not observed,* *complete frond shape not observed,* venation free and forked, *vein termination at the margin not observed,* *vein termination in lobes/segments and clefts between segments not applicable*, margin irregular?, *reproductive structures not observed.*

| ***Fern14 Family Assignment Hypotheses and Reference Subfamilies & Genera*** | | |
| --- | --- | --- |
| **Hypothesis Strength** | **Family** | **Reference Subfamily & Genus** |
| Strong | Hymenophyllaceae | Trichomanoideae, *Didymoglossum*; Hymenophylloideae, *Hymenophyllum* |

***Family & Subfamily Assignment Deliberations:***

***Hymenophyllaceae:*** Hymenophyllaceae is the strongest and most likely hypothesis for Fern14. This family is known for their highly branched free, often forked veins and very thin, translucent fronds and has no inconsistent character states with Fern14. The placement of this morphotype was also investigated at the subfamily level, considering the two defined subfamilies of Hymenophyllaceae: Trichomanoideae and Hymenophylloideae (Pteridophyte Phylogeny Group, 2016). Trichomanoideae may be a slightly stronger hypothesis, due to Fern14’s similarity to the modern *Didymoglossum godmanii* (Hook. ex Baker) Ebihara & Dubuisson (Plate 15B). In contrast, many species within Hymenophylloideae (Plate 15C) have very reduced, minimal leaf tissue and are often highly segmented, although *D. godmanii* demonstrates that some species in Trichomanoideae retain more substantial leaf tissue. Because Trichomanoideae and Hymenophylloideae are morphologically distinguished by the shape of their sori (Pryer et al., 2001), though, we conclude that the subfamily of Fern14 cannot be determined.

## ***Morphotype: Fern15***

***Synonymous Morphotypes:*** CS12 (Lyson et al., 2019)

***Nomenclature to be Evaluated:*** NA

***Age of Rocks:*** Paleocene

***Morphotype Quality Index:*** Medium

***Reference Specimens:*** DMNH.EPI.49735, DMNH.EPI.50995

***Number of Specimens:*** 6

***Observed Damage Types:*** None

***Percent of Specimens with Herbivory/Pathogen Damage:*** 0%

***Study Sites:*** Denver Basin (Corral Bluffs, Plum Creek Parkway)

***Diagnostic Characters:*** Tertiary ultimate segment pinnate-pinnatifid. Leaf tissue reduced to narrow strips surrounding veins. Tertiary ultimate and penultimate segment shape irregular. Ultimate segments oblong to elliptic. Venation free and forked. Sori cone-shaped on the margins at the termination of veins.

***Description:*** Tertiary ultimate segment pinnate-pinnatifid, *complete blade structure not observed*, ultimate segment shape oblong to elliptic, ultimate segment short tapering at the tip, *ultimate segment tapering at the base not applicable*, ultimate segments symmetrical, penultimate segment shape irregular, penultimate segment short tapering at the tip, penultimate segment long tapering at the base, *penultimate segment symmetry not observed,* tertiary ultimate segment shape irregular, tertiary ultimate segment short tapering at the tip, tertiary ultimate segment long tapering at the base, *complete frond shape not observed*, venation free and forked, veins terminate at the margin, vein termination in lobes/segments only, margin entire, sori cone-shaped, sori discrete and not continuous on margin and at the termination of veins, *indusia not observed but like true cup-shaped indusia.*

| ***Fern15 Family Assignment Hypotheses and Reference Subfamilies & Genera*** | | |
| --- | --- | --- |
| **Hypothesis Strength** | **Family** | **Reference Subfamily & Genus** |
| Strong | Hymenophyllaceae | Trichomanoideae, *Crepidomanes* |
| Rejected | Pteridaceae | *Onychium* |

***Family Assignment Deliberations:***

***Hymenophyllaceae:*** Ferns in the family Hymenophyllaceae are often very delicate and have highly branched segments. Their veins are extensively forked, often with singular veins terminating in lobes/ultimate segments. Because these character states are overwhelmingly consistent, we can confidently place Fern15 within Hymenophyllaceae.

DMNH.EPI.49735 (Plate 16B) likely exhibits cone-shaped sori on the margins at vein terminations. They have likely cup-shaped indusia and/or two involucres that form the indusium. Although uncertainty regarding the sori of Fern15 precludes us from confidently place Fern15 into a subfamily, we favor Trichomanoideae over Hymenophylloideae because we interpret Fern15 as having two darkened bracts surrounding each sorus, resembling *Crepidomanes pyxidiferum* (L.) Dubuisson & Ebihara (Plate 16C).

***Pteridaceae:*** *Onychium japonicum* (Thunb.) Kunze (Plate 16E) has a similar blade structure and segmentation to Fern15. They are dissimilar in the pinnatifid structure of the penultimate segments. More importantly, the sori are inconsistent with those of Pteridaceae. Because the blade structure and segmentation of Fern15 are not typical of Pteridaceae and their reproductive structures are inconsistent, we can exclude this family as a possible classification.

## ***Morphotype: Fern16***

***Synonymous Morphotypes:*** CS14 (Lyson et al., 2019)

***Nomenclature to be Evaluated:*** NA

***Age of Rocks:*** Paleocene

***Morphotype Quality Index:*** Low

***Reference Specimens:*** DMNH.EPI.51244

***Number of Specimens:*** 1

***Observed Damage Types:*** None

***Percent of Specimens with Herbivory/Pathogen Damage:*** 0%

***Study Sites:*** Denver Basin (Corral Bluffs)

***Diagnostic Characters:*** Penultimate segments pinnatifid. Penultimate segment flabellate (approx. 8 cm in length and 4 cm in width) and ultimate segments/lobes oblong. Venation free and forked, radiating from base of penultimate segment.

***Description:*** Penultimate segments pinnatifid, *complete blade structure not observed*, ultimate segment shape oblong, ultimate segment short tapering at the tip, *ultimate segment tapering at the base not applicable,* *ultimate segment symmetry not applicable,* penultimate segment flabellate, penultimate segment short tapering at the tip, *penultimate segment tapering at the base not observed*, *penultimate segment symmetry not applicable,* *complete frond shape not observed,* venation free and forked, vein termination at the margin, vein termination in lobes/segments and clefts between segments, margin entire, *reproductive structures not observed.*

| ***Fern16 Family Assignment Hypotheses and Reference Genera*** | | |
| --- | --- | --- |
| **Hypothesis Strength** | **Family** | **Reference Genus** |
| Weak | Hymenophyllaceae | *Didymoglossum* |

***Family Assignment Deliberations:***

***Hymenophyllaceae:*** Fern16 appears somewhat similar to the modern *Didymoglossum* (Plate 17C) in Hymenophyllaceae. This is due to the radiating venation from the base to the tip of the penultimate segment. Fern16 also appears to be lobed or have ultimate segments that are not deeply incised. The margin is entire, albeit irregular. However, the only specimen of this morphotype is poorly preserved and does not have characters such as reproductive structures that can definitively diagnose it.

## ***Morphotype: Fern17***

***Synonymous Morphotypes:*** NA

***Nomenclature to be Evaluated:*** NA

***Age of Rocks:*** Paleocene

***Morphotype Quality Index:*** Low

***Reference Specimens:*** DMNH.EPI.51246

***Number of Specimens:*** 1

***Observed Damage Types:*** None

***Percent of Specimens with Herbivory/Pathogen Damage:*** 0%

***Study Sites:*** Denver Basin (Corral Bluffs)

***Diagnostic Characters:*** Ultimate segments flabellate (approx. 3 cm in length and 6 cm in width). Venation free and forked, radiating outward from the base to the tip of the ultimate segment.

***Description:*** *Segment division not observed, complete blade structure not observed,* ultimate segment flabellate, ultimate segment short tapering at the tip, ultimate segment short tapering at the base, *ultimate segment symmetry not observed, complete frond shape not observed,* venation free and forked, veins terminate at the margin, *vein termination in lobes/segments and clefts between segments not applicable, margin type not observed, reproductive structures not observed.*

| ***Fern17 Family Assignment Hypotheses and Reference Genera*** | | |
| --- | --- | --- |
| **Hypothesis Strength** | **Family** | **Reference Genus** |
| Weak | Schizaeaceae | *Schizaea* |
| Weak | Hymenophyllaceae | *Didymoglossum* |
| Weak | Pteridaceae | *Adiantum* |

***Family Assignment Deliberations:***

***Schizaeaceae:*** Schizaeaceae is the most likely hypothesis for Fern17, but it is not much stronger than competing hypotheses. This family has species with flabellate ultimate segments with radiating free-forked venation. In the single specimen of this morphotype, the apical margin is not preserved in the ultimate segment. Sori are typically located along this margin in species of *Schizaea* (Plate 17D). Due to the delicate attachment of this family’s sori, it is likely that they may not be preserved on fossils. The fragility of the reproductive structures in Schizaeaceae may provide an explanation as to why the margin itself was poorly preserved. Regardless of the reproductive structure character states, all other character states are consistent with Schizaeaceae.

***Hymenophyllaceae:*** Within modern Hymenophyllaceae, Fern17 appears to be most similar to *Didymoglossum membranaceum* (L.) Vareschi (Plate 17C)*.* This species also has radiating free forked venation and flabellate ultimate segments. *D. membranaceum* has sori along the apex margin of the frond/ultimate segment. Additionally, the lamina of this species is two cells thick, which may explain the near-transparent preserved lamina of the fossil.

***Pteridaceae:*** Modern *Adiantum andicola* Liebm. (Plate 17E) of Pteridaceae has radiating free forked venation and flabellate ultimate segments like Fern17. Again, the genus *Adiantum* often has sori along the margin of the apex of ultimate segments with a false indusium. The size of ultimate segments in *Adiantum* tend to be much smaller than the large ultimate segment of Fern17, however, this is enough evidence to reject a Pteridaceae hypothesis.

## ***Morphotype: Fern18***

***Synonymous Morphotypes:*** CS5 (Lyson et al., 2019)

***Nomenclature to be Evaluated:*** *Dryopteris lakesii* (Lesquereux) Knowlton

***Age of Rocks:*** Cretaceous and Paleocene

***Morphotype Quality Index:*** High

***Reference Specimens:*** DMNH.EPI.40407, DMNH.EPI.51331, DMNH.EPI.51250

***Number of Specimens:*** 95

***Observed Damage Types:*** Hole feeding DT001, DT002, DT003; margin feeding DT012, DT014.

***Percent of Specimens with Herbivory/Pathogen Damage:*** 6.32%

***Study Sites:*** Denver Basin (Corral Bluffs, West Bijou, Plum Creek Parkway), Raton Basin, Williston Basin

***Diagnostic Characters:*** Tertiary ultimate segments bipinnatifid. Lobes transition to more deeply incised ultimate segments from the apex of the frond to the base. Margins serrate. Sori round and centered between the axial vein of the ultimate segment and the ultimate segment margin.

***Description:*** Tertiary ultimate segment bipinnatifid, *complete blade structure not observed but likely bipinnatifid*, ultimate segment shape elliptic to linear, ultimate segment long tapering at the tip, *ultimate segment tapering at the base not applicable*, ultimate segments symmetrical, penultimate segment shape elliptic to linear, penultimate segment long tapering at the tip, *penultimate segment tapering at the base not applicable*, penultimate segments symmetrical, *tertiary ultimate segment shape not observed*, tertiary ultimate segment long tapering at the tip, *tertiary ultimate segment tapering at the base not observed,* *complete frond shape not observed,* venation free and simple, veins terminate at the margin, vein termination in lobes/segments only, margin serrate, sori round, sori centered between ultimate segment axial vein and margin of ultimate segments, *indusia not observed.*

| ***Fern18 Family Assignment Hypotheses and Reference Genera*** | | |
| --- | --- | --- |
| **Hypothesis Strength** | **Family** | **Reference Genus** |
| Strong | Dryopteridaceae | *Dryopteris* |
| Weak | Cyatheaceae | *Cyathea* |
| Rejected | Tectariaceae | *Tectaria* |

***Family Assignment Deliberations:*** This morphotype closely aligns with the morphology of *Dryopteris lakesii* (Lesquereux) Knowlton, which was previously associated with Polypodiaceae (Knowlton, 1930). More recently, modern classifications have placed *Dryopteris* within the family Dryopteridaceae (Pteridophyte Phylogeny Group, 2016). Due to the stem age of Polypodiaceae post-dating the Cretaceous-Paleogene boundary (Pelosi et al., 2022) and the reworking of the familial affiliation of *Dryopteris*, Polypodiaceae has not been considered in our study as a viable hypothesis. Instead, Dryopteridaceae has been investigated. The identification of two fertile specimens (Plate 18A, Plate 18D, DMNH.EPI.40407; Plate 18B, DMNH.EPI.51331) provides support for a Dryopteridaceae hypothesis of Fern18.

***Dryopteridaceae:*** Modern Dryopteridaceae often have highly divided fronds like Fern18. Serrate margins that transition to lobes, then to ultimate segments moving from the apex to the base of the frond are common in this family. The sori of Dryopteridaceae have a variety of shapes (Smith, 2020a); in *Dryopteris*, sori are typically round (Montgomery and Wagner, 2020). Although not identical, modern *Dryopteris campyloptera* Clarkson (Plate 18F) is a good analog for Fern18. Their serrate margins transition to lobes and ultimate segments toward the base of the tertiary ultimate segment, like in Fern18. *D. camplyoptera* also has a mix of sterile and fertile segments on a single frond, also observed in DMNH.EPI.51331 (Plate 18B). Although the presence of true indusia in Fern18 is uncertain due to preservation, it appears plausible in DMNH.EPI.40407 (Plate18D), with a small, round, light marking on the dark sori. This is consistent with the small, round indusia of *D. camplyoptera.*

The segment symmetry across axial veins of Fern18 is suggested in our descriptions to be symmetrical. This notion cannot be completely confirmed because the entire frond of this morphotype has not been preserved in any specimen. Asymmetrical segments are a common characteristic of members in Dryopteridaceae, particularly *Dryopteris*, but segment symmetry and asymmetry are both observed in this family and genus*.* So, if Fern18 has only symmetrical segments, this does not discount the validity of a Dryopteridaceae hypothesis. We suggest that the nomenclature of *Dryopteris lakesii* be maintained as there is strong evidence for Dryopteridaceae.

***Cyatheaceae:*** Cyatheaceae was considered as an alternative hypothesis in the survey we conducted. Many members of this family such as *Cyathea multiflora* Sm. (Plate 18E) have a serrate margin, free venation, highly divided fronds, and round sori centered between the ultimate segment axial vein and margin. However, the transition of serrations to lobes to ultimate segments down the frond is typically not as abrupt as in Fern18 and in many species of *Dryopteris.* Cyatheaceae has a wide variety of the appearance of their sori. They are typically large, round, and have diverse indusium types. Fern18 is hypothesized to possess scale-like true indusia, which is a character state observed in Cyatheaceae (Zhang and Nishida, 2013) but is not typical of this family. Because there are no inconsistencies between Fern18 and the overall description of Cyatheaceae, this family cannot be completely ruled out as a hypothesis. But, this family does not have a striking resemblance to Fern18 as observed in Dryopteridaceae, making this a weak hypothesis.

***Tectariaceae:*** *Tectaria mexicana* (Fée) C.V.Morton (Plate 18F) is the most similar modern representative of Tectariaceae to Fern18. This species has a highly divided frond and round sori, like Fern18. However, the genus *Tectaria,* which contains the majority of modern Tectariaceae species (Pteridophyte Phylogeny Group, 2016), typically has anastomosing venation (Moran, 2020), whereas Fern18 has free simple venation. In addition, *T. mexicana*, as our best modern equivalent to Fern18, has sori scattered on ultimate and penultimate segments, rather than centered between the ultimate segment axial vein and margin. Because of these inconsistencies, the likelihood of a Tectariaceae affiliation is minimal.

## ***Morphotype: Fern19***

***Synonymous Morphotypes:*** CS221 (Lyson et al., 2019)

***Nomenclature to be Evaluated:*** NA

***Age of Rocks:*** Paleocene

***Morphotype Quality Index:*** High

***Reference Specimens:*** DMNH.EPI.51030

***Number of Specimens:*** 1

***Observed Damage Types:*** None

***Percent of Specimens with Herbivory/Pathogen Damage:*** 0%

***Study Sites:*** Denver Basin (Corral Bluffs)

***Diagnostic Characters:*** Tertiary ultimate segment pinnate-pinnatifid. penultimate segment shape linear and ultimate segment shape oblong. Venation free and forked. Sori impressions small (<0.5 mm diameter), round and centered between the axial vein of the ultimate segment and margin. Thick rachis.

***Description:*** Tertiary ultimate segment pinnate-pinnatifid, *complete blade structure not observed but likely pinnate-pinnatifid*, ultimate segment shape oblong, ultimate segment short tapering at the tip, *ultimate segment tapering at the base not applicable*, ultimate segments symmetrical, penultimate segment shape linear, penultimate segment long tapering at the tip, penultimate segment short tapering at the base, penultimate segments symmetrical, *tertiary ultimate segment shape not observed*, *tertiary ultimate segment tapering at the tip not observed, tertiary ultimate segment tapering at the base not observed, complete frond shape not observed,* venation free and forked, veins terminate at the margin, vein termination in lobes/segments only, margin entire, sori round, sori centered between ultimate segment axial vein and margin, *indusia not observed.*

| ***Fern19 Family Assignment Hypotheses and Reference Genera*** | | |
| --- | --- | --- |
| **Hypothesis Strength** | **Family** | **Reference Genus** |
| Weak | Cyatheaceae | *Cyathea* |
| Weak | Dryopteridaceae | *Dryopteris* |

***Family Assignment Deliberations:***

***Cyatheaceae:*** Fern19 is similar to the modern *Cyathea bipinnatifida* (Plate 19H). Both share a thick rachis, free and forked venation, nearly identical ultimate and penultimate segment shapes, and small, round sori lined near the ultimate segment axial veins. There are no apparent differences between the two; however, impressions of sori, rather than preserved sori themselves, are present on DMNH.EPI.51030 (Plate 19D). This prevents assignment of some character states and may give a misleading interpretation of the character states of the sori that were scored. Cyatheaceae is our strongest hypothesis, but it remains weak because the character states observed are not particularly diagnostic for this family.

***Dryopteridaceae:*** Dryopteridaceae is an alternative hypothesis for Fern19. *Dryopteris polylepis* (Franch. & Sav.) C.Chr. (Plate 19G) has similar ultimate segment shapes, round sori, and a thick rachis. A difference between the two is that *D. polylepis* has sharp, long tapering toward the tip of the penultimate segment. Further, many species of *Dryopteris* have highly divided fronds or lobed segments. Dryopteridaceae cannot be completely ruled out, though, because there are no character states that can explicitly distinguish them.

## ***Morphotype: Fern20***

***Synonymous Morphotypes:*** NA

***Nomenclature to be Evaluated:*** *Dryopteris integra* Knowlton

***Age of Rocks:*** Cretaceous

***Morphotype Quality Index:*** Low

***Reference Specimens:*** DMNH.EPI.21785, DMNH.EPI.21790

***Number of Specimens:*** 4

***Observed Damage Types:*** None

***Percent of Specimens with Herbivory/Pathogen Damage:*** 0%

***Study Sites:*** Denver Basin (Corral Bluffs), Williston Basin

***Diagnostic Characters:*** Ultimate segments lanceolate to linear with long tapering at the tip. Sori round and centered between ultimate segment midvein and margin. True indusium, round.

***Description:*** Penultimate segment pinnate?, *complete blade structure not observed*, ultimate segment shape lanceolate to linear, ultimate segment long tapering at the tip, ultimate segment short tapering at the base, ultimate segments symmetrical, *penultimate segment shape not observed*, *penultimate segment tapering at the tip not observed, penultimate segment tapering at the base not observed,* *penultimate segment symmetry not observed,* *complete frond shape not observed*, *venation not observed*, margin entire?, sori round, sori centered between ultimate segment axial vein and margin, true indusia round.

| ***Fern20 Family Assignment Hypotheses and Reference Genera*** | | |
| --- | --- | --- |
| **Hypothesis Strength** | **Family** | **Reference Genus** |
| Moderate | Dryopteridaceae | *Dryopteris* |

***Family Assignment Deliberations:***

***Dryopteridaceae:*** This morphotype is likely the same as the fossil *Dryopteris integra* Knowlton (Knowlton 1930). The most striking character states of this morphotype are the round sori with their true round indusia that resemble modern *Dryopteris wallichiana* (Spreng.) Hyl. (Plate 20E)*.* Some species of *Dryopteris* are described to exhibit laterally attached indusia and can be persistent or caducous, meaning that the indusia can stay attached or fall off as the sori mature (Montgomery and Wagner, 2020).

Specimens DMNH.EPI.21785 (Plate 20A, Plate 20B) and DMNH.EPI.21790 (Plate 20C) exhibits sori with various stages of indusium detachment. Different sori on the fossil have laterally attached indusia, partially detached indusia that remain on the sorus, and indusia that have likely completely detached at maturity. These stages on singular fossils support a Dryopteridaceae hypothesis. Because of the poor preservation of the lamina in all specimens, the character states of the margin and veins cannot be scored, preventing definitive confirmation of classification.

## ***Morphotype: Fern21***

***Synonymous Morphotypes:*** CS11

***Nomenclature to be Evaluated:*** NA

***Age of Rocks:*** Cretaceous and Paleocene

***Morphotype Quality Index:*** Low

***Reference Specimens:*** DMNH.EPI.23739, DMNH.EPI.34379

***Number of Specimens:*** 2

***Observed Damage Types:*** Skeletonization DT016.

***Percent of Specimens with Herbivory/Pathogen Damage:*** 50%

***Study Sites:*** Denver Basin (Corral Bluffs, Plum Creek Parkway)

***Diagnostic Characters:*** Ultimate segments large, but unknown size range. Ultimate segments with long, triangular tapering at the tip. Venation anastomosing. Margin serrate.

***Description:*** *Segment division not observed, complete blade structure not observed, ultimate segment shape not observed*, ultimate segment long tapering at the tip, *ultimate segment tapering at the base not observed*, *ultimate segment symmetry not observed, complete frond shape not observed,* venation anastomosing, veins terminate at the margin, *vein termination in lobes/segments and clefts between segments not applicable*, margin serrate to crenate, *reproductive structures not observed*.

| ***Fern21 Family Assignment Hypotheses and Reference Genera*** | | |
| --- | --- | --- |
| **Hypothesis Strength** | **Family** | **Reference Genus** |
| Weak | Dryopteridaceae | *Cyrtomium* |
| Rejected | Athyriaceae | *Diplazium* |

***Family Assignment Deliberations:***

***Dryopteridaceae****:* Due to the fragmentary nature of the observed specimens and lack of reproductive structures, it is challenging to place Fern21 into a family confidently. The sharp point of the apex of ultimate segments, the anastomosing venation, and the serrate to crenate margin are the only character states preserved for this morphotype. These are characters that are all consistent with *Cyrtomium* (Plate 19E) in Dryopteridaceae. Other character states that distinguish *Cyrtomium* are their rows of round sori between the axial vein and margin, and often an inequilateral base of ultimate segments (Yatskievych, 2020). Either of these character states would need to be preserved to make this a stronger hypothesis.

***Athyriaceae:*** *Diplazium* (Plate 19F) was suggested in our survey due to the long tapering at the tip of the preserved segments. However, members of Athryriaceae rarely have anastomosing venation (Wang et al., 2013), making this family an unlikely candidate.

## ***Morphotype: Fern22***

***Synonymous Morphotypes:*** NA

***Nomenclature to be Evaluated:*** NA

***Age of Rocks:*** Cretaceous and Paleocene

***Morphotype Quality Index:*** Low

***Reference Specimens:*** YPMPB.061361, YPMPB.041985

***Number of Specimens:*** 8

***Observed Damage Types:*** None

***Percent of Specimens with Herbivory/Pathogen Damage:*** 0%

***Study Sites:*** Denver Basin

***Diagnostic Characters:*** Bipinnate-pinnatifid quaternary ultimate segments. Penultimate and ultimate segments asymmetrical. Venation free and forked.

***Description:*** Bipinnate-pinnatifid quaternary ultimate segment, *complete blade structure not observed,* ultimate segment shape elliptic, ultimate segment long tapering at the tip, *ultimate segment tapering at the base not applicable,* ultimate segments asymmetrical, penultimate segment shape ovate to elliptic, penultimate segment long tapering at the tip, penultimate segment long tapering at the base, penultimate segments asymmetrical, *tertiary ultimate segment shape not observed,* tertiary ultimate segment long tapering at the tip, tertiary ultimate segment long tapering at the base, *quaternary ultimate segment shape not observed, quaternary ultimate segment tapering at the tip not observed, quaternary ultimate segment tapering at the base not observed, complete frond shape not observed,* venation free and forked, terminating at the margin, vein termination in lobes/segments only, margin entire?, *reproductive structures not observed.*

| ***Fern22 Family Assignment Hypotheses and Reference Genera*** | | |
| --- | --- | --- |
| **Hypothesis Strength** | **Family** | **Reference Genus** |
| Moderate | Dryopteridaceae | *Dryopteris* |
| Moderate | Saccolomataceae | *Saccoloma* |
| Weak | Athyriaceae | *Diplazium* |
| Weak | Dennstaedtiaceae | *Hypolepis* |

***Family Assignment Deliberations:*** We acknowledge uncertainty regarding whether all specimens assigned to this group represent a cohesive morphotype. There are small inconsistencies between specimens that lead to this uncertainty, but there are no prominent character states identified that justify separate distinct morphotypes. As a result, we recognize that this morphotype could encompass multiple taxa. For the purpose of family assignment, we have selected YPMPB.061361 (Plate 21A) to represent this morphotype with classifications focused on this specimen.

***Dryopteridaceae:*** Fern22 is fairly consistent with modern Dryopteridaceae (Plate 21C), with highly divided fronds and free forked venation commonly observed in this family. Based on YPMPB.061361 (Plate 21A), some of the ultimate and penultimate segments are asymmetrical across axial veins. Asymmetry of segments supports a Dryopteridaceae hypothesis, however, this character state is also observed in the other families considered. Because evidence for this character state is weak and the reproductive structures have not been observed, Dryopteridaceae is a moderate hypothesis.

***Saccolomataceae:*** Fern22 resembles modern *Saccoloma inaequale* (Kunze) Mett. (Plate 21D). Saccolomataceae is equally as viable of a hypothesis as Dryopteridaceae due to highly divided fronds, asymmetrical segments, free forked venation, and ultimate and penultimate segment shapes. Because the overall frond morphology within both of these two groups can have difficult to distinguish character states (other than reproductive structures), Saccolomataceae is also a moderate hypothesis.

***Athyriaceae:*** Athyriaceae, as a hypothesis, is consistent with Fern22 for similar grounds as for Dryopteridaceae and Saccolomataceae. Many members of this family, like *Diplazium prominulum* (Plate 21E), have highly divided fronds, free forked venation, and asymmetrical segments. Asymmetrical segments, however, are not typical of *Diplazium* or Athryiaceae, making this a somewhat weaker hypothesis.

***Dennstaedtiaceae:*** *Hypolepis hostilis* (Kunze) C.Presl (Plate 21F) of Dennstaedtiaceae also has asymmetrical penultimate segments to Fern22. Other character states, like highly divided fronds and the venation type, are in common as well. The ultimate and penultimate segment shapes of Fern22 seem more consistent with species of Dryopteridaceae or Saccolomataceae, though, making Dennstaedtiaceae a weaker hypothesis.

## ***Morphotype: Fern23***

***Synonymous Morphotypes:*** NA

***Nomenclature to be Evaluated:*** NA

***Age of Rocks:*** Paleocene

***Morphotype Quality Index:*** Medium

***Reference Specimens:*** DMNH.EPI.51954, DMNH.EPI.51952

***Number of Specimens:*** 7

***Observed Damage Types:*** None

***Percent of Specimens with Herbivory/Pathogen Damage:*** 0%

***Study Sites:*** Williston Basin

***Diagnostic Characters:*** Tertiary ultimate segment pinnate-pinnatifid. Ultimate segments ovate. Venation free and forked. Margin serrate.

***Description:*** Tertiary ultimate segment pinnate-pinnatifid, *complete blade structure not observed*, ultimate segment shape ovate, ultimate segment long tapering at the tip, *ultimate segment tapering at the base not applicable,* ultimate segments symmetrical, *penultimate segment shape not observed,* penultimate segment long tapering at the tip, penultimate segment long tapering at the base, *penultimate segment symmetry not observed, tertiary ultimate segment shape not observed, tertiary ultimate segment tapering at the tip not observed, tertiary ultimate segment tapering at the base not observed, complete frond shape not observed*, venation free and forked, veins terminate at the margin, vein termination in lobes/segments and clefts between segments, margin serrate, *reproductive structures not observed.*

| ***Fern23 Family Assignment Hypotheses and Reference Genera*** | | |
| --- | --- | --- |
| **Hypothesis Strength** | **Family** | **Reference Genus** |
| Weak | Dryopteridaceae | *Dryopteris* |
| Weak | Athyriaceae | *Athyrium* |

***Family Assignment Deliberations:***

**Dryopteridaceae:** Fern23 has several morphological features similar to modern *Dryopteris cristata* Watts (Plate 22C) with their kinked costa and costules; however, this could be preservational in the fossils. The tapering of the penultimate segment is comparable as well. However, while these traits are consistent with *Dryopteris*, they are not particularly distinctive to this family. Dryopteridaceae might be our best hypothesis, but it only has weak support because reproductive structures have not been observed.

**Athyriaceae:** This morphotype also looks like the modern *Athyrium vidalii* (Franch. & Sav.) Nakai (Plate 22D) because of the shared shape of the penultimate segment and free forked venation. The venation for this genus does not seem as dense as what is typically found in *Dryopteris*, making this a somewhat weaker hypothesis. Without reproductive structures, neither hypothesis has strong support.

## ***Morphotype: Fern24***

***Synonymous Morphotypes:*** NA

***Nomenclature to be Evaluated:*** NA

***Age of Rocks:*** Cretaceous

***Morphotype Quality Index:*** Low

***Reference Specimens:*** DMNH.EPI.23839

***Number of Specimens:*** 2

***Observed Damage Types:*** None

***Percent of Specimens with Herbivory/Pathogen Damage:*** 0%

***Study Sites:*** Denver (Corral Bluffs)

***Diagnostic Characters:*** Tripinnate quaternary ultimate segment with likely a triangular-pentagonal shape. Tertiary ultimate segments ovate or elliptic, penultimate and ultimate segments oblong to linear. Margin entire.

***Description:*** Quaternary ultimate segment tripinnate, *complete blade structure not observed but likely tripinnate*, ultimate segment shape oblong, ultimate segment short tapering at the tip, ultimate segment short tapering at the base, symmetrical ultimate segments, penultimate segment shape oblong to linear, penultimate segment long tapering at the tip, penultimate segment long? tapering at the base, symmetrical penultimate segments, tertiary ultimate segment ovate or elliptic, tertiary ultimate segment long tapering at the tip, *tertiary ultimate segment tapering at the base not observed*, *quaternary ultimate segment shape not observed but likely star shaped, complete frond shape/quaternary ultimate segment shape not observed but likely triangular-pentagonal,* *frond/quaternary ultimate segment tapering at the tip not observed but likely long, frond/quaternary ultimate segment tapering at the base not observed but likely short, venation not observed*, margin entire, *reproductive structures not observed.*

| ***Fern24 Family Assignment Hypotheses and Reference Genera*** | | |
| --- | --- | --- |
| **Hypothesis Strength** | **Family** | **Reference Genus** |
| Moderate | Pteridaceae | *Pentagramma* |

***Family Assignment Deliberations:***

***Pteridaceae:*** The triangular-pentagonal shape of this morphotype’s blade suggests that it is most like the modern genus *Pentagramma* (Plate 23B) in Pteridaceae (Yatskievych and Windham, 2020). The approximately five tertiary ultimate segments of Fern24 appear to be triangular in shape and meet together palmately to form the blade. The specimens of this morphotype all have poor preservation, with the venation type and reproductive structures not observed. Although other genera such as *Notholaena* (Plate 23C) exhibit the triangular-pentagonal shape, this character state is restricted to Pteridaceae. The low MQI of the morphotypes restricts it to a moderate hypothesis.

## ***Morphotype: Fern25***

***Synonymous Morphotypes:*** NA

***Nomenclature to be Evaluated:*** NA

***Age of Rocks:*** Cretaceous and Paleocene

***Morphotype Quality Index:*** Low

***Reference Specimens:*** YPMPB.066250

***Number of Specimens:*** 2

***Observed Damage Types:*** None

***Percent of Specimens with Herbivory Damage:*** 0%

***Study Sites:*** Williston Basin

***Diagnostic Characters:*** Penultimate segment pinnatifid. Penultimate segment lanceolate (approx. 11 cm in length and 3 cm in width), ultimate segment elliptic. Margin entire.

***Description:*** Penultimate segment pinnatifid, *complete blade structure not observed*, ultimate segment shape elliptic, ultimate segment long tapering at the tip, *ultimate segment tapering at the base not applicable,* ultimate segment symmetrical, penultimate segment shape lanceolate, penultimate segment long tapering at the tip, penultimate segment long tapering at the base, *penultimate segment symmetry not observed*, *tertiary ultimate segment shape not observed, tertiary ultimate segment tapering at the tip not observed, tertiary ultimate segment tapering at the base not observed, complete frond shape not observed*, *venation not observed*, margin entire, *reproductive structures not observed.*

| ***Fern25 Family Assignment Hypotheses and Reference Genera*** | | |
| --- | --- | --- |
| **Hypothesis Strength** | **Family** | **Reference Genus** |
| Weak | Pteridaceae | *Pteris* |
| Rejected | Osmundaceae | *Osmunda* |

***Family Assignment Deliberations:***

***Pteridaceae:*** Fern25 resembles modern *Pteris pungens* Krug (Plate 23E), with similarities in the shape of the ultimate and penultimate segments. However, the absence of preserved venation and reproductive structures prevents further evaluation in assigning Fern25 to a family. Pteridaceae is a weak hypothesis.

***Osmundaceae:*** Osmundaceae is a rejected hypothesis because the segment shapes do not strongly resemble modern species of this family. Segment shape is not particularly diagnostic, and more characters and states need to be preserved to place Fern25 confidently into a family.

## ***Morphotype: Fern26***

***Synonymous Morphotypes:*** NA

***Nomenclature to be Evaluated:*** NA

***Age of Rocks:*** Paleocene

***Morphotype Quality Index:*** Medium

***Reference Specimens:*** DMNH.EPI.40371, DMNH.EPI.40372

***Number of Specimens:*** 3

***Observed Damage Types:*** Hole feeding DT001; margin feeding DT015; pathogen/environment DT058.

***Percent of Specimens with Herbivory/Pathogen Damage:*** 33.33%

***Study Sites:*** Denver Basin (Corral Bluffs), Williston Basin

***Diagnostic Characters:*** Ultimate segments elliptic with a base that tapers outward with a slight curved upturn (approx 1-2 cm in length and 0.5 cm in width). Venation free and forked. Margin entire.

***Description:*** Penultimate segment pinnatifid?, *complete blade structure not observed*, ultimate segment shape elliptic, ultimate segment long tapering at the tip, *ultimate segment tapering at the base not applicable,* *ultimate segment symmetry not observed,* penultimate segment shape lanceolate, penultimate segment long tapering at the tip, penultimate segment short tapering at the base, *penultimate segment symmetry not observed, complete frond shape not observed,* venation free and forked, veins terminate at the margin, vein termination in lobes/segments only, margin entire, *reproductive structures not observed.*

| ***Fern26 Family Assignment Hypotheses and Reference Genera*** | | |
| --- | --- | --- |
| **Hypothesis Strength** | **Family** | **Reference Genus** |
| Weak | Nephrolepidaceae | *Nephrolepis* |
| Weak | Pteridaceae | *Pteris* |

***Family Assignment Deliberations:***

***Nephrolepidaceae:*** Modern *Nephrolepis* *undulata* (Afzel. ex Sw.) J.Sm. (Plate 26D) shares a similar venation and ultimate segment shape as Fern26. In species of *Nephrolepis,* ultimate segments are often articulated to the rachis or the penultimate segment axial vein (Nauman, 2020b)*.* Articulation refers to the phenomenon in which the base of the ultimate segments is not completely fused to the penultimate segment axial vein. Instead, ultimate segments are attached only at the ultimate segment axial vein, and the base appears to be wide or notched. Due to preservation constraints, it is challenging to definitively suggest that ultimate segment articulation is a character state present in Fern26. However, articulation may be observed in specimens like DMNH.EPI.40371 (Plate 26B). Reproductive structures have not been observed to confirm a Nephrolepidaceae classification.

***Pteridaceae:*** *Pteris* has similar morphology to Fern26, and with *Nephrolepis* as well*.* A key distinction that would support a Pteridaceae hypothesis would be the conclusion that the penultimate segment is pinnatifid, rather than having ultimate segments that are articulate to the penultimate segment axial vein. Because this character state is uncertain, there is no strong evidence to support one hypothesis over the other.

## ***Morphotype: Fern27***

***Synonymous Morphotypes:*** CS10 (Lyson et al., 2019)

***Nomenclature to be Evaluated:*** *Salvinia* sp.

***Age of Rocks:*** Cretaceous

***Morphotype Quality Index:*** Medium

***Reference Specimens:*** DMNH.EPI.23743

***Number of Specimens:*** 2

***Observed Damage Types:*** None

***Percent of Specimens with Herbivory/Pathogen Damage:*** 0%

***Study Sites:*** Denver Basin (Corral Bluffs)

***Diagnostic Characters:*** Fronds simple and paired. Blades orbicular in shape. Trichomes on blade surface.

***Description:*** Blade structure simple, frond/ultimate segment shape orbicular, frond/ultimate segment short tapering at the tip, frond/ultimate segment short tapering at the base, *ultimate segment symmetry not applicable*, *venation not observed*, margin entire, *reproductive structures not preserved but likely sporocarps.*

| ***Fern27 Family Assignment Hypotheses and Reference Genera*** | | |
| --- | --- | --- |
| **Hypothesis Strength** | **Family** | **Reference Genus** |
| Strong | Salviniaceae | *Salvinia* |

***Family Assignment Deliberations:***

***Salviniaceae:*** Fern27 appears nearly identical to modern species of Salviniaceae, particularly *Salvinia minima* Baker (Plate 25D). Both have paired blades with orbicular shapes. Additionally, preserved indentations on Fern27 likely represent trichome impressions that are a characteristic feature of *Salvinia*. There are no morphological traits that would suggest Fern27 belongs to a different family. Based on the placement of Fern27 within Salviniaceae, we can confidently infer that their reproductive structures were sporocarps.

## ***Morphotype: Fern28***

***Synonymous Morphotypes:*** CS13 (Lyson et al., 2019)

***Nomenclature to be Evaluated:*** *Hydropteris pinnata* Rothwell & Stockey, *Dorfiella knowltonii* (Dorf) Weber

***Age of Rocks:*** Cretaceous

***Morphotype Quality Index:*** Medium

***Reference Specimens:*** DMNH.EPI.23744, DMNH.EPI.51240

***Number of Specimens:*** 14

***Observed Damage Types:*** None

***Percent of Specimens with Herbivory/Pathogen Damage:*** 0%

***Study Sites:*** Denver Basin (Corral Bluffs), Williston Basin

***Diagnostic Characters:*** Penultimate segments pinnate. Ultimate segments 1 - 2 cm in length and obovate in shape, tapering to a point at the base and rounding at the tip. Venation anastomosing.

**Description:** Penultimate segment pinnate, *complete blade structure not observed but likely pinnate*, ultimate segment shape obovate to spatulate, ultimate segment short tapering at the tip, ultimate segment long tapering at the base, ultimate segments symmetrical, *penultimate segment shape not observed*, *penultimate segment tapering at the tip not observed, penultimate segment tapering at the base not observed, penultimate segment symmetry not observed*, *complete frond shape not observed,* venation anastomosing, veins terminate at the margin, *vein termination in lobes/segments and clefts between segments not applicable*, margin entire, *reproductive structures not observed but likely sporocarps (based both on our family assignment and observation of other fossils attributed to this name by previous authors, where these structures were present).*

| ***Fern28 Family Assignment Hypotheses and Reference Genera*** | | |
| --- | --- | --- |
| **Hypothesis Strength** | **Family** | **Reference Genus** |
| Strong | Marsileaceae | *Regellidium; Marsilea* |
| Rejected | Salviniaceae | *Salvinia* |

***Family Assignment Deliberations:*** It has been proposed that *Hydropteris pinnata* Rothwell & Stockey, a name in the literature that matches *Dorfiella knowltonii* (Dorf) Weber, should belong to its own extinct family, Hydropteridaceae (Rothwell and Stockey, 1994). Pryer (1999) argues that *H. pinnata* exhibits combined character states of both Marsileaceae and Salviniaceae and does not completely represent one of these families, suggesting that Hydropteridaceae may be an appropriate classification. The foliage of *H. pinnata* overwhelmingly resembles Marsileaceae rather than Salviniaceae.

Our study did not observe sporocarps or any reproductive structures associated with the foliage of *H. pinnata,* which would be necessary to confirm a Hydropteridaceae affiliation. Our methods also involve attempting to associate each morphotype with an extant family. We do not reject Hydropteridaceae as a hypothesis and instead agree with Pryer’s (1999) findings. However, we cannot confirm the character states of the sporocarps or other reproductive structures due to the lack of preservation in our specimens. Regardless of its exact familial affiliation, we affirm that *H. pinnata* belongs to the order Salviniales.

***Marsileaceae:*** The foliage of *H. pinnata* is strikingly similar to modern Marsileaceae, particularly the genus *Regnellidium* (Plate 25E). In common, ultimate segments have long tapering at the base and short tapering at the tip. This genus also has two paired leaflets/ultimate segments. Marsileaceae commonly exhibits anastomosing venation as observed in *Marsilea* (Plate 25F). Based on the foliage alone, *H. pinnata* likely has an affiliation with Marsileaceae.

***Salviniaceae:*** Salviniaceae (Plate 25D) has been proposed as a closely related family of *H. pinnata* because of their similarity in microsporangiate massulae and megaspore complexes (Pryer 1999). However, this family has minimal morphological similarity with *H. pinnata*, particularly with the foliage, and so we reject this classification. It is highly likely that *H. pinnata* shares a close relationship to Salviniaceae, but certainly does not belong in the family.

## ***Morphotype: Fern29***

***Synonymous Morphotypes:*** NA

***Nomenclature to be Evaluated:*** NA

***Age:*** Paleocene

***Morphotype Quality Index:*** Low

***Reference Specimens:*** DMNH.EPI.22631, DMNH.EPI.22732, DMNH.EPI.22753

***Number of Specimens:*** 7

***Observed Damage Types:*** None

***Percent of Specimens with Herbivory/Pathogen Damage:*** 0%

***Study Sites:*** Williston Basin

***Diagnostic Characters****:* Venation free and forked with veins that do not terminate at the margin. Margin crenate.

***Description:*** *Segment division not observed, complete blade division not observed, ultimate segment shape not observed, ultimate segment tapering at the tip not observed, ultimate segment tapering at the base not observed*, *ultimate segment symmetry not observed, complete frond shape not observed,* venation free and forked, veins do not terminate at the margin, *vein termination in lobes/segments and clefts between segments not applicable*, margin crenate, *reproductive structures not observed.*

| ***Fern29 Family Assignment Hypotheses and Reference Genera*** | | |
| --- | --- | --- |
| **Hypothesis Strength** | **Family** | **Reference Genus** |
| Moderate | Gleicheniaceae | *–* |

***Family Assignment Deliberations:***

***Gleicheniaceae:*** Although this morphotype only has poorly preserved specimens, resulting in a low MQI, it is reasonable to hypothesize that it belongs to Gleicheniaceae. In specimens such as DMNH.EPI.22753, the ultimate segment appears to fork into two further divided segments, which is strongly characteristic of Gleicheniaceae (Plate 29D) (Nauman 2020a). Despite this singular character being nearly diagnostic, a Gleicheniaceae hypothesis is only moderate in strength due to the uncertainty of the forked segment morphology. It is not a verifiable feature because of the preservational state of all specimens. Additionally, Gleicheniaceae often possess arrested buds at the fork of segments. This feature might be present in DMNH.EPI.22732 (Plate 26B), but preservation is too poor to be certain. Ultimately, key characters that would confirm the affinity of Fern29 as Gleicheniaceae are missing either due to preservation or true inconsistency with the defining traits of the family.

## ***Morphotype: Fern30***

***Synonymous Morphotypes:*** CS6 (Lyson et al., 2019)

***Nomenclature to be Evaluated:*** *Anemia elongata* (Newberry) Knowlton

***Age of Rocks:*** Cretaceous and Paleocene

***Morphotype Quality Index:*** Medium

***Reference Specimens:*** YPMPB.061305

***Number of Specimens:*** 10

***Observed Damage Types:*** None

***Percent of Specimens with Herbivory/Pathogen Damage:*** 0%

***Study Sites:*** Denver Basin (Corral Bluffs, West Bijou), Williston Basin, Raton Basin

***Diagnostic Characters****:* Penultimate segments pinnatifid. Ultimate segments elongated linear to oblong. Venation free and forked.

***Description:*** Penultimate segment pinnatifid, *complete blade structure not observed, but likely pinnatifid,* ultimate segment shape linear to oblong, ultimate segment long tapering at the tip, *ultimate segment tapering at the base not applicable*, ultimate segments symmetrical, *penultimate segment shape not observed*, penultimate segment long tapering at the tip, *penultimate segment tapering at the base not observed, penultimate segment symmetry not observed,* *complete frond shape not observed,* venation free and forked, veins terminate at the margin, vein termination in lobes/segments only, margin serrate, *reproductive structures not observed.*

***Discussion:*** Fern30 is potentially the same morphotype as Fern31. If they are the same morphotype, the tip of the frond could be represented by Fern30 and the base represented by Fern31. This hypothesis is similar to the pattern in modern ferns where they can become more segmented towards the base of the frond. Because there are distinguished differences in character states, we have opted to split these into two morphotypes. Regardless of the strength of our proposed familial hypotheses, we argue that these morphotypes are likely closely related.

| ***Fern30 Family Assignment Hypotheses and Reference Genera*** | | |
| --- | --- | --- |
| **Hypothesis Strength** | **Family** | **Reference Genus** |
| Weak | Osmundaceae | *Osmunda* |
| Weak | Blechnaceae | *–* |
| Rejected | Anemiaceae | *Anemia* |
| Rejected | Schizaeaceae | *Schizaea* |

***Family Assignment Deliberations & Anemiaceae:*** The specimens of this morphotype most closely resemble *“Anemia” elongata* (Newberry) Knowlton. The familial assignment of *“A” elongata* has historically had many challenges due to the absence of preserved reproductive structures associated with it. Despite its name, the foliage of *“A.” elongata* does not resemble modern Anemiaceae and therefore does not resemble *Anemia*. Collinson (2001) described *“A.” elongata* as having *Anemia*-like foliage, suggesting Schizaeaceae as its classification. Alternatively, Berry (2023) suggested a Blechnaceae affiliation. Within Anemiaceae, *Anemia phyllitidis* (L.) Sw. (Plate 27E) is most similar to the fossil species. It has the dimorphic fronds, but differs in that it has lanceolate ultimate segments and anastomosing venation. Anemiaceae is typically pinnate, with ultimate segments that are usually much shorter and smaller than observed in *“A.” elongata.* Because the closest modern representative only slightly resembles *“A.” elongata*, it is not reasonable to suspect that it belongs in *Anemia*, and therefore Anemiaceae. The affiliation of *“Anemia” elongata* likely cannot be fully resolved without the preservation of reproductive structures, but we have evaluated suggestions made in the literature and have proposed Osmundaceae as an additional hypothesis.

***Osmundaceae:*** Due to the lack of observed reproductive structures, we suggest that*“Anemia” elongata* likely has strongly dimorphic fronds, but this hypothesis has not been directly observed. This could provide an explanation as to why only the sterile foliage of this morphotype has been found. Because Osmundaceae is known for its strongly dimorphic fronds, we have suggested it as a hypothesis. The description of modern Osmundaceae is consistent with *“A.” elongata* in that members of this family can be pinnatifid, have free forked veins, and sori are absent on leaf tissue (Whetstone and Atkinson, 2020). No features of modern Osmundaceae contradict what is observed in *“A.” elongata*. *Osmunda vachellii* Hook. (Plate 27B) has the similar features to *“A.” elongata* such as the linear ultimate segments and free forked veins, though it differs in that it is pinnate. Frond dimorphism is present in a diversity of ferns, so our hypothesis that *A. elongata* is dimorphic remains a weak hypothesis that cannot be confirmed without the observation of reproductive structures.

***Blechnaceae:*** A Blechnaceae affiliation of *“Anemia” elongata* was suggested by Berry (2023). Members of Blechnaceae can be dimorphic often with herbaceous fertile foliage. This idea could indicate that the fertile foliage of *“A.” elongata* may appear morphologically different in many character states, ultimately leading to identification of two separate morphotypes. For example, some Blechnaceae species have sterile foliage with free venation and fertile foliage with anastomosing venation (Cranfill, 2020a). This is certainly a possibility as no character states of the *“A.” elongata* sterile foliage contradict with the modern description Blechnaceae. We have determined this to be a weak hypothesis because frond dimorphism in *“A.” elongata* has not been confirmed.

***Schizaeaceae:*** Several publications have affiliated *“Anemia” elongata* to the family Schizaeaceae (Brown, 1962; Collinson, 2001; Knowlton, 1930; Manchester, 2014). Members of Schizaeaceae can have dimorphic fronds, consistent with our hypothesis for “*A”* *elongata.* However, none of the ferns in the two modern genera of Schizaeaceae (*Actinostachys* and *Schizaea*) remotely resemble *“A.” elongata* in morphology*.* Many species of *Schizaea* (Plate 27C) have flabellate fronds, with ultimate and penultimate segments exhibiting dramatically different segment structures. There are no strong morphological similarities that can correlate this family to *“A. elongata”* and it is a rejected hypothesis.

## ***Morphotype: Fern31***

***Synonymous Morphotypes:*** NA

***Nomenclature to be Evaluated:*** *Diplazium crossii* (Knowlton) Knowlton

***Age of Rocks:*** Cretaceous and Paleocene

***Morphotype Quality Index:*** Medium

***Reference Specimens:*** DMNH.EPI.51023, VPR.013, SS.046, CB.037

***Number of Specimens:*** 156

***Observed Damage Types:*** None

***Percent of Specimens with Herbivory/Pathogen Damage:*** 0%

***Study Sites:*** Denver Basin (Corral Bluffs, West Bijou, Plum Creek Parkway), Williston Basin, Raton Basin

***Diagnostic Characters:*** Tertiary ultimate segments pinnate-pinnatifid. Ultimate segments elliptic. Venation free and forked and diverges from the axial vein of the penultimate segment as parallel venation in the ultimate segment. No axial vein in the ultimate segment. Margin serrate.

***Description:*** Tertiary ultimate segment pinnate-pinnatifid, *complete blade structure not observed*, ultimate segment shape elliptic, ultimate segment long tapering at the tip, *ultimate segment tapering at the base not applicable*, ultimate segments symmetrical, penultimate segment shape lanceolate to oblanceolate, penultimate segment long tapering at the tip, penultimate segment long tapering at the base, penultimate segments symmetrical, *tertiary ultimate segment shape not observed, tertiary ultimate segment tapering at the tip not observed, tertiary ultimate segment tapering at the base not observed, complete frond shape not observed,* venation free and forked, veins terminate at the margin, vein termination in lobes/segments and clefts between segments, margin serrate, *reproductive structures not observed.*

| ***Fern31 Family Assignment Hypotheses and Reference Genera*** | | |
| --- | --- | --- |
| **Hypothesis Strength** | **Family** | **Reference Genus** |
| Weak | Osmundaceae | *Osmunda* |
| Weak | Blechnaceae | *–* |
| Rejected | Athyriaceae | *Diplazium* |
| Rejected | Lonchitidaceae | *Lonchitis* |
| Rejected | Dryopteridaceae | *Rumorha* |

***Family Assignment Deliberations & Athyriaceae:*** The morphotype was previously identified as *Diplazium crossii* (Knowlton) Knowlton and assigned to the family Polypodiaceae (Knowlton, 1930). In modern classifications, *Diplazium* belongs to the family Athyriaceae (Pteridophyte Phylogeny Group, 2016). Because the molecular age of Polypodiaceae postdates the Cretaceous-Paleogene boundary (Pelosi et al., 2022), we have not investigated the family as a valid hypothesis for *“D.” crossii.*

Similar to *“Anemia” elongata*, no fertile material has been associated with*“D.” crossii*, despite its abundance. As a result, we hypothesize that *“D.” crossii* has strongly dimorphic fronds that have not been preserved or that have not been associated with the sterile foliage. *“Diplazium” crossii* should be rejected as the name for this morphotype because modern *Diplazium* and the Athryiaceae family tend to have monomorphic sterile and fertile fronds. With the abundance of specimens found for this morphotype, it is reasonable to assume that reproductive structures are not preserved on fronds that are nearly identical to the sterile fronds. From this, Athyriaceae, and therefore, the *Diplazium* hypothesis, should be rejected.

The dimorphic frond hypothesis for this morphotype provides a challenge in assigning it to a family. Further, we have observed no modern species of ferns that strongly resemble *“D.” crossii*, particularly considering its venation pattern. The ultimate segments of this morphotype lack an axial vein and instead have near-parallel, free forked veins running to the margin.

Additionally, although not completely confirmed, there is preserved material showing an axis bearing sori that may be associated with “*D.*” *crossii* (Plate 28B). Confirmation of the affiliation between this potentially fertile fern material and the sterile leafy foliage would aid in resolving familial taxonomic placement and support our dimorphic frond hypothesis. However, due to the uncertainty regarding the association between the fertile and sterile material, we have excluded the fertile structure from our descriptions and considerations of familial hypotheses.

***Osmundaceae:*** The weak hypothesis of Osmundaceae comes from the similarity in ultimate and penultimate segment shape to modern species like *Osmunda cinnamomea* L. (Plate 29A). Also in common, the ultimate segments transition into lobes moving toward the tip of the frond. The venation of this species is free and forked, but an axial vein is present in ultimate segments, different from *“D.” crossii.* Additionally, in congruence with our *“D.” crossii* dimorphic frond hypothesis, Osmundaceae is known for its strongly dimorphic fronds. Because the state of the fertile material has not been confirmed, Osmundaceae is a weak hypothesis.

***Blechnaceae:*** This morphotype may be associated with Blechnaceae due to a similar rationale stated for *“Anemia” elongata* and in Berry (2023): that both may have strongly dimorphic fronds, with fertile fronds having a dramatically different overall appearance from the sterile fronds. The fronds/penultimate segments of *Blechnum* (Plate 29B) often exhibit long tapering at the base, similar to the penultimate segments of *“D.” crossii*. It must be noted, though, that *“D.” crossii* is divided at least three times into a tertiary ultimate segment, and the penultimate segment does not represent the entire frond. Based on this evidence, Blechnaceae as a hypothesis only has weak support.

***Lonchitidaceae:*** This family was suggested in the survey we conducted. Modern *Lonchitis hirsuta* L. (Plate 29C) has ultimate and penultimate segment shapes that resemble *“Diplazium” crossii.* However, sori in this species rest between the clefts of the ultimate segments/lobes on the herbaceous foliage and neither of the estimated two modern species in this family (Pteridophyte Phylogeny Group, 2016) have a strong resemblance to *“D.” crossii*. As a result, this hypothesis is rejected.

***Dryopteridaceae:*** Modern *Rumohra* (Plate 29D) is similar to *“Diplazium” crossii* in the shape of the ultimate and penultimate segments. This genus also has what appears to be the most similar venation to the distinct parallel veins in ultimate segments of *“D.” crossii.* However, *Rumohra* has monomorphic fronds with sori on herbaceous foliage. Other members of Dryopteridaceae can have dimorphic fronds (Smith, 2020a), but none were observed that closely resemble the segment shapes and venation of *“D.” crossii,* leading us to reject this family as a hypothesis.

## ***Morphotype: Fern32***

***Synonymous Morphotypes:*** NA

***Nomenclature to be Evaluated:*** NA

***Age of Rocks:*** Cretaceous

***Morphotype Quality Index:*** Medium

***Reference Specimens:*** YPMPB.061303

***Number of Specimens:*** 1

***Observed Damage Types:*** Surface feeding DT031; pathogen/environment DT058.

***Percent of Specimens with Herbivory/Pathogen Damage:*** 100%

***Study Sites:*** Denver Basin

***Diagnostic Characters:*** Tertiary ultimate segments bipinnate. Segments become more divided toward the base of the frond. Ultimate segments oblong to elliptic. Venation free and forked. Margin entire.

***Description:*** Tertiary ultimate segment bipinnate, *complete blade structure not observed but likely bipinnate,* ultimate segment shape oblong to elliptic, ultimate segment short tapering at the tip, ultimate segment short tapering at the base, ultimate segments symmetrical, *penultimate segment shape not observed, penultimate segment tapering at the tip not observed,* penultimate segment long tapering at the base, *penultimate segment symmetry not observed,* *tertiary ultimate segment shape not observed,* tertiary ultimate segment long tapering at the tip, *tertiary ultimate segment tapering at the base not observed*, *complete frond shape not observed,* venation free and forked, veins terminate at the margin, *vein termination in lobes/segments and clefts between segments not applicable*, margin entire, *reproductive structures not observed.*

| ***Fern32 Family Assignment Hypotheses and Reference Genera*** | | |
| --- | --- | --- |
| **Hypothesis Strength** | **Family** | **Reference Genus** |
| Weak | Osmundaceae | *Osmunda* |
| Rejected | Pteridaceae | *Pteris* |

***Family Assignment Deliberations:***

***Osmundaceae:*** Fern32 has a close morphological similarity to the modern *Osmunda regalis* (Plate 30B). Both transition from a pinnate structure towards the tip of the frond and become more divided, bipinnate even, towards the base of the frond. Additionally, they have free forked venation along with elliptic ultimate segment shapes. Only one specimen of Fern32 was observed in this study and only consists of a singular infertile portion of a frond. There are no prominent differences observed between Fern32 and *O. regalis*, but no reproductive structures have been observed, causing Osmundaceae to be a weak hypothesis.

***Pteridaceae:*** *Pteris* was suggested as a possible genus in the survey we conducted. However, none of the observed modern species for *Pteris* or Pteridaceae are notably similar in appearance. Many of these species have pinnatifid penultimate segments and anastomosing venation. We have rejected Pteridaceae as a hypothesis.

## ***Morphotype: Fern33***

***Synonymous Morphotypes:*** CS18 (Lyson et al., 2019)

***Nomenclature to be Evaluated:*** *Equisetum* sp.

***Age of Rocks:*** Cretaceous and Paleocene

***Morphotype Quality Index:*** High

***Reference Specimens:*** DMNH.EPI.21842, DMNH.EPI.24872, DMNH.EPI.22099, UWBMPB.99694

***Number of Specimens:*** 23

***Observed Damage Types:*** None

***Percent of Specimens with Herbivory/Pathogen Damage:*** 0%

***Study Sites:*** Denver Basin (Corral Bluffs, West Bijou), Williston Basin

***Diagnostic Characters:*** Ridged stems with node-internode structure and whorled branches. Leaves minute and fused into sheaths at nodes.

***Description:*** Likely separate sterile and fertile stalks. Sterile stems ridged with node-internode structure, whorls of branches, and leaves, if present, minute, tooth-like, and fused into sheaths at nodes, storage tubers globular and on rhizomes, *reproductive structures not observed but likely strobili.*

| ***Fern33 Family Assignment Hypotheses and Reference Genera*** | | |
| --- | --- | --- |
| **Hypothesis Strength** | **Family** | **Reference Genus** |
| Strong | Equisetaceae | *Equisetum* |

***Family Assignment Deliberations:***

***Equisetaceae:*** This morphotype has ridged stems with a node and internode structure, whorls of branches, and stem sheaths with teeth, which are actually highly reduced leaves and which are characteristic of Equisetaceae. The traits are consistent across specimens, and we can confidently state that these belong to the same family. What is unknown is if all specimens belong to the same species because sheaths, stems, leaves, and storage tubers on rhizomes are the only characters preserved; no strobili were observed.

**Plate 1** Fern01 *Allantodiopsis erosa*; (A) fertile ultimate segment, DMNH.EPI.40382; (B) complete sterile ultimate segment, DMNH.EPI.51033; (C) pinnate division of the penultimate segment, DMNH.EPI.51320; (D) serrated margin of the ultimate segment, DMNH.EPI.40385; (E) elongated sori embedded in the lamina with an indusium originating on both sides of the sorus, DMNH.EPI.40380.

**Plate 2** Fern01 *Allantodiopsis erosa* modern comparisons; (A) Aspleniaceae, *Asplenium feei* Kunze ex Fée; (B) Athyriaceae, *Diplazium sanctae-rosae* Christ.; (C) Osmundaceae, *Osmunda regalis* L.; (D) Diplaziopsidaceae, *Hemidictyum marginatum* (L.) C.Presl; (E) Polypodiaceae, *Pleopeltis astrolepis* (Liebm.) E.Fourn.; (F) Thelypteridaceae, *Goniopteris falcatum*. (Modern fern images by Robbin Moran via [plantsystematics.org](http://plantsystematics.org/)).

**Plate 3** Fern02 *Woodwardia arctica*; (A) tertiary ultimate segment, box outlines ultimate segment, DMNH.EPI.51254; (B) partial segment with anastomosing venation, DMNH.EPI.50996; (C) fertile penultimate segment with linear sporangia parallel to axial veins, DMNH.EPI.22660; (D) fertile segment with linear sporangia parallel to axial veins, UWBMPB.99706; (E) linear sporangia, DMNH.EPI.22660.

**Plate 4** Fern02 *Woodwardia arctica* modern comparisons; (A) Blechnaceae, *Woodwardia radicans* L.; (B) Blechnaceae, *Blechnum occidentale* L.; (C) Onocleaceae, *Onoclea sensibilis* L.; (D) Pteridaceae, *Pteris altissima* Poir*.* (Modern fern images by Robbin Moran via [plantsystematics.org](http://plantsystematics.org/)).

**Plate 5** Fern03 and modern comparisons; (A) tertiary ultimate segment, DMNH.EPI.21114; (B) tertiary ultimate segment with arrows pointing to possible pathogen damage or irregular clasts (note that there are many), DMNH.EPI.19968; (C) Blechnaceae, *Woodwardia radicans*; (D) Onocleaceae, *Onoclea sensibilis*. (Modern fern images by Robbin Moran via [plantsystematics.org](http://plantsystematics.org/)).

**Plate 6** Fern04 *Salpichlaena anceps* and modern comparisons; (A) ultimate segment with linear sporangia parallel to ultimate segment axial vein, box outlines portion of linear sporangia, DMNH.EPI.51184; (B) penultimate segment, DMNH.EPI.51248; (C) penultimate segment, DMNH.EPI.51182; (D) Blechnaceae, *Salpichlaena papyrus* G.G.Cárdenas, Tuomisto & Lehtonen; (E) Blechnaceae, *Parablechnum cordatum* (Desv.) Gasper & Salino. (Modern fern images by Robbin Moran via [plantsystematics.org](http://plantsystematics.org/)).

**Plate 7** Fern05 *Dennstaedtia crossiana*, Fern06, and modern comparisons; (A) fertile quaternary ultimate segment (Fern05), box outlines a penultimate segment, DMNH.EPI.52058; (B) tertiary ultimate segments (Fern06), DMNH.EPI.52098; (C) tertiary ultimate segments, both morphotypes on the same block: (Fern05 (left), Fern06 (right)), box outlines penultimate segment, DMNH.EPI.52059; (D) marginal sori with a likely cup-shaped indusium (Fern05), DMNH.EPI.52074; (E) Dennstaedtiaceae, *Dennstaedtia cicutaria* (Sw.) T.Moore (Fern05, Fern06); (F) Dryopteridaceae, *Dryopteris patula* (Sw.) Underw. (Fern05). (Modern fern images by Robbin Moran via [plantsystematics.org](http://plantsystematics.org/)). (Plate is Figure 9 in manuscript).

**Plate 8** Fern07 and modern fern comparisons; (A) quaternary ultimate segment, DMNH.EPI.51242; (B) tertiary ultimate segment, DMNH.EPI.51189; (C) Dryopteridaceae, *Dryopteris marginalis* (L.) A.Gray; (D) penultimate segments with venation free and forked, DMNH.EPI.51191; (E) Davalliaceae; *Davallia tyermanii* T.Moore; (F) Dennstaedtiaceae, *Dennstaedtia obtusifolia* (Willd.) T.Moore; (G) Anemiaceae, *Anemia irwinii* Mickel. (Modern fern images by Robbin Moran via [plantsystematics.org](http://plantsystematics.org/)).

**Plate 9** Fern08 and modern comparisons; (A) tertiary ultimate segment, DMNH.EPI.23742; (B) penultimate segment, DMNH.EPI.34378; (C) Thelypteridaceae, *Goniopteris curta* (Christ) A.R.Sm.; (D) Athyriaceae, *Diplazium striatastrum* Lellinger; (E) Osmundaceae, *Osmunda claytoniana* L. (Modern fern images by Robbin Moran via [plantsystematics.org](http://plantsystematics.org/)).

**Plate 10** Fern09 and modern comparisons; (A) complete fertile penultimate segments, DMNH.EPI.58775; (B) round sori along lateral veins, DMNH.EPI.58775; (C) Thelypteridaceae, *Goniopteris curta*; (D) Cyatheaceae, *Cyathea bipinnatifida* Copel. (Modern fern images by Robbin Moran via [plantsystematics.org](http://plantsystematics.org/)).

**Plate 11** Fern10 and modern comparisons; (A) fertile penultimate segment with small, round sori, DMNH.EPI.40369; (B) sterile penultimate segment, DMNH.EPI.40370; (C) Thelypteridaceae, *Goniopteris curta*; (D) Thelypteridaceae, *Thelypteris palustris* Schott; (E) Dryopteridaceae, *Dryopteris goldiana* Dowell; (F) Nephrolepidaceae, *Nephrolepis pectinata* (Willd.) Baker. (Modern fern images by Robbin Moran via [plantsystematics.org](http://plantsystematics.org/)).

**Plate 12** Fern11 and modern comparisons; (A) tertiary ultimate segment, DMNH.EPI.40376; (B) tertiary ultimate segment, DMNH.EPI.51024; (C) Thelypteridaceae, *Steiropteris decussata* (L.) A.R.Sm.; (D) Athyriaceae, *Diplazium lindbergii* (Mett.) Christ; (E) Gleicheniaceae, *Diplopterygium bancroftii* (Hook.) A.R.Sm.; (F) Osmundaceae, *Osmunda claytoniana*. (Modern fern images by Robbin Moran via [plantsystematics.org](http://plantsystematics.org/)).

**Plate 13** Fern12 *Saccoloma gardneri* and modern comparisons; (A) fertile ultimate segment with venation free and forked, DMNH.EPI.51185; (B) round sori arranged linearly along margin with cup-shaped indusia; fertile ultimate segment, DMNH.EPI.51186; (C) Saccolomataceae, *Saccoloma elegans* Kaulf.; (D) Dennstaedtiaceae, *Dennstaedtia coronata* (Sodiro) C.Chr.; (E) Pteridaceae, *Pteris nipponica* Shieh; (F) DMNH.EPI.51186. (Modern fern images by Robbin Moran via [plantsystematics.org](http://plantsystematics.org/)).

**Plate 14** Fern13 and modern comparisons; (A) fertile ultimate segment with anastomosing venation, DMNH.EPI.24389; (B) sterile ultimate segment, DMNH.EPI.23896; (C) Saccolomataceae, *Saccoloma elegans*; (D) round sori along the margin arranged linearly with cup-like indusia, DMNH.EPI.24389; (E) Dennstaedtiaceae, *Dennstaedtia coronata*; (F) Pteridaceae, *Pteris altissima*. (Modern fern images by Robbin Moran via [plantsystematics.org](http://plantsystematics.org/)).

**Plate 15** Fern14 and modern comparisons; (A) penultimate segments, box outlines penultimate segment, arrow points to ultimate segment, DMNH.EPI.51031; (B) Hymenophyllaceae, Trichromanoideae, *Didymoglossum godmanii* (Hook. ex Baker) Ebihara & Dubuisson; (C) Hymenophyllaceae, Hymenophylloideae, *Hymenophyllum fucoides* (Sw.) Sw. (Modern fern images by Robbin Moran via [plantsystematics.org](http://plantsystematics.org/)).

**Plate 16** Fern15 and modern comparisons; (A) quaternary ultimate segment, DMNH.EPI.50995; (B) tertiary ultimate segment with arrows pointing to cone-shaped sori and funnel-shaped indusia, DMNH.EPI.49735; (C) Hymenophyllaceae, Trichomanoideae, *Crepidomanes pyxidiferum* (L.) Dubuisson & Ebihara; (D) Hymenophyllaceae, Hymenophylloideae, *Hymenophyllum tunbridgense* (Labill.) Hook.f.; (E) Pteridaceae, *Onychium japonicum* (Thunb.) Kunze. (Modern fern images by Robbin Moran via [plantsystematics.org](http://plantsystematics.org/)).

**Plate 17** Fern16, Fern17, and modern comparisons; (A) penultimate segment (Fern16), DMNH.EPI.51244; (B) ultimate segment (Fern17), DMNH.EPI.51246; (C) Hymenophyllaceae, *Didymoglossum membranaceum* (L.) Vareschi (Fern16, Fern17); (D) Schizaeaceae, *Schizaea elegans* (Vahl) Sw. (Fern17); (E) Pteridaceae, *Adiantum andicola* Liebm. (Fern17)*.* (Modern fern images by Robbin Moran via [plantsystematics.org](http://plantsystematics.org/)).

floc

**Plate 18** Fern18 *Dryopteris lakesii* and modern comparisons; (A) fertile penultimate segment, DMNH.EPI.40407; (B) fertile penultimate segment on a tertiary ultimate segment, box outlines fertile penultimate segment, DMNH.EPI.51331; (C) sterile tertiary ultimate segment, DMNH.EPI.51250; (D) round sorus with a true indusium, DMNH.EPI.40407; (E) Cyatheaceae, *Cyathea multiflora* Sm.; (F) Dryopteridaceae, *Dryopteris campyloptera* Clarkson, (G) Tectariaceae, *Tectaria mexicana* (Fée) C.V.Morton. (Modern fern images by Robbin Moran via [plantsystematics.org](http://plantsystematics.org/)).

**Plate 19** Fern19, Fern21, and modern comparisons; (A) ultimate segment (Fern21), DMNH.EPI.23739; (B) ultimate segment (Fern21), DMNH.EPI.34379; (C) tertiary ultimate segment, (Fern19), DMNH.EPI.51030; (D) close-up of penultimate segment, arrows point to soral indentations, DMNH.EPI.51030; (E) Dryopteridaceae, *Cyrtomium fortunei* [J.Sm](http://j.sm/).; (F) Athyriaceae, *Diplazium bombonasae* Rosenst. (Fern19); (G) Dryopteridaceae, *Dryopteris polylepis* (Franch. & Sav.) C.Chr. (Fern21); (H) Cyatheaceae, *Cyathea bipinnatifida* (Fern21). (Modern fern images by Robbin Moran via [plantsystematics.org](http://plantsystematics.org/)).

**Plate 20** Fern20 *Dryopteris integra* and modern comparisons; (A) fertile penultimate segment with round sori centered between ultimate segment axial vein and margin, DMNH.EPI.21785; (B) sori with true, round indusia, DMNH.EPI.21785; (C) fertile penultimate segment, DMNH.EPI.21790; (D) sori with true, round indusia, visible round sporangia, DMNH.EPI.21785; (E) Dryopteridaceae, *Dryopteris wallichiana* (Spreng.) Hyl. (image by Robbin Moran via [plantsystematics.org](http://plantsystematics.org/)).

**Plate 21** Fern22 and modern comparisons; (A) quaternary ultimate segment with asymmetrical? penultimate segments, YPMPB.061361; (B) tertiary ultimate segment, YPMPB.041985; (C) Dryopteridaceae, *Dryopteris patula*; (D) Saccolomataceae, *Saccoloma inaequale* (Kunze) Mett.; (E) Athyriaceae, *Diplazium pominulum*; (F) Dennstaedtiaceae, *Hypolepis hostilis* (Kunze) C.Presl. (Modern fern images by Robbin Moran via [plantsystematics.org](http://plantsystematics.org/)).

**Plate 22** Fern23 and modern comparisons; (A) penultimate segment with serrate margin and venation free and forked, DMNH.EPI.51954; (B) tertiary ultimate segment, DMNH.EPI.51952; (C) Dryopteridaceae *Dryopteris cristata* Watts; (D) Athyriaceae, *Athyrium vidalii* (Franch. & Sav.) Nakai. (Modern fern images by Robbin Moran via [plantsystematics.org](http://plantsystematics.org/)).

**Plate 23** Fern24, Fern25, and modern comparisons; (A) quaternary ultimate segment with tripinnate division and likely a triangular-pentagonal shape (Fern24), box outlines penultimate segment, DMNH.EPI.23839; (B) Pteridaceae, *Pentagramma maxonii* (Weath.) Schuettp. & Windham (Fern24); (C) Pteridaceae, *Notholaena candida* (M.Martens & Galeotti) Hook (Fern24); (D) penultimate segments (Fern25), YPMPB.066250; E) Pteridaceae, *Pteris pungens* Krug (Fern25); (F) Osmundaceae, *Osmunda regalis* (Fern25)*.* (Modern fern images by Robbin Moran via [plantsystematics.org](http://plantsystematics.org/)).

**Plate 24** Fern26 and modern comparisons; (A) tertiary ultimate segment, DMNH.EPI.40371; (B) close up of segments, box outlines articulation? to the axial vein, DMNH.EPI.40371; (C) penultimate segments, DMNH.EPI.40372; (D) Nephrolepidaceae, *Nephrolepis undulata* (Afzel. ex Sw.) J.Sm., box outlines ultimate segment with articulation to penultimate segment axial vein; (E) Pteridaceae, *Pteris quadriaurita* Retz. (Modern fern images by Robbin Moran via [plantsystematics.org](http://plantsystematics.org/)).

**Plate 25** Fern27 *Salvinia* sp., Fern28 *Hydropteris pinnata*, and modern comparisons; (A) paired blades/ultimate segments (Fern27), DMNH.EPI.23743; (B) penultimate segments (Fern28), DMNH.EPI.51240; (C) penultimate segment (Fern28), DMNH.EPI.23744; (D) Salviniaceae, *Salvinia minima* Baker (Fern27, Fern28); (E) Marsileaceae, *Regnellidium diphyllum* Lindm. (Fern28); (F) Marsileaceae, *Marsilea mutica* Mett. (Fern28). (Modern fern images by Robbin Moran via [plantsystematics.org](http://plantsystematics.org/)). (Plate is Figure 11 in manuscript).

**Plate 26** Fern29 and modern comparisons; (A) forked ultimate segments, DMNH.EPI.22753; (B) forked ultimate segments, box outlines possible arrested bud, DMNH.EPI.22732; (C) crenate margin and free forked veins not terminating at the margin, DMNH.EPI.22631; (D) Gleicheniaceae, *Sticherus revolutus* (Kunth) Ching (image by Robbin Moran via [plantsystematics.org](http://plantsystematics.org/)).

**Plate 27** Fern30 *“Anemia” elongata* and modern comparisons; (A) penultimate segment, YPMPB.061305; (B) Osmundaceae, *Osmunda vachellii* Hook.; (C) Schizaeaceae, *Schizaea elegans*; (D) Blechnaceae, *Parablechnum schiedeanum* (Schltdl. ex C.Presl) Gasper & Salino; (E) Anemiaceae, *Anemia phyllitidis* (L.) Sw. (Modern fern images by Robbin Moran via [plantsystematics.org](http://plantsystematics.org/)).

**Plate 28** Fern31 *“Diplazium” crossii*; (A) penultimate segment, DMNH.EPI.51023, (B) penultimate segment with near-parallel venation free and forked and lacking an axial vein on ultimate segments, VPR.013; (C) axis bearing sori? with possible association to Fern31, SS.025; (D) penultimate segment long tapering at the base, SS.046; (E) tertiary ultimate segment, CB.037. (Plate is Figure 9 in manuscript).

**Plate 29** Fern31 *“Diplazium” crossii* modern comparisons; (A) Osmundaceae, *Osmunda cinnamomea* L.; (B) Blechnaceae, *Blechnum polypodioides* (Sw.) Kuhn; (C) Lonchitidaceae, *Lonchitis hirsuta* L.; (D) Dryopteridaceae, *Rumohra adiantiformis* (G.Forst.) Ching; (E) Athyriaceae, *Diplazium squamigerum* (Mett.) Christ. (Modern fern images by Robbin Moran via [plantsystematics.org](http://plantsystematics.org/)). (Plate is Figure 10 in manuscript).

**Plate 30** Fern32 and modern comparisons; (A) tertiary ultimate segment, YPMPB.061303; (B) Osmundaceae, *Osmunda regalis*; (C) Pteridaceae, *Pteris livida* Mett. (Modern fern images by Robbin Moran via [plantsystematics.org](http://plantsystematics.org/)).

**Plate 31** Fern33 *Equisetum* sp. and modern comparisons; (A) stalk with whorled branches (leaves in *Equisetum* are highly reduced), DMNH.EPI.21842; (B) stem with node-internode structure, DMNH.EPI.24872; (C) sheath of leaves surrounding the stem at a node, UWBMPB.99694; (D) globular storage tubers on rhizome, DMNH.EPI.22099; (E) Equisetaceae, *Equisetum bogotense* Kunth; (F) Equisetaceae, *Equisetum arvense* L.; (G) Equisetaceae, *Equisetum telmateia* Ehrh. (Modern fern images by Robbin Moran via [plantsystematics.org](http://plantsystematics.org/)).
