# Supplementary material for: Application of a novel schema for describing fossil fern foliage and investigating taxonomic and morphological change across the Cretaceous–Paleogene boundary in western North America
Source: Am J Bot. 2026 Jul 30;113(8):e70243. doi: 10.1002/ajb2.70243 (PMC13496116; doi:10.1002/ajb2.70243)
Supplement: Supplementary file 2 [file AJB2-113-e70243-s002.docx]

**Appendix S2.** Descriptive schema for fossilized ferns

## ***Description Schema for Fossilized Ferns***

The complete description formula is designed to describe nearly all observable characteristics of the fossilized fern frond or segments of the frond and is intended to be expanded upon. The system ensures consistency in describing morphotypes and draws attention to traits that are characteristic of certain modern taxa. It is designed to acknowledge character states that are not observed or not applicable, preventing misinterpretations or incomplete descriptions.

This method accommodates for frond dimorphism. Sterile and fertile fronds can be described separately, except when fertile fronds lack leaf tissue or fertile fronds/segments are sporophores. In these cases, the fertile fronds are described through these characters in the complete description formula: reproductive structure type/shape, reproductive structure placement, and presence of indusia/indusium type. Monomorphic fronds (those with nearly identical sterile and fertile fronds) only require a single description.

## ***Fern Segment Terminology***

In modern fern descriptions, terminology such as blade, pinna, pinnule, and pinnulet are commonly used to describe the segments of fronds. Accurately applying these terms requires the highest order division to be known, meaning the entire frond must be preserved. However, most fern fossils are fragmentary and consist of segments rather than complete fronds. If the entire frond is not preserved, the complete blade division is unknown, and the traditional segment terminology (i.e., pinna, pinnule, pinnulet) cannot be applied consistently, quickly becomes speculative, and causes ambiguity in descriptions.

To address this issue, we propose a new system for describing fossilized fern fronds and segments of fronds, starting with the smallest order of division first. The key terms used for segments in this system, from smallest to largest, are as follows: ultimate segment, penultimate segment, tertiary ultimate segment, quaternary ultimate segment, and so forth (Figure S2.1; Table S2.1). These terms are additionally interchangeable with first-order ultimate segment, second-order ultimate segment, third-order ultimate segment, fourth-order ultimate segment, and so forth. This framework is based on Tryon’s (1960) fern frond terminology, which defines ultimate and penultimate segments, and extends this terminology to include higher-order division segments. This approach allows for consistent terminology when different orders of division are preserved on different specimens within the same morphotype. It ensures that the same terminology is used for each segment. Additionally, for this study, we have opted to refer to any structures that may be perceived as lobes, instead as ultimate segments. This terminology was chosen because lobes often transition into segments toward the base of fern fronds and allows for consistency and simplicity in descriptions.

**Figure S2.1.** Segment terminology. The smallest order segments are designated as ultimate segments, followed sequentially by penultimate segments, tertiary ultimate segments, and quaternary ultimate segments.

| **Table S2.1 Key Terminology for Describing Ferns** | |
| --- | --- |
| **Term** | **Definition** |
| Blade | Portion of the frond that contains herbaceous tissue. |
| Frond | The entire leaf including the blade and stipe. |
| Axial Vein | The central vein on a segment. |
| Segment | A portion of a divided fern frond. |
| Ultimate Segment | The smallest, most divided segment on the frond that does not have smaller segments. |
| Penultimate Segment | The second to last divided segment that bears ultimate segments. |
| Tertiary Ultimate Segment | The third to last divided segment that bears penultimate and ultimate segments. |
| Quaternary Ultimate Segment | The fourth to last divided segment that bears tertiary ultimate, penultimate, and ultimate segments. |
| Pinna | The largest order segment of a frond. |
| Pinnule | The second largest order segment of a frond. |
| Pinnulet | The third largest order segment of a frond. |
| Cleft | The space between the leaf tissue of lobes or ultimate segments of a pinnatifid penultimate segment. |
| Apex | Tip of the frond or segment. |
| Base | Bottom of the frond or segment. |
| Sori/Sorus | Clusters of sporangia containing spores. |
| Sporangia/  Sporangium | Structures that release spores. |
| Sporophore | Sporangia and spores are in reduced, modified blades such as in Ophioglossaceae. |
| Sporocarp | Modified structures derived from leaves or pinnae containing modified sori that produce both megaspores and microspores, present in aquatic ferns. |
| Indusia/Indusium | Specialized protective tissue that covers sori. |
| Acroscopic | Toward the tip of the frond. |
| Basiscopic | Toward the base of the frond. |
| Dimorphic Fronds | Distinct fertile fronds and sterile fronds that differ in morphology and function. |
| Monomorphic Fronds | Fronds have a uniform morphology, with little to no distinct morphological differences between fertile fronds and sterile fronds. |
| Sterile Frond | A frond with the primary purpose of photosynthesis and lacks reproductive structures. |
| Fertile Frond | A frond that bears reproductive structures and may or may not have photosynthetic tissue. |

## ***Description Schema Guidelines***

Each character and their character states are listed in the complete description formula and the complete description schema. Definitions of the characters and character states are provided with the complete description schema and supplemented with a table of additional key terminology. All character states in the schema should be recorded verbatim and incorporated directly into the complete description formula. Generally, the character state will be explicitly recorded as ‘not observed’ or ‘not applicable’. However, the following exceptions to this rule are outlined with modifications or additions that may be necessary:

***1) Unlisted Character States:*** The complete description schema lists a broad overview of character states that should apply to most ferns. There may be instances where a character state is not listed or predefined in the schema. In these cases, the character state should be recorded, following the general formatting guidelines of the schema.

*Example:* a 4-pinnate blade structure is observed, but the schema does not explicitly list this character state.

*Character:* Complete Blade Division

*Character State:* Blade Structure 4-Pinnate

***2) Relevant Unlisted Characters:*** In some cases, a character is not included in the standard schema but is still relevant for classification. These characters should only be added if they provide meaningful diagnostic information and contribute to the identification of a morphotype.

*Example 1:* An observed tertiary ultimate segment is asymmetrical across the quaternary ultimate segment axial vein.

*Character:* Tertiary Ultimate Segment Symmetry

*Character State:* Tertiary Ultimate Segment Asymmetrical

**Note that this character is not included in the standard description formula because it is typically not relevant. In this example, it is included because segment asymmetry aids in classification diagnosis. Conversely, symmetrical tertiary ultimate segments should not be explicitly stated as the alternative character state because it typically does not provide useful diagnostic information.*

*Example 2:* An observed penultimate segment has a paripinnate structure.

*Character:* Paripinnate or Imparipinnate Penulitmate Segment

*Character State:* Paripinnate Penultimate Segment

**Paripinnate structure is not observed in modern ferns, but it is possible that it exists in the fossil record. This is not in the schema because it is typically not relevant. But, in the instance that it is, the character state should be included.*

***3) Character State Variation:*** If there is variation in a character state of a morphotype, all character states observed should be recorded.

*Example:* The ultimate segment shapes vary from ovate to elliptic.

*Character:* Ultimate Segment Shape

*Character State:* Ultimate Segment Shape Ovate to Elliptic

***4) Unobserved Segments:*** If no evidence of a segment is observed, the associated segment characters and character states do not need to explicitly state that they are not observed.

*Example:* A bipinnate tertiary ultimate segment is observed as the character state for preserved segment division. Characters associated with the quaternary ultimate segment do not need to be explicitly stated because it is implied that they are not observed.

*Characters that do not need to be explicitly stated:* Quaternary Ultimate Segment Shape, Quaternary Ultimate Segment Tapering at the Tip, Quaternary Ultimate Segment Tapering at the Base

**Because the tertiary ultimate segment was mentioned in the preserved segment division, the tertiary ultimate segment shape should be explicitly stated even if it is not observed. Additionally, the complete frond shape should always be explicitly stated, even in cases where it is not observed. In those cases, the characters of frond tapering at the tip and frond tapering at the base do not need to be explicitly stated. All character definitions in the complete schema describe the parameters in which the character state needs to be explicitly recorded.*

***5) Hypothesized Character States:*** A hypothesis may be included if a character state is proposed due to reasonable inference.

*Example:* A large pinnate penultimate segment is hypothesized to exhibit the division of the entire frond.

*Character:* Complete Blade Division

*Character State:* Complete Blade Division Not Observed But Likely Pinnate.

## ***Complete Description Formula***

**(1)** Preserved segment division (to the highest order observed), **(2)** complete blade division, **(3)** ultimate segment shape, **(4)** ultimate segment tapering at the tip, **(5)** ultimate segment tapering at the base, **(6)** ultimate segment symmetry, **(7)** penultimate segment shape, **(8)** penultimate segment tapering at the tip, **(9)** penultimate segment tapering at the base, **(10)** penultimate segment symmetry, **(11)** tertiary ultimate segment shape, **(12)** tertiary ultimate segment tapering at the tip, **(13)** tertiary ultimate segment tapering at the base, **(14)** quaternary ultimate segment shape, **(15)** quaternary ultimate segment tapering at the tip, **(16)** quaternary ultimate segment tapering at the base, **(17)** complete frond shape, **(18)** frond tapering at the tip, **(19)** frond tapering at the base, **(20)** vein type, **(21)** vein termination at the margin, **(22)** vein termination location, **(23)** margin type, **(24)** reproductive structure type/shape, **(25)** reproductive structure placement, and **(26)** presence of indusia/indusium type.

## ***Complete Description Schema with Defined Characters and Character States***

**Figure S2.2** Complete blade division and preserved segment division character states. This figure illustrates the terminology associated with the division of fern fronds. Black lines indicate the absence of leaf tissue between segments, resulting in discrete, completely separated segments (pinnate). Green-filled clefts without a black line indicate the presence of leaf tissue between segments where clefts do not completely separate them (pinnatifid). See definitions of each character state under *1. Preserved Segment Division* and 2. *Complete Blade Division.*

**1. Preserved Segment Division** – The degree of dissection of the frond up until what is preserved including if segment divisions extend to an axial vein (Figure S2.2). *Character state may be the same as the complete blade division if the complete blade division is observed or hypothesized. Character state should always be explicitly stated except when the complete blade division is known and observed.*

a. **Ultimate Segment Simple** – Ultimate segment is not further divided. Further division of the frond is unknown.
b. **Penultimate Segment Pinnate**– Penultimate segment is divided once into smaller segments. Ultimate segments are discrete, with clefts fully separating each ultimate segment.
c. **Penultimate Segment** **Pinnatifid** – Penultimate segment is divided once into smaller segments. Ultimate segments are not discrete, with clefts that do not completely separate each ultimate segment.
d. **Tertiary Ultimate Segment Pinnate-Pinnatifid** – Tertiary ultimate segment is divided twice into smaller segments. Penultimate segments are discrete, with clefts fully separating each penultimate segment. Ultimate segments are not discrete, with clefts that do not completely separate each ultimate segment.
e. **Tertiary Ultimate Segment Bipinnate** – Tertiary ultimate segment is divided twice into smaller segments. Ultimate and penultimate segments are discrete, with clefts fully separating each ultimate and penultimate segment.
f. **Tertiary Ultimate Segment Bipinnatifid** – Tertiary ultimate segment is divided twice into smaller segments. Ultimate and penultimate segments are not discrete, with clefts that do not completely separate each ultimate and penultimate segment.
g. **Quaternary Ultimate Segment** **Bipinnate-Pinnatifid** – Quaternary ultimate segment is divided three times into smaller segments. Penultimate and tertiary ultimate segments are discrete, with clefts fully separating each penultimate and tertiary ultimate segment. Ultimate segments are not discrete, with clefts that do not completely separate each ultimate segment.
h. **Quaternary Ultimate Segment** **Tripinnate** – Quaternary ultimate segment is divided three times into smaller segments. Ultimate, penultimate, and tertiary ultimate segments are discrete, with clefts fully separating each ultimate, penultimate, and tertiary ultimate segment.
i. **Quaternary Ultimate Segment** **Tripinnatifid**– Quaternary ultimate segment is divided three times into smaller segments. Ultimate, penultimate, and tertiary ultimate segments are not discrete, with clefts that do not completely separate each ultimate and penultimate segment.
j. **Segment Division Not Observed** – No segmentation of the frond is preserved.

**2. Complete Blade Division** – The degree of dissection of the frond including if segment divisions fully extend to an axial vein (Figure S2.2). *Character is only applicable when the entire frond is preserved.* *Character state should always be explicitly stated.*

a. **Blade Structure Simple**– Entire blade is not segmented.
b. **Blade Structure Pinnate** – Blade is divided once into smaller segments. Ultimate segments are discrete, with clefts fully separating each ultimate segment.
c. **Blade Structure Pinnatifid** – Blade is divided once into smaller segments. Ultimate segments are not discrete, with clefts that do not completely separate each ultimate segment.
d. **Blade Structure Pinnate-Pinnatifid** – Blade is divided twice into smaller segments. Penultimate segments are discrete, with clefts fully separating each penultimate segment. Ultimate segments are not discrete, with clefts that do not completely separate each ultimate segment.
e. **Blade Structure Bipinnate**– Blade is divided twice into smaller segments. Ultimate and penultimate segments are discrete, with clefts fully separating each ultimate and penultimate segment.
f. **Blade Structure Bipinnatifid** – Blade is divided twice into smaller segments. Ultimate and penultimate segments are not discrete, with clefts that do not completely separate each ultimate and penultimate segment.
g. **Blade Structure Bipinnate-Pinnatifid** – Blade is divided three times into smaller segments. Penultimate and tertiary ultimate segments are discrete, with clefts fully separating each penultimate and tertiary ultimate segment. Ultimate segments are not discrete, with clefts that do not completely separate each ultimate segment.
h. **Blade Structure** **Tripinnate** – Blade is divided three times into smaller segments. Ultimate, penultimate, and tertiary ultimate segments are discrete, with clefts fully separating each ultimate, penultimate, and tertiary ultimate segment.
i. **Blade Structure Tripinnatifid** – Blade is divided three times into smaller segments. Ultimate, penultimate, and tertiary ultimate segments are not discrete, with clefts that do not completely separate each ultimate and penultimate segment.
j. **Complete Blade Division Not Observed** – Entire blade is not preserved. A hypothesis may be included such as: *Complete blade division not observed but likely pinnate.*

**Figure S2.3** Character states for frond/segment shape: complete frond shape, quaternary ultimate segment shape, tertiary ultimate segment shape, penultimate segment shape, and ultimate segment shape. See *3. Ultimate Segment Shape, 7. Penultimate Segment Shape, 11. Tertiary Ultimate Segment Shape, 14. Quaternary Ultimate Segment Shape, and 17. Complete Frond Shape* characters for character state definitions.

**3. Ultimate Segment Shape** – Shape of the ultimate segment, ignoring gaps between lobe spacing (Figure S2.3). *If the penultimate segment is pinnatifid, the base of the ultimate segment is disregarded. Character state should always be explicitly stated, except in the case of a known simple frond.*

a. **Ultimate Segment Shape Ovate** – Egg-shaped segment that is broader toward the base and narrower toward the tip.
b. **Ultimate Segment Shape Obovate** – Egg-shaped segment that is broader toward the tip and narrower at the base.
c. **Ultimate Segment Shape Lanceolate** – Lance-shaped segment that is much longer than wide, broader toward the base, and narrower toward the tip.
d. **Ultimate Segment Shape Oblanceolate** – Lance-shaped segment that is much longer than wide, broader toward the tip, and narrower toward the base.
e. **Ultimate Segment Shape Oblong** – Segment is two to four times as long as it is wide with margins that run nearly parallel to the axial vein.
f. **Ultimate Segment Shape Elliptic** – Ellipse-shaped segment with the broadest portion in the middle and narrowing at the base and tip.
g. **Ultimate Segment Shape Orbicular** – Circular segment shape with equal or nearly equal width and length.
h. **Ultimate Segment Shape Flabellate** – Fan-shaped segment.
i. **Ultimate Segment Shape Irregular** – Segment does not have a consistent or defined shape.
j. **Ultimate Segment Shape Linear** – Long, narrow segment with nearly parallel margins, greater than 4 times as long as wide.
k. **Ultimate Segment Shape Not Observed** – Entire ultimate segment is not preserved.

**Figure S2.4** Segment/frond tapering at the base and tip character states. (A), (B) short tapering at the base/tip; (C) long tapering at the base/tip. See *4. Ultimate Segment Tapering at the Tip,* *5. Ultimate Segment Tapering at the Base, 8. Penultimate Segment Tapering at the Tip, 9. Penultimate Segment Tapering at the Base, 12. Tertiary Ultimate Segment Tapering at the Tip, 13. Tertiary Ultimate Segment Tapering at the Base, 15. Quaternary Ultimate Segment Tapering at the Tip, 16. Quaternary Ultimate Segment Tapering at the Base, 18. Frond Tapering at the Tip, and 19. Frond Tapering at the Base* characters for character state definitions.

**4. Ultimate Segment Tapering at the Tip** – The length of tapering at the apex of the ultimate segment (Figure S2.4). *Character state should always be explicitly stated, except in the case of a known simple frond.*

a. **Ultimate Segment Short Tapering at the Tip** – Ultimate segment abruptly narrows over a short distance to the tip of this segment. Tip of the ultimate segment is rounded or slightly pointed.
b. **Ultimate Segment Long Tapering at the Tip** – Ultimate segment gradually narrows over a long distance to the tip of this segment. Tip of the ultimate segment is pointed.
c. **Ultimate Segment Tapering at the Tip Not Observed** – Tip of the ultimate segment is not preserved.

**5. Ultimate Segment Tapering at the Base** – The length of tapering at the base of the ultimate segment (Figure S2.4). *Not applicable if the penultimate segment is pinnatifid. Character state should always be explicitly stated, except in the case of a known simple frond.*

a. **Ultimate Segment Short Tapering at the Base** – Ultimate segment abruptly narrows over a short distance to the base of this segment. Base of the ultimate segment is rounded or slightly pointed.
b. **Ultimate Segment Long Tapering at the Base** – Ultimate segment gradually narrows over a long distance to the base of this segment. Base of the ultimate segment is pointed.
c. **Ultimate Segment Tapering at the Base Not Observed** – Base of the ultimate segment is not preserved.
d. **Ultimate Segment Tapering at the Base Not Applicable** – Penultimate segment is pinnatifid and the ultimate segment does not taper at the base.

**Figure S2.5** Penultimate segment symmetry and ultimate segment symmetry character states. Segment symmetry indicates if segments are equal or unequal in size across an axial vein. Ultimate segment symmetry character states: (A) symmetrical ultimate segments; (B) asymmetrical ultimate segments. Penultimate segment symmetry character states: (C) symmetrical penultimate segments; (D) asymmetrical penultimate segments. See *6. Ultimate Segment Symmetry* and *10. Penultimate Segment Symmetry* for character state definitions.

**6. Ultimate Segment Symmetry** – Indicates if ultimate segments are equal or unequal in size across the penultimate segment axial vein (Figure S2.5). *Character state should always be explicitly stated.*

a. **Ultimate Segments** **Symmetrical** – Ultimate segments are equal in size across the penultimate segment axial vein.
b. **Ultimate Segments** **Asymmetrical** – Ultimate segments are unequal in size across the penultimate segment axial vein.
c. **Ultimate Segment Symmetry Not Observed** – Ultimate segment symmetry is not observed across the penultimate segment axial vein.
d. **Ultimate Segment Symmetry Not Applicable** – Entire frond has a simple blade division where the symmetry of ultimate segments cannot be assessed. (Usually also the case for pinnate and pinnatifid fronds).

**7. Penultimate Segment Shape** – Shape of the penultimate segment, ignoring gaps between lower order segment spacing (Figure S2.3). *If the tertiary ultimate segment is bipinnatifid, the base of the penultimate segment is disregarded. If the complete blade division or preserved segment division does not indicate the presence of this level of division, this character/character state does not need to be explicitly stated.*

a. **Penultimate Segment Shape** **Ovate** – Egg-shaped segment that is broader toward the base and narrower toward the tip.
b. **Penultimate Segment Shape** **Obovate** – Egg-shaped segment that is broader toward the tip and narrower at the base.
c. **Penultimate Segment Shape Lanceolate** – Lance-shaped segment that is much longer than wide, broader toward the base, and narrower toward the tip.
d. **Penultimate Segment Shape Oblanceolate** – Lance-shaped segment that is much longer than wide, broader toward the tip, and narrower toward the base.
e. **Penultimate Segment Shape** **Oblong** – Segment is two to four times as long as it is wide with margins that run nearly parallel to the axial vein.
f. **Penultimate Segment Shape Elliptic** – Ellipse-shaped segment with the broadest portion in the middle and narrowing at the base and tip.
g. **Penultimate Segment Shape Orbicular** – Circular segment shape with equal or nearly equal width and length.
h. **Penultimate Segment Shape Flabellate** – Fan-shaped segment.
i. **Penultimate Segment Shape Irregular** – Segment does not have a consistent or defined shape.
j. **Penultimate Segment Shape Linear** – Long, narrow segment with nearly parallel margins, greater than 4 times as long as wide.
k. **Penultimate Segment Shape Not Observed** – Entire penultimate segment is not preserved, but evidence of the penultimate segment level of division is present.

**8. Penultimate Segment Tapering at the Tip** – The length of tapering at the apex of the penultimate segment (Figure S2.4). *If the complete blade division or preserved segment division does not indicate the presence of this level of division, this character/character state does not need to be explicitly stated. If the penultimate ultimate segment shape is not preserved, this character state should still be explicitly stated.*

a. **Penultimate Segment Short Tapering at the Tip** – Penultimate segment abruptly narrows over a short distance to the tip of this segment. Tip of the penultimate segment is rounded or slightly pointed.
b. **Penultimate Segment Long Tapering at the Tip** – Penultimate segment gradually narrows over a long distance to the tip of this segment. Tip of the penultimate segment is pointed.
c. **Penultimate Segment Tapering at the Tip Not Observed** – Tip of the penultimate segment is not preserved, but the penultimate segment level of division is present.

**9. Penultimate Segment Tapering at the Base** – The length of tapering at the base of the penultimate segment (Figure S2.4). *Not applicable if the tertiary ultimate segment is bipinnatifid. If the complete blade division or preserved segment division does not indicate the presence of this level of division, this character/character state does not need to be explicitly stated. If the penultimate segment shape is not preserved, this character state should still be explicitly stated.*

a. **Penultimate Segment Short Tapering at the Base** – Penultimate segment abruptly narrows over a short distance to the base of this segment. Base of the penultimate segment is rounded or slightly pointed.
b. **Penultimate Segment Long Tapering at the Base** – Penultimate segment gradually narrows over a long distance to the base of this segment. Base of the penultimate segment is pointed.
c. **Penultimate Segment Tapering at the Base Not Observed** – Base of the penultimate segment is not preserved, but the penultimate segment level of division is present.
d. **Penultimate Segment Tapering at the Base Not Applicable** – Tertiary ultimate segment is bipinnatifid and the penultimate segment does not taper at the base.

**10. Penultimate Segment Symmetry** – Indicates if penultimate segments are equal or unequal in size across the tertiary ultimate axial vein (Figure S2.5). *If the complete blade division or preserved segment division does not indicate the presence of the penultimate segment, this character/character state does not need to be explicitly stated.*

a. **Penultimate Segments** **Symmetrical** – Penultimate segments are equal in size across the tertiary ultimate segment axial vein.
b. **Penultimate Segments Asymmetrical** – Penultimate segments are unequal in size across the tertiary ultimate segment axial vein.
c. **Penultimate Segment Symmetry Not Observed** – Penultimate segment symmetry is not observed across the tertiary ultimate segment axial vein.
d. **Penultimate Segment Symmetry Not Applicable** – Entire frond has a simple, pinnate, or pinnatifid blade division where the symmetry of penultimate segments cannot be assessed. (Usually also the case for bipinnate, pinnate-pinnatifid, and bipinnatifid fronds).

**11. Tertiary Ultimate Segment Shape** – Shape of the tertiary ultimate segment, ignoring gaps between lower order segment spacing (Figure S2.3). *If the quaternary ultimate segment is tripinnatifid, the base of the tertiary ultimate segment is disregarded. If the complete blade division or preserved segment division does not indicate the presence of this level of division, this character/character state does not need to be explicitly stated.*

a. **Tertiary Ultimate Segment Shape Ovate** – Egg-shaped segment that is broader toward the base and narrower toward the tip.
b. **Tertiary Ultimate Segment Shape Obovate** – Egg-shaped segment that is broader toward the tip and narrower at the base.
c. **Tertiary Ultimate Segment Shape Lanceolate** – Lance-shaped segment that is much longer than wide, broader toward the base, and narrower toward the tip.
d. **Tertiary Ultimate Segment Shape Oblanceolate** – Lance-shaped segment that is much longer than wide, broader toward the tip, and narrower toward the base.
e. **Tertiary Ultimate Segment Shape Oblong** – Segment is two to four times as long as it is wide with margins that run nearly parallel to the axial vein.
f. **Tertiary Ultimate Segment Shape Elliptic** – Ellipse-shaped segment with the broadest portion in the middle and narrowing at the base and tip.
g. **Tertiary Ultimate Segment Shape Orbicular** – Circular segment shape with equal or nearly equal width and length.
h. **Tertiary Ultimate Segment Shape Flabellate** – Fan-shaped segment.
i. **Tertiary Ultimate Segment Shape Irregular** – Segment does not have a consistent or defined shape.
j. **Tertiary Ultimate Segment Shape Linear** – Long, narrow segment with nearly parallel margins, greater than 4 times as long as wide.
k. **Tertiary Ultimate Segment Shape Not Observed** – Entire tertiary ultimate segment is not preserved, but evidence of the tertiary ultimate segment level of division is present.

**12. Tertiary Ultimate Segment Tapering at the Tip** – The length of tapering at the apex of the tertiary ultimate segment (Figure S2.4). *If the complete blade division or preserved segment division does not indicate the presence of this level of division, this character/character state does not need to be explicitly stated. If the tertiary ultimate segment shape is not preserved, this character state should still be explicitly stated.*

a. **Tertiary Ultimate Segment Short Tapering at the Tip** – Tertiary ultimate segment abruptly narrows over a short distance to the tip of this segment. Tip of the tertiary ultimate segment is rounded or slightly pointed.
b. **Tertiary Ultimate Segment Long Tapering at the Tip** – Tertiary ultimate segment gradually narrows over a long distance to the tip of this segment. Tip of the tertiary ultimate segment is pointed.
c. **Tertiary Ultimate Segment Tapering at the Tip Not Observed** – Tip of the tertiary ultimate segment is not preserved, but the tertiary ultimate segment level of division is present.

**13. Tertiary Ultimate Segment Tapering at the Base** – The length of tapering at the base of the tertiary ultimate segment (Figure S2.4). *Not applicable if the quaternary ultimate segment is tripinnatifid. If the complete blade division or preserved segment division does not indicate the presence of this level of division, this character/character state does not need to be explicitly stated. If the tertiary ultimate segment shape is not preserved, this character state should still be explicitly stated.*

a. **Tertiary Ultimate Segment Short Tapering at the Base** – Tertiary ultimate segment abruptly narrows over a short distance to the base of this segment. Base of the tertiary ultimate segment is rounded or slightly pointed.
b. **Tertiary Ultimate Segment Long Tapering at the Base** – Tertiary ultimate segment gradually narrows over a long distance to the base of this segment. Base of the tertiary ultimate segment is pointed.
c. **Tertiary Ultimate Segment Tapering at the Base Not Observed** – Base of the tertiary ultimate segment is not preserved, but the tertiary ultimate segment level of division is present.
d. **Tertiary Ultimate Segment Tapering at the Base Not Applicable** – Quaternary ultimate segment is tripinnatifid and the tertiary ultimate segment does not taper at the base.

**14. Quaternary Ultimate Segment Shape** – Shape of the quaternary ultimate segment, ignoring gaps between lower order segment spacing (Figure S2.3). *If the fifth-order ultimate segment is 4-pinnatifid, the base of the quaternary ultimate segment is disregarded. If the complete blade division or preserved segment division does not indicate the presence the quaternary ultimate segment, this character/character state does not need to be explicitly stated.*

a. **Quaternary Ultimate Segment Shape Ovate** – Egg-shaped segment that is broader toward the base and narrower toward the tip.
b. **Quaternary Ultimate Segment Shape Obovate** – Egg-shaped segment that is broader toward the tip and narrower at the base.
c. **Quaternary Ultimate Segment Shape Lanceolate** – Lance-shaped segment that is much longer than wide, broader toward the base, and narrower toward the tip.
d. **Quaternary Ultimate Segment Shape Oblanceolate** – Lance-shaped segment that is much longer than wide, broader toward the tip, and narrower toward the base.
e. **Quaternary Ultimate Segment Shape** **Oblong** – Segment is two to four times as long as it is wide with margins that run nearly parallel to the axial vein.
f. **Quaternary Ultimate Segment Shape** **Elliptic** – Ellipse-shaped segment with the broadest portion in the middle and narrowing at the base and tip.
g. **Quaternary Ultimate Segment Shape Orbicular** – Circular segment shape with equal or nearly equal width and length.
h. **Quaternary Ultimate Segment Shape Flabellate** – Fan-shaped segment.
i. **Quaternary Ultimate Segment Shape Irregular** – Segment does not have a consistent or defined shape.
j. **Quaternary Ultimate Segment Shape Linear** – Long, narrow segment with nearly parallel margins, greater than 4 times as long as wide.
k. **Quaternary Ultimate Segment Shape Not Observed** – Entire quaternary ultimate segment is not preserved, but evidence of the quaternary ultimate segment level of division is present.

**15. Quaternary Ultimate Segment Tapering at the Tip** – The length of tapering at the apex of the quaternary ultimate segment (Figure S2.4). *If the complete blade division or preserved segment division does not indicate the presence of this level of division, this character/character state does not need to be explicitly stated. If the quaternary ultimate segment shape is not preserved, this character state should still be explicitly stated.*

a. **Quaternary Ultimate Segment Short Tapering at the Tip** – Quaternary ultimate segment abruptly narrows over a short distance to the tip of this segment. Tip of the quaternary ultimate segment is rounded or slightly pointed.
b. **Quaternary Ultimate Segment Long Tapering at the Tip** – Quaternary ultimate segment gradually narrows over a long distance to the tip of this segment. Tip of the quaternary ultimate segment is pointed.
c. **Quaternary Ultimate Segment Tapering at the Tip Not Observed** – Tip of the quaternary ultimate segment is not preserved, but the quaternary ultimate segment level of division is present.

**16. Quaternary Ultimate Segment Tapering at the Base** – The length of tapering at the base of the quaternary ultimate segment (Figure S2.4). *Not applicable if the fifth-order ultimate segment is 4-pinnatifid. If the complete blade division or preserved segment division does not indicate the presence of this level of division, this character/character state does not need to be explicitly stated. If the quaternary ultimate segment shape is not preserved, this character state should still be explicitly stated.*

a. **Quaternary Ultimate Segment Short Tapering at the Base** – Quaternary ultimate segment abruptly narrows over a short distance to the base of this segment. Base of the quaternary ultimate segment is rounded or slightly pointed.
b. **Quaternary Ultimate Segment Long Tapering at the Base** – Quaternary ultimate segment gradually narrows over a long distance to the tip of this segment. Base of the quaternary ultimate segment is pointed.
c. **Quaternary Ultimate Segment Tapering at the Base Not Observed** – Base of the quaternary ultimate segment is not preserved, but the quaternary ultimate segment level of division is present.
d. **Quaternary Ultimate Segment Tapering at the Base Not Applicable** – Fifth-order ultimate segment is 4-pinnatifid and the quaternary ultimate segment does not taper at the base.

**17. Complete Frond Shape** – Shape of the entire frond, ignoring gaps between segment spacing (Figure S2.3). *Character is only applicable when the entire frond is preserved. Character state should always be explicitly stated.*

a. **Frond Shape Ovate** – Egg-shaped frond that is broader toward the base and narrower toward the tip.
b. **Frond Shape Obovate** – Egg-shaped frond that is broader toward the tip and narrower at the base.
c. **Frond Shape Lanceolate** – Lance-shaped frond that is much longer than wide, broader toward the base, and narrower toward the tip.
d. **Frond Shape Oblanceolate** – Lance-shaped frond that is much longer than wide, broader toward the tip, and narrower toward the base.
e. **Frond Shape Oblong** – Frond is two to four times as long as it is wide with margins that run nearly parallel to the axial vein.
f. **Frond Shape** **Elliptic** – Ellipse-shaped frond with the broadest portion in the middle and narrowing at the base and tip.
g. **Frond Shape** **Orbicular** – Circular frond shape with equal or nearly equal width and length.
h. **Frond Shape Flabellate** – Fan-shaped frond.
i. **Frond Shape Irregular** – Frond does not have a consistent or defined shape.
j. **Frond Shape Linear** – Long, narrow frond with nearly parallel margins, greater than 4 times as long as wide.
k. **Complete Frond Shape Not Observed** – Entire frond is not preserved.

**18. Frond Tapering at the Tip** – The length of tapering at the apex of the entire frond (Figure S2.4). *If the complete blade division is not preserved, this character/character state does not need to be explicitly stated.*

a. **Frond Short Tapering at the Tip** – Frond abruptly narrows over a short distance to the tip of the frond. Tip of the frond is rounded or slightly pointed.
b. **Frond Long Tapering at the Tip** – Frond gradually narrows over a long distance to the tip of the frond. Tip of the frond is pointed.
c. **Frond Tapering at the Tip Not Observed** – Apex of the frond is not preserved.

**19. Frond Tapering at the Base –** The length of tapering at the base of the entire frond (Figure S2.4). *If the complete blade division is not preserved, this character/character state does not need to be explicitly stated.*

a. **Frond Short Tapering at the Base** – Frond abruptly narrows over a short distance to the base of the frond. Base of the frond is rounded or slightly pointed.
b. **Frond Long Tapering at the Base** – Frond gradually narrows over a long distance to the base of the frond. Base of the frond is pointed.
c. **Frond Tapering at the Base Not Observed** – Base of the frond is not preserved.

**Figure S2.6** Vein type character states: free simple venation, free forked venation, and anastomosing venation. See *20. Vein Type* for character state definitions.

**20. Vein Type** – The branching pattern of lateral veins (Figure S2.6). *Character state should always be explicitly stated.*

a. **Venation** **Free** – Lateral veins diverge from primary veins and do not reconnect.
   i. **Venation Free and Simple** – Lateral veins do not split into two or more branches.
   ii. **Venation Free and Forked** – Lateral veins split into two or more branches
b. **Venation** **Anastomosing** – Lateral veins branch and reconnect, forming a net-like structure.
c. **Venation Type Not Observed** – Lateral veins are not preserved.

**Figure S2.7** Vein termination at the margin character states. (A) Veins terminate at the margin; (B) veins do not terminate at the margin. See *21. Vein Termination at the Margin* for character state definitions. 

**21. Vein Termination at the Margin** – Indicates if the veins extend to the margin or terminate before reaching it (Figure S2.7). *If the vein type is not observed, this character/character state does not need to be explicitly stated.*

a. **Veins Terminate at the Margin** – Veins extend completely to the margin.
b. **Veins do not Terminate at the Margin** – Veins terminate before reaching the margin.
c. **Vein Termination at the Margin Not Observed** – Lateral venation and/or vein termination not preserved.

**Figure S2.8** Vein termination location character states. (A) Vein termination in lobes/segments only; (B), (C) vein termination in lobes/segments and clefts between segments. Green indicates the margin and black indicates veins. See *22. Vein Termination Location* for character state definitions.

**22. Vein Termination Location** – Indicates whether veins only end in lobes/segments or veins terminate in lobes/segments and clefts between segments (Figure S2.8). *Only applicable if the penultimate segment is pinnatifid/ultimate segment is lobed, but still should be explicitly stated*. *If the vein type is not observed, this character/character state does not need to be explicitly stated.*

a. **Vein Termination in Lobes/Segments Only** – Veins only terminate in ultimate segments/lobes.
b. **Vein Termination in Lobes/Segments and Clefts Between Segments** – Veins terminate in ultimate segments/lobes, and veins terminate in clefts between ultimate segments/lobes.
c. **Vein Termination in Lobes/Segments and Clefts Between Segments Not Observed** – In a penultimate segment that is pinnatifid or ultimate segment that is lobed, lateral venation and/or vein termination is not preserved.
d. **Vein Termination in Lobes/Segments and Clefts Between Segments Not Applicable** – Penultimate segment is pinnate or ultimate segment is not lobed.

**Figure S2.9** Margin type character states. See *23. Margin Type* for character state definitions.

**23. Margin Type** – The structure of the frond’s edge (Figure S2.9). *Character state should always be explicitly stated.*

a. **Margin Entire** – Frond margins are smooth.
b. **Margin Crenate** – Frond margins have rounded teeth.
c. **Margin Serrate** – Frond margins have pointed teeth.
d. **Margin** **Irregular** – Frond margins do not follow a consistent or defined pattern.
e. **Margin Type Not Observed** – Margin is not preserved.

**Figure S2.10.1** Reproductive structure type/shape: sori and sporangial groupings shape character states. (A) Round sori; (B) kidney bean shaped sori; (C) elongated sori; (D) j-shaped sori; and (E) linear sporangia. See *24. Reproductive Structure Shape/Type* for character state definitions.

**Figure S2.10.2** Reproductive structure type/shape: sporophore and sporocarp character states. (A) Sporophore branched and panicle-like; (B) sporophore spike-like; (C) sporocarps round. See *24. Reproductive Structure Shape/Type* for character state definitions. 

**24. Reproductive Structure Type/Shape** – Indicates spores are located in sori, sporophores, sporocarps, or only in sporangia (Figures S2.10.1 and S2.10.2). This includes the shape of the reproductive structures. *Character state should always be explicitly stated.*

a. **Sori** – Clusters of sporangia on the leaf tissue.
   i. **Sori** **Round** – Sori are circular in shape.
   ii. **Sori Kidney Bean Shaped** – Sori have a curved, bean-like shape.
   iii. **Sori J-Shaped** – Sori form the letter “J” in shape.
   iv. **Sori Elongated** – Sori are much longer than they are wide in shape.
   v. Sori Cone-Shaped – Sori are triangular and form a cone.
b. **Sporangial Groupings** – Structures that form and release spores.
   i. **Sporangia Linear** – Sporangia are not arranged into clusters, but instead follow a long continuous line (usually along margins or around an axial vein).
c. **Sporophore** – Sporangia and spores are in reduced, modified blades such as in Ophioglossaceae.
   i. **Sporophore Spike-like** – Sporophore is upright as simple, unbranched spike such as in *Ophioglossum*.
   ii. **Sporophore Branched and Panicle-like** – Sporophore branches and bears round sporangia, resembling a bunch of grapes such as in *Botrychium*.
d. **Sporocarps** – Modified structures derived from leaves or pinnae containing modified sori that produce both megaspores and microspores, present in aquatic ferns.
   i. **Sporocarps Round** – Sporocarps form the shape of a sphere or are nearly spherical.
   ii. **Sporocarps Bean-Shaped** – Sporocarps form the shape of a bean that is elongated and slightly curved.
   iii. **Sporocarps Irregular/Clustered** – Sporocarps do not have a uniform shape and appear to be closely-packed structures.
e. **Reproductive Structures Not Observed** – Reproductive structures not preserved. A hypothesis may be included such as: *Reproductive structures not observed but likely sporocarps.*

**25. Reproductive Structure Placement** – Indicates where reproductive structures are located. *If reproductive structures are not preserved, this character/character state does not need to be explicitly stated.*

a. **Sori/Sporangia**
   i. **Sori Scattered on Segments** – Sori are irregularly distributed on the leaf tissue. Can also be refined for clarity or stated like: *Sori scattered on ultimate segments.*
   ii. **(Sori/Sporangia) Along the Margins** – Sori or sporangia are directly on the margins or closely follow the margins.
       i. **Sori Discrete and not Continuous on Margin** – Sori are along the margins, but are not closely spaced together, where there is visible leaf tissue between each sorus.
       ii. **Sori Continuous and Linearly Arranged on Margin** – Sori are along the margins and are closely spaced together and there is no visible leaf tissue between each sorus.
       iii. **Sporangia on the Margin** - Sporangia are continuous along the margins and are not congregated into discrete sori.
   iii. **(Sori/Sporangia) Along the Sides of the Axial Veins** – Sori or sporangia surround both sides of an axial vein. Can also be refined for clarity or stated like: *Sori along the sides of the ultimate and penultimate segment axial veins; Sori along the sides of the ultimate segment axial veins*.
   iv. **Sori Centered Between Axial Vein and Margin** – Sori are centered or near to the center of a segment between an axial vein and the margin. Can also be refined for clarity or stated like: *Sori centered between ultimate segment axial vein and margin.*
   v. **Sori on a Dimorphic Frond Lacking Herbaceous Tissue** – Sori are on a fertile frond that lacks photosynthetic tissue.
b. **Sporophores**
   i. **Sporophores Attached at the Base of Sterile (Frond/Segment)** – Sporophores as a modified fertile frond/segment diverge at the base of the sterile frond/segment.
c. **Sporocarps**
   i. **Sporocarps Attached to Rhizomes at the Base of the Frond** – Sporocarps develop from rhizomes as the base of the vegetative frond.

**Figure S2.11** Presence of indusia/indusium type character states. (A) Absent indusia; (B) true indusia; and (C) false indusia. Small circles indicate sporangia, yellow indicates indusia (B) or false indusia (C), and green indicates unspecialized leaf tissue. See *26. Presence of Indusia/Indusium Type* for character state definitions.

**26. Presence of Indusia/Indusium Type** – Indicates if indusia are observed and the type that is observed (Figure S2.11). *If reproductive structures are not preserved, this character/character state does not need to be explicitly stated.*

a. **True Indusia** – Specialized tissue that protects the sori.
b. **False Indusia** – Margin of the frond rolls over the sori for protection, but is not specialized tissue.
c. **Absent Indusia** – No indusium present, but sori are present.
d. **Indusia Not Observed** – Sori, sporangia, or sporocarps are observed, but indusia are not observed and their character state cannot be inferred.
